# Anti-Inflammatory and Antioxidant Strategies in Epilepsy: From Molecular Mechanisms to Threshold Management

**DOI:** 10.3390/ijms27156606

**Published:** 2026-07-24

**Authors:** Alexander Trofimov, Ksenia Shcherbakova, Alexander Schwarz, Burkitkan Akbay, Orynbassar Karapina, Zhuldyz Myrkhiyeva, Egor Shirokov, Bauyrzhan Kizatov, Kudiyar Zhukanov, Ayaulym Baktursyn, Madina Isseyeva, Aliya Namiyaliyeva, Alexey Sarapultsev, Maria Komelkova, Oleg Lookin, Tursonjan Tokay

**Affiliations:** 1 Laboratory of Neurophysiology, Department of Biology, School of Sciences and Humanities (SSH), Nazarbayev University, 53 Kabanbay Batyr Ave., Astana 010000, Kazakhstan; burkitkan.akbay@nu.edu.kz (B.A.); orynbassar.karapina@nu.edu.kz (O.K.); zhuldyz.myrkhiyeva@nu.edu.kz (Z.M.); egor.shirokov@nu.edu.kz (E.S.); bauyrzhan.kizatov@nu.edu.kz (B.K.); kudiyar.zhukanov@nu.edu.kz (K.Z.); ayaulym.baktursyn@nu.edu.kz (A.B.); madina.isseyeva@nu.edu.kz (M.I.); aliya.namiyaliyeva@nu.edu.kz (A.N.); 2 Laboratory of Neurobiology of Integrative Brain Functions, I.P. Pavlov Department of Physiology, Institute of Experimental Medicine, 12 Acad. Pavlov St., 197022 St. Petersburg, Russia; 3 Independent Researcher, Montreal, QC H3Y 2W4, Canada; shcherbakova.ksenia.jp@gmail.com; 4 Laboratory of Molecular Mechanisms of Neuronal Interactions, I.M. Sechenov Institute of Evolutionary Physiology and Biochemistry, Russian Academy of Sciences, 44 Thorez Ave., 194223 St. Petersburg, Russia; aleksandr.pavlovich.schwarz@gmail.com; 5 Institute of Immunology and Physiology, Ural Branch of the Russian Academy of Sciences, 106 Pervomaiskaya St., 620049 Ekaterinburg, Russia; a.sarapultsev@gmail.com; 6 Russian–Chinese Education and Research Center of System Pathology, South Ural State University, 76 Lenin Prospekt, 454080 Chelyabinsk, Russia; mkomelkova@mail.ru; 7 JSC “National Scientific Medical Center”, 42 Abylai Khan Ave., Astana 010009, Kazakhstan; lookinoleg@gmail.com

**Keywords:** epilepsy, drug-resistant epilepsy (DRE), antiseizure medications (ASMs), seizure threshold, neuroinflammation, oxidative stress (OS), mitochondrial dysfunction, blood–brain barrier (BBB), ketogenic diet (KD), precision medicine

## Abstract

Epilepsy is a multifactorial disorder, yet routine management still focuses on neuronal excitation and insufficient inhibition, with antiseizure medications (ASMs) as the primary therapeutic strategy. This approach fails in roughly one-third of patients who develop drug-resistant epilepsy (DRE). Converging evidence links DRE with neuroinflammation, oxidative stress (OS), and mitochondrial dysfunction—an interconnected distal pathophysiological triad that progressively lowers seizure thresholds yet remains peripheral to clinical epilepsy management. We map this triad mechanistically and show that ASMs modulate it beyond their anticonvulsant activity, while triad-targeting pharmacological, dietary, and botanical interventions independently reduce seizure susceptibility. Common precipitants are reinterpreted as acute activators of the distal triad, linking precipitant identification and patient agency to threshold elevation. Integrating these elements, we propose a threshold management framework for DRE, built on a revised reservoir model, and translate it into three structural priorities: mechanistic phenotyping to stratify patients by pathophysiological domain, dual-mechanism drug development, and trial designs suited to multicomponent, context-dependent interventions. Together, these proposals reframe epilepsy management from sequential pharmacological trials toward coordinated optimization of the full seizure threshold landscape.

## 1. Part 1: Distal Pathophysiological Mechanisms in Epilepsy

### 1.1. Epilepsy as a Seizure Threshold Disorder

Epilepsy is one of the most prevalent neurological disorders, affecting approximately 50 million people worldwide [1,2]. Conceptually, an epileptic seizure is defined as a transient occurrence of signs and/or symptoms due to abnormal, excessive, or synchronous neuronal activity in the brain, whereas epilepsy denotes an enduring predisposition to generate such seizures, including their neurobiological, cognitive, psychological, and social consequences [3]. This enduring predisposition, in turn, can be conceptualized as a pathologically lowered seizure threshold—a state in which multiple causal factors converge to increase the probability of recurrent seizures [3]. Most currently available antiseizure medications (ASMs) address this predisposition at the level of neural excitability, reducing seizure probability by modulating ion channels and neurotransmitter systems [4,5].

Current diagnostic framework in epilepsy explicitly acknowledges the multifactorial nature of the disease. The International League Against Epilepsy (ILAE) recommends a stepwise classification proceeding from seizure type through epilepsy type to syndromic diagnosis [6,7], with parallel etiologic assignment across structural, genetic, infectious, metabolic, immune, and unknown categories. Etiology is explicitly recognized as predictive of prognosis, comorbidities, and the likely relevance of non-neuronal mechanisms such as immune signaling, blood–brain barrier (BBB) dysfunction, or metabolic factors [6]. Yet in routine clinical practice, in most cases, the etiologic category shapes the choice of ASM rather than lead to questioning whether the use of ASM is the right therapeutic strategy.

ASM monotherapy remains the cornerstone of initial management, achieving seizure freedom in approximately 50% of newly diagnosed patients with the first agent and an additional 11% to 12% with the second regimen [8,9]. However, approximately one-third of patients develop drug-resistant epilepsy (DRE) despite multiple therapeutic trials [10,11]. The ILAE defines DRE as “failure of adequate trials of two tolerated and appropriately chosen and used ASM schedules (whether as monotherapies or in combination) to achieve sustained seizure freedom” [10]. Despite the introduction of more than a dozen new ASMs with diverse mechanisms of action over the past three decades, long-term treatment outcomes have changed little at the population level [9]. Moreover, drug-induced seizure exacerbation further complicates ASM management, as certain agents may paradoxically worsen seizure control in susceptible individuals [12,13], suggesting that continued ASM development focused only on stronger seizure suppression is unlikely to fully meet the needs of the substantial proportion of patients whose epilepsy remains uncontrolled.

#### 1.1.1. Multifactorial Causation and the Proximal-Distal Divide

The threshold concept, central to how we think about seizures nowadays, was historically introduced as a latent instability that becomes clinically manifest when precipitating influences act on a susceptible brain. These contributors, progressively clarified over time, originally included genetic predispositions, acquired structural lesions, metabolic derangements, and transient precipitating factors [14,15,16]. William G. Lennox (1884–1960) illustrated this view by a reservoir analogy, which we will return to in Part 3 [17]. In this analogy, multiple contributory factors act as converging tributaries whose cumulative effects eventually reach the point at which neuronal hyperexcitability manifests as clinical seizure activity [15,18].

Thinking about epilepsy as a multifactorial disease also benefits from distinguishing between proximal and distal causal factors [15,19]. Proximal causes represent the immediate cellular and network disturbances at the epileptic focus, including ion channel dysfunction, neurotransmitter imbalance, and pathological neuronal synchronization, that is, the mechanisms primarily targeted by currently available ASMs, while distal causes, in contrast, encompass the upstream structural, metabolic, inflammatory, and other processes that create a permissive environment for seizure generation, including genetic mutations, acquired brain lesions, neuroinflammation, oxidative stress (OS), and mitochondrial dysfunction [15,16,19]. As emphasized by Shorvon, where there is least knowledge is in understanding how the known structural diseases, that is distal causes, give rise to the molecular changes in epilepsy, that is proximal causes [15]. Thus, standard ASMs act mainly on proximal mechanisms, suppressing abnormal electrical activity while often leaving insufficiently addressed the distal factors that maintain chronically low seizure thresholds.

It should be noted that the terminology used to describe epilepsy pharmacotherapy has evolved significantly in recent years. Historically, terms such as “antiepileptic drugs” (AEDs) and “anticonvulsants” have been widely used. The ILAE now recommends the term “antiseizure medications” (ASMs), explicitly framing currently approved agents as symptomatic rather than disease-modifying [4]. This shift appears not to have been fully absorbed by the field: a 2024 survey in epileptologists and epilepsy patients found that 56.42% of respondents still favored the terms “antiepileptic drug”, “AED”, and “antiepileptic therapy”, and only 17.32% endorsed the ILAE terminology [20]. We adopt “antiseizure medications” throughout because this framing is central to the threshold-based argument developed below.

#### 1.1.2. Proximal Mechanisms of Seizure Generation: Excitatory/Inhibitory Imbalance

Before turning to the distal mechanisms, which are the main focus of this review, we briefly outline the proximal mechanisms of seizure generation.

Seizures arise when excitatory drive exceeds inhibitory restraint, producing hypersynchrony and sustained firing across neuronal ensembles [21,22,23,24,25]. On the excitatory side, extracellular glutamate is elevated in the epileptogenic tissues, rising before and during spontaneous seizures [25,26], with chronically elevated interictal tone [27]. This hyperglutamatergic state is reinforced by altered receptor function, particularly in α-amino-3-hydroxy-5-methyl-4-isoxazolepropionic acid receptor (AMPAR) and N-methyl-D-aspartate receptor (NMDAR) subunits [28], as well as impaired astrocytic clearance, glutamine synthetase deficiency, and region-specific excitatory amino acid transporter (EAAT1/EAAT2) redistribution that collectively favor glutamate accumulation [24,29,30,31]. In status epilepticus (SE), progressive internalization of synaptic GABA_A_Rs alongside upregulated NMDAR and AMPAR signaling further promotes pharmacoresistance and ongoing excitotoxicity [22]. That impaired glutamate clearance is therapeutically actionable is supported by preclinical evidence that allosteric enhancement of EAAT2-mediated glutamate uptake suppresses seizures across several animal models [32].

On the inhibitory side, GABAergic underfunction in epilepsy manifests in interneuron loss, synaptic reorganization, receptor-subunit remodeling that alters receptor kinetics and phasic inhibition [33,34,35,36,37,38], and impaired chloride extrusion: when the K^+^-Cl^−^ cotransporter KCC2 is downregulated or the NKCC1/KCC2 balance shifts unfavorably, GABA signaling can convert from hyperpolarizing to depolarizing, facilitating synchronization and epileptiform discharge [21,23]. Potentiating KCC2 activity limits seizure onset and severity in vivo [39,40,41,42,43].

These are the immediate electrophysiological pathways typically targeted by ASMs, and the proximal interface through which distal pathophysiological processes express themselves clinically.

#### 1.1.3. The Distal Triad—Neuroinflammation, Oxidative Stress, and Mitochondrial Dysfunction—As High-Yield Targets in Drug-Resistant Epilepsy

The proximal/distal distinction is particularly important in DRE, as after the failure of two appropriately chosen ASM regimens, continued emphasis on proximal mechanisms alone yields diminishing returns. For example, in the 30-year Glasgow cohort, a third ASM regimen provided only an additional 4.4% likelihood of 1-year seizure freedom [9]. At this stage, greater attention to distal causal factors becomes a rational therapeutic shift.

Among the many candidate distal mechanisms, three pathophysiological domains—neuroinflammation, oxidative stress, and mitochondrial dysfunction—stand out because of their broad relevance across epilepsy syndromes, their mechanistic interdependence, and their accessibility to therapeutic modulation, forming a “distal triad,” as we refer to it in the present review.

Briefly, neuroinflammation includes chronic glial activation and cytokine signaling, which can directly enhance neuronal excitability, impair inhibitory neurotransmission, and compromise BBB integrity [44,45]; oxidative stress (OS) refers to the accumulation of reactive oxygen and nitrogen species that damage cellular membranes, proteins, and nucleic acids, while also activating redox-sensitive signaling pathways, including NF-κB [46,47]; mitochondrial dysfunction includes impaired oxidative phosphorylation, reduced ATP availability for maintenance of ionic gradients and synaptic transmission, excessive mitochondrial reactive oxygen species (ROS) production, and release of mitochondrial danger signals that amplify inflammatory cascades [48,49]. Importantly, these three domains are not parallel abnormalities acting independently but form a tightly connected pathogenic system. Through reciprocal interactions, each process reinforces the others and progressively stabilizes a brain state permissive for seizure generation. Moreover, seizures both arise from and intensify each component of the distal triad. Neuroinflammation, OS, and mitochondrial dysfunction therefore operate as both causes and consequences of epileptic activity [45,46,48,50]. What may begin as an acute response to seizure activity can therefore evolve into a chronic vulnerability that supports further seizures and, over time, epileptogenesis and pharmacoresistance. We review these mechanisms in detail in the following Section 1.3, Section 1.4, Section 1.5 and Section 1.6.

Given this bidirectional coupling with seizure activity, the distal triad represents a high-yield therapeutic target space in DRE. These mechanisms are widespread across diverse etiologies, lie upstream of many proximal disturbances targeted by conventional ASMs, remain dynamically coupled to seizure activity across different stages of disease, and are amenable to both pharmacological and non-pharmacological intervention.

### 1.2. Scope and Objectives: An Integrative Framework for Seizure Threshold Management

The mechanisms addressed in this review—neuroinflammation, OS, and mitochondrial dysfunction—have been the subject of a substantial prior literature, and existing reviews addressed inflammatory mediators and glial contributions [44,45,51,52,53,54,55,56,57,58,59,60,61,62], OS and mitochondrial bioenergetics [46,63,64,65,66,67,68,69,70], BBB dysfunction, peripheral immune entry as epileptogenic drivers [71,72,73,74,75,76], and broader integrative frameworks emphasizing redox-inflammation crosstalk and downstream therapeutic implications [47,77,78,79,80,81,82] (Table 1). The most closely related recent syntheses, Madireddy and Madireddy (2023), covered OS, mitochondrial dysfunction, neuroinflammation, BBB dysfunction, and a broad range of therapeutic approaches in a single manuscript [82]. The present review extends this literature in two respects. First, by placing the distal triad within an explicitly threshold-based framework, treating these mutually interacting processes as modulators of seizure susceptibility, pharmacoresistance, and long-term disease progression rather than as parallel pathological features. Second, it develops a translational framework integrating precipitating factors, patient agency, mechanistic phenotyping, and trial design that addresses the structural barriers currently preventing threshold management from reaching clinical practice in DRE.

This review thus adopts an explicitly integrative approach, examining neuroinflammation, OS, and mitochondrial dysfunction as interconnected processes, with emphasis on their mechanistic convergence, therapeutic accessibility, and clinical translation. Our specific objectives are:

1. To elucidate the molecular mechanisms linking neuroinflammation, OS, and mitochondrial dysfunction in epilepsy, emphasizing their roles as distal causal factors that maintain low seizure thresholds and systematically analyzing their bidirectional relationships among each other and with seizure activity.
2. To analyze the anti-inflammatory, antioxidant (AOX), and mitochondria-modulating properties of existing ASMs (Table S1), recognizing that these medications may exert therapeutically relevant effects beyond their primary ion channel or neurotransmitter targets, and that some agents may conversely aggravate inflammatory or oxidative burden, potentially contributing to treatment failure.
3. To review the antiseizure, neuroprotective, and, where supported, disease-modifying effects of existing anti-inflammatory drugs (AIDs) and AOX agents—including pharmacological agents, dietary modifications, targeted supplementation, and botanical approaches—in epilepsy and selected clinical contexts (Table S2).
4. To develop a unified threshold management framework integrating distal pathophysiology, precipitating factors, and patient agency; and to examine the structural barriers that currently impede its clinical translation, with particular focus on DRE.

By shifting the emphasis from seizure suppression to seizure threshold management, we propose a framework in which optimal epilepsy treatment, particularly in drug-resistant cases, combines conventional ASMs with pharmacological and non-pharmacological interventions directed at the inflammatory, oxidative, and metabolic vulnerabilities that maintain chronically low seizure thresholds. Targeting the distal triad in this context may serve disease-modifying and, where feasible, antiepileptogenic goals, slowing progression and reducing the biological conditions that sustain epileptic network instability, rather than limiting treatment to the suppression of established seizures.

It should be emphasized, however, that the strength of evidence varies substantially across mechanisms and interventions. In this review, “disease-modifying potential” is used cautiously, because in most cases the evidence remains preclinical or context-dependent. Confirmation of clinically meaningful disease modification in DRE will require biomarker-stratified clinical studies with long-term seizure outcomes and appropriate safety monitoring, which we address in detail in Part 3.

As for source selection, this article is a narrative, mechanistic-translational review and was not designed as a systematic review or meta-analysis. Relevant literature was identified through PubMed/MEDLINE and Google Scholar searches performed up to March 2026. Search terms included combinations of “epilepsy,” “drug-resistant epilepsy,” “seizure threshold,” “epileptogenesis,” “neuroinflammation,” “oxidative stress,” “mitochondrial dysfunction,” “blood–brain barrier,” “glia,” “cytokines,” “NLRP3,” “HMGB1,” “TLR4,” “antiseizure medications,” “anti-inflammatory drugs,” “antioxidants,” “ketogenic diet,” “supplements,” and “botanicals.” Priority was given to peer-reviewed primary studies, clinical trials or observational studies, and recent mechanistic reviews directly related to the inflammatory-oxidative-mitochondrial triad or to broader seizure threshold modulation and epilepsy management strategies. Publications were excluded when they were not directly relevant to epilepsy, seizure susceptibility, epileptogenesis, DRE, or the interventions discussed, or when they did not provide mechanistic, translational, clinical, or safety information relevant to the scope of the review. To maintain focus on mechanistic classification rather than comprehensive efficacy cataloging in Part 2, positive, inconsistent, and null findings are noted selectively within therapeutic subsections; a more systematic treatment of evidence limitations, failed trials, and the translational gap is developed in Part 3.

### 1.3. Neuroinflammation and Its Association with Epilepsy

**Figure 1:**
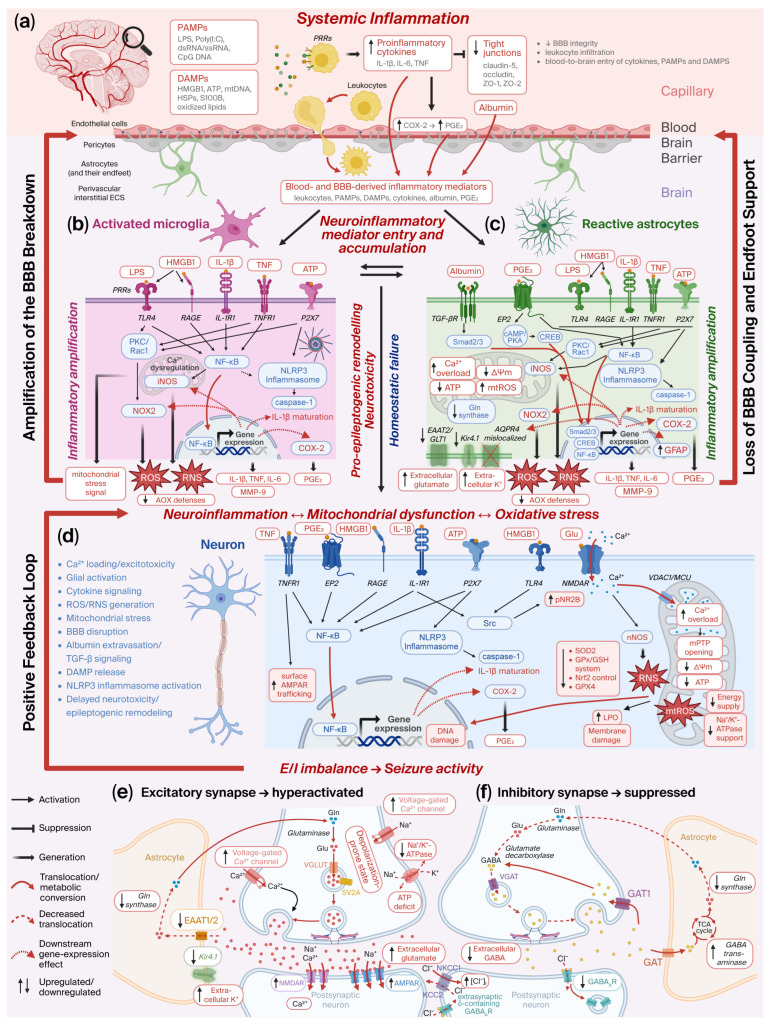
Crosstalk of inflammation, mitochondrial dysfunction, and OS with E/I synaptic balance. (**a**) Systemic inflammation compromises BBB integrity and promotes entry of blood-borne inflammatory mediators into the brain. PAMPs, DAMPs, circulating cytokines, albumin, PGE_2_, and leukocytes interact with the cerebrovascular unit, reduce tight-junction integrity, and facilitate leukocyte infiltration and brain exposure to systemic inflammatory signals. (**b**) Activated microglia amplify neuroinflammation through pattern-recognition and cytokine-responsive pathways, including TLR4, RAGE, IL-1R1, TNFR1, and P2X7 signaling, with downstream activation of NF-κB, iNOS, NOX2, the NLRP3 inflammasome, caspase-1, and COX-2. These pathways increase ROS and RNS, promote IL-1β maturation, and enhance release of cytokines, MMP-9, and PGE_2_, thereby reinforcing inflammatory amplification and neurotoxicity. (**c**) Reactive astrocytes lose their homeostatic function and acquire a pro-inflammatory phenotype. Albumin-TGF-β receptor signaling, PGE_2_-EP2 signaling, and cytokine- or DAMP-mediated activation of TLR4, RAGE, IL-1R1, TNFR1, and P2X7 converge on NF-κB-, COX-2-, iNOS-, and inflammasome-related pathways. In parallel, astrocytic mitochondrial dysfunction and OS develop, while glutamine synthetase, EAAT1/2-mediated glutamate uptake, Kir4.1-dependent K^+^ buffering, and AQP4 organization are impaired or mislocalized, contributing to extracellular glutamate and K^+^ accumulation and to loss of BBB-endfoot coupling. (**d**) Neurons integrate inflammatory and metabolic stress signals through EP2, RAGE, IL-1R1, TNFR1, P2X7, TLR4, and NMDAR pathways. IL-1β and HMGB1 signaling enhance excitatory drive through TLR4/IL-1R1-dependent facilitation of NMDAR Ca^2+^ influx, including Src- and NR2B-linked mechanisms, while TNF promotes excitatory receptor gain. Downstream consequences include NF-κB activation, COX-2/PGE_2_ signaling, nNOS-dependent RNS generation, mitochondrial Ca^2+^ overload, permeability transition pore opening, loss of ΔΨm, ATP depletion, impaired Na^+^/K^+^-ATPase activity, LPO, and DNA/membrane damage. Endogenous AOX defenses, including SOD2, the GPx/GSH system, Nrf2-regulated responses, and GPX4-dependent lipid peroxide control, become insufficient under persistent stress. (**e**) Excitatory synapses become hyperactivated owing to impaired astrocytic glutamate clearance and K^+^ buffering, increased extracellular glutamate, enhanced NMDAR- and AMPAR-mediated signaling, ATP deficit, and facilitated depolarization, collectively shifting the network toward hypersynchronous activity. (**f**) Inhibitory synapses are suppressed because GABAergic restraint weakens, astrocyte-neuron GABA-glutamine cycling and GABA handling are disturbed, and altered chloride homeostasis reduces the inhibitory efficacy of GABA_A_ receptor signaling. Together, BBB dysfunction, glial inflammatory amplification, OS, and mitochondrial failure form positive feedback loops that destabilize neuronal homeostasis, shift excitatory/inhibitory balance toward excitation, and promote seizure activity and susceptibility. Abbreviations: AQP4, aquaporin 4; CpG DNA, cytosine–phosphate–guanine DNA; CREB, cyclic adenosine monophosphate (cAMP) response element-binding protein; DAMPs, damage-associated molecular patterns; dsRNA, double-stranded RNA; EAAT1/2, excitatory amino acid transporter 1/2; ECS, extracellular space; E/I, excitatory/inhibitory; EP2, prostaglandin E receptor 2; GAT, GABA transporter; GFAP, glial fibrillary acidic protein; GLT1, glutamate transporter 1; Gln, glutamine; Glu, glutamate; GPX4, glutathione peroxidase 4; HSPs, heat shock proteins; IL-1R1, IL-1 receptor type 1; KCC2, potassium-chloride cotransporter 2; Kir4.1, inwardly rectifying potassium channel 4.1; MCU, mitochondrial calcium uniporter; MMP-9, matrix metalloproteinase-9; mPTP, mitochondrial permeability transition pore; mtDNA, mitochondrial DNA; mtROS, mitochondrial reactive oxygen species; NKCC1, sodium-potassium-chloride cotransporter 1; nNOS, neuronal nitric oxide synthase; NOX2, NADPH oxidase 2; PAMPs, pathogen-associated molecular patterns; PKA, protein kinase A; PKC, protein kinase C; Poly(I:C), polyinosinic:polycytidylic acid; PRRs, pattern-recognition receptors; P2X7, purinergic receptor P2X7; Rac1, Ras-related C3 botulinum toxin substrate 1; RAGE, receptor for advanced glycation end products; RNS, reactive nitrogen species; S100B, S100 calcium-binding protein B; Smad2/3, SMAD family member 2/3; SOD2, superoxide dismutase 2; Src, proto-oncogene tyrosine-protein kinase Src; ssRNA, single-stranded RNA; TCA, tricarboxylic acid; TGF-βR, transforming growth factor beta receptor; TNFR1, TNF receptor 1; VDAC1, voltage-dependent anion channel 1; VGAT, vesicular gamma-aminobutyric acid transporter; VGLUT, vesicular glutamate transporter; ZO-1/2, zonula occludens 1/2. (Created in BioRender. Trofimov, A. (2026) https://BioRender.com/lfrolc1 accessed on 13 July 2026).

Experimental research and clinical evidence have established that neuroinflammatory processes contribute to both epileptogenesis and the persistence of chronic epilepsy [44,83,84,92,93,94,95]. In this section, we briefly outline the key mechanisms (Figure 1) and their clinical relevance.

#### 1.3.1. Historical and Clinical Evidence

The link between inflammation and seizures emerged from clinical observations long before the underlying mechanisms were identified. Pediatric studies reported elevated IL-6 and altered IL-1 system profiles, suggesting that systemic immune activation can lower seizure threshold [96,97]. Other inflammatory conditions, including lupus erythematosus and CNS infections, were also recognized as important causes of both acute symptomatic seizures and epilepsy [98,99,100]. Further studies extended these observations to the interictal state. Circulating IL-6 and other inflammatory markers were found to be abnormal between seizures, suggesting that chronic epilepsy carries a persistent systemic inflammatory component [83,101]. Surgical specimens from patients with drug-resistant TLE showed gliosis and chronic inflammatory glial remodeling within seizure foci [52,102], while other studies reported elevated cytokines in cerebrospinal fluid after recent seizures [103]. PET imaging studies in TLE demonstrated chronically increased glial activation ipsilateral to the seizure focus [94,95]. All these findings together progressively established a link between both infectious and sterile (non-infectious) inflammation and seizure disorders [99].

#### 1.3.2. Cellular and Molecular Mechanisms

**Interleukin-1β (IL-1β)** is among the best-characterized proconvulsant mediators. A study using a rodent model of febrile seizures demonstrated that IL-1 receptor type 1 (IL-1R1) signaling is required for fever-induced seizures. IL-1R1-deficient mice were resistant to febrile seizures, whereas exogenous IL-1β lowered seizure threshold and, at higher doses, induced seizures in wild-type but not receptor-deficient animals [104]. In another mouse study, both intracerebral administration of the IL-1 receptor antagonist (IL-1Ra) and astrocytic overexpression of IL-1Ra suppressed experimental seizures [105]. The proconvulsant mechanism of IL-1β involves direct facilitation of NMDAR function through NR2B subunit phosphorylation, thereby increasing Ca^2+^ influx. NR2B-selective antagonists, such as ifenprodil, block IL-1β-mediated seizure prolongation [106,107]. Microglia and astrocytes are the principal sources of IL-1β in the brain. During seizures and SE, these cells rapidly release IL-1β, tumor necrosis factor (TNF), and IL-6, thereby promoting hyperexcitability in a feed-forward manner [45,107].

**NLRP3 inflammasome.** The NLR family pyrin domain-containing protein 3 (NLRP3) inflammasome is a key upstream regulator of IL-1β release. This multiprotein complex (composed of NLRP3, apoptosis-associated speck-like protein containing a CARD (ASC), and caspase-1) is activated by danger signals, including extracellular ATP and ROS, both of which arise during seizures. Caspase-1 activation leads to cleavage of pro-IL-1β and pro-IL-18 into their mature forms [55,108]. NLRP3 expression is elevated in neurons and microglia in epileptic brain tissue, increased NLRP3 signaling has been described particularly in neurons and microglia, and its pharmacological or genetic inhibition reduces IL-1β and IL-18 levels, decreases seizure severity, and attenuates hippocampal neuronal injury in SE models [109,110].

**The HMGB1–TLR4 axis** is another major innate immune pathway. Seizures promote the release of the nuclear protein HMGB1 (high mobility group box 1) from neurons and glia. Extracellular HMGB1, acting as a DAMP (damage-associated molecular pattern), activates TLR4 (Toll-like receptor 4) on glial cells and neurons, driving a proconvulsant inflammatory response that includes NR2B phosphorylation and NMDAR-dependent excitability enhancement [107]. Genetic TLR4 deletion and pharmacological HMGB1 blockade both reduce seizure severity in mouse models, while the upregulation of HMGB1 and TLR4 has been confirmed in human refractory epileptic tissue [107]. Importantly, HMGB1 contains 3 key cysteines (C23, C45, C106), and their redox state determines the extracellular signaling function of HMGB1. Its disulfide form (C23-C45 bond) signals through the TLR4/MD-2 complex to induce cytokines; the fully reduced form acts primarily as a chemoattractant via the CXCL12-CXCR4 axis; the fully oxidized extracellular form is inactive [111,112,113].

**TNF (tumor necrosis factor)** is another cytokine that plays a complex, receptor-dependent role in seizure modulation [114,115]. Proconvulsant effects are linked principally to TNFR1 (tumor necrosis factor receptor 1), which promotes surface insertion of excitatory AMPARs and reduces GABA_A_R-mediated inhibition, while TNFR2 signaling is more commonly associated with homeostatic or anticonvulsive responses [114,116,117].

**IL-6** is rapidly released in the CNS and the periphery during seizures, with serum and CSF levels rising particularly after recurrent or generalized tonic–clonic seizures [118,119]. IL-6 contributes to seizure susceptibility by amplifying inflammatory cascades, inducing acute-phase responses, and affecting network plasticity [44,45]. IL-6-deficient mice exhibit altered susceptibility to several convulsant stimuli [120].

**Cyclooxygenase-2 (COX-2)** and downstream prostaglandin signaling pathways are induced rapidly after seizures and contribute to neuroinflammation, altered excitability, and BBB dysfunction [121,122,123,124,125,126]. In SE models, EP2 receptor antagonism reduces delayed mortality and brain inflammation [126]. Upstream IL-1R1 and TLR4 pathways converge on MyD88-IRAK-TRAF6, which activates NF-κB and MAPK signaling, driving the expression of COX-2, iNOS, and other effectors [45,107,127].

**Complement** components are also increasingly recognized as contributors to epileptogenesis. After seizures, reactive glial cells produce complement factors C1q and C3, which promote synaptic remodeling and microglia-mediated pruning, inducing excitation-inhibition imbalance [128,129]. In a pilocarpine-induced SE mouse model, neutralization of C1q during epileptogenesis interrupted complement-driven synaptic engulfment, preserved synaptic and neuronal integrity, and reduced interictal epileptiform activity [130].

#### 1.3.3. Microglial and Astrocytic Involvement

Microglia and astrocytes are now recognized as active contributors to epilepsy pathophysiology. In epileptic tissue and experimental models, both cell types undergo marked phenotypic and functional remodeling that influences neuronal excitability, synaptic organization, inflammatory signaling, and BBB integrity [58,60,61,62].

Microglia contribute to hyperexcitability by releasing IL-1β, TNF, and other inflammatory mediators, by interacting with neurons through purinergic and complement-related pathways, and by reshaping synaptic connectivity within the epileptogenic network [59,60,129]. Microglia may also exert protective functions, including debris clearance and limiting excessive network activity, so their net effect depends on the nature of the insult, disease stage, and specific signaling programs engaged [59,60,131,132].

Astrocytic dysfunction can increase seizure susceptibility by impairing extracellular K^+^ buffering, glutamate uptake, adenosine metabolism, metabolic support, gliotransmission, and BBB interactions [133]. In epilepsy, reactive astrocytes exhibit a combination of impaired homeostatic support and increased inflammatory signaling, leading to extracellular glutamate accumulation, disturbed ionic regulation, altered purinergic and adenosine signaling, and facilitation of leukocyte recruitment [58,133].

Importantly, microglia–astrocyte bidirectional crosstalk amplifies both cell types’ contributions, thereby further amplifying inflammatory signaling [132]. Inflammatory microglia shift toward increased aerobic glycolysis, while reactive astrocytes undergo reprogramming of glycolysis, lipid handling, and mitochondrial function [91,134,135,136,137]. These metabolic changes influence cytokine production, redox balance, and glutamate handling, and impair normal astrocyte-neuron metabolic coupling, thereby influencing seizure susceptibility.

#### 1.3.4. Blood–Brain Barrier Disruption

Blood–brain barrier (BBB) disruption contributes to epileptogenesis, and this relationship is bidirectional. Seizures and SE can impair BBB integrity, whereas BBB breakdown itself can facilitate seizures and epileptogenic remodeling [71,138,139]. During seizures, BBB permeability increases through several interacting mechanisms, including inflammatory cytokine signaling, induction of matrix metalloproteinases (MMP), vascular endothelial growth factor (VEGF)-mediated vascular changes, and degradation of the tight junctions [71,140,141,142].

Serum albumin extravasation is one of the best-characterized epileptogenic consequences of BBB disruption. Albumin entering the parenchyma through a compromised BBB activates transforming growth factor-β (TGF-β) signaling in astrocytes through activin receptor-like kinase 5 (ALK5), leading to downregulation of inwardly rectifying potassium channel Kir4.1. This impairs K^+^ buffering and leads to an increase in activity-dependent K^+^ accumulation, facilitating neuronal depolarization and NMDAR-dependent hyperexcitability [138,143]. TGF-β/ALK5 signaling also drives broader astrocytic inflammatory transformation and pro-epileptogenic gene-expression changes [143,144]. ALK5-directed inhibitors (SJN2511, SB431542) and losartan attenuate albumin-induced signaling and reduce epileptogenesis in experimental models [138,143,144].

BBB dysfunction may also contribute to pharmacoresistance. P-glycoprotein, encoded by *ABCB1*, is overexpressed in epileptogenic tissue and reduces intracerebral exposure to ASMs [145,146]. Hypoxia-inducible factor 1α (HIF-1α) provides a mechanistic link between vascular stress and *ABCB1* upregulation, connecting BBB dysfunction directly to reduced treatment responsiveness [146,147]. Thus, BBB disruption is especially relevant in DRE.

#### 1.3.5. Peripheral Immune Cell Infiltration and Systemic Inflammation

Substantial evidence indicates that peripheral immune cells enter epileptogenic tissue and contribute to seizure-related inflammation, particularly when BBB integrity is compromised. Following SE, blood-borne monocytes infiltrate the hippocampus and other affected regions, where they amplify local inflammatory signaling [148,149]. This recruitment is driven by chemokine C-C motif ligand 2 (CCL2) and its receptor CCR2. After SE, CCL2 is induced in resident myeloid cells and neurons, generating a chemotactic gradient that draws circulating CCR2^+^ monocytes into the brain [148,149]. Disrupting the CCL2–CCR2 axis reduces monocyte recruitment, lowers hippocampal IL-1β, attenuates neuronal damage, and improves post-SE outcomes [148,149,150,151].

Infiltrating T lymphocytes, including CD4+ and CD8+ populations, have been identified in brain tissue from DRE patients and experimental models, and their entry is facilitated by integrin-mediated leukocyte-endothelial adhesion [152,153]. Analysis of resected brain tissues in pediatric intractable epilepsy demonstrated that proinflammatory γδ T cells producing IL-17 and GM-CSF (granulocyte-macrophage colony-stimulating factor) were concentrated in the lesions, which correlated positively with disease severity, whereas regulatory T cells showed an inverse relationship [153].

Neutrophil recruitment also contributes to epileptogenesis, particularly in the acute phase after SE. Experimentally induced seizures increase leukocyte rolling and adhesion in cerebral vessels, and interference with leukocyte-vascular adhesion markedly reduces both acute and spontaneous seizures and BBB leakage [152].

Systemic inflammatory stimuli can also lower seizure threshold and promote epileptogenesis through peripheral-central immune crosstalk. Systemic lipopolysaccharide (LPS) reduces the threshold for seizure induction in a dose- and time-dependent manner, and this effect is blocked by nitric oxide synthase (NOS) or cyclooxygenase (COX) inhibition [154]. In rodents, LPS administration before heat-induced seizures exacerbated seizures and led to microglial activation, while neither LPS nor seizures alone induced any microglial changes [155]. These findings extend epileptogenic neuroinflammation beyond the CNS to encompass peripheral immune activation, endothelial signaling, and leukocyte trafficking [45,156,157,158].

#### 1.3.6. Syndrome-Specific Inflammatory Patterns

Although the mechanisms described above are relevant across epilepsy types, their relative weight and temporal profile vary substantially between syndromes, and the relative role neuroinflammation plays in epileptogenesis is context-dependent [55,86,107,159,160,161,162,163,164,165].

Rasmussen encephalitis represents an example of a primarily inflammatory epilepsy. It is characterized by unihemispheric inflammation, pronounced T-cell infiltration, microglial activation, and progressive neuronal loss. Seizure control ultimately requires hemispherectomy in many patients [160,163]. Autoimmune epilepsies (anti-NMDAR, anti-LGI1, and anti-CASPR2 encephalitis) show CSF pleocytosis, intrathecal immune activation, and BBB dysfunction. Seizures often respond to immunotherapy [45,162]. These disorders illustrate that inflammatory mechanisms can be sufficiently central to warrant immunological treatment as first-line management.

In TLE, studies of surgically resected tissue from refractory TLE patients, especially with hippocampal sclerosis, consistently show astrocyte activation and neuroinflammation, detecting changes in IL-1β, IL-18, HMGB1, CCL2, COX, TGF-β, NF-κB [94,107,159,166,167,168]. Post-traumatic and post-stroke epilepsies also involve microglial activation, cytokine induction, and BBB disruption during the latent period between the initial lesion and the onset of spontaneous recurrent seizures [86,161,164,169]. In these acquired epilepsies, anti-inflammatory intervention during this window could modify epileptogenic progression. Even some genetic epilepsies may acquire inflammatory components. For example, in Dravet syndrome, neuroinflammatory signaling has been implicated in disease progression beyond the primary channelopathy [55,165].

In summary, these findings collectively establish neuroinflammation as a bidirectionally reinforced component of the distal triad: cytokine signaling, glial dysfunction, and BBB disruption converge to lower the seizure threshold, supporting the conditions for chronic epilepsy. Notably, these inflammatory cascades also generate oxidative stress as a direct byproduct, connecting neuroinflammation to the second arm of the triad examined in Section 1.4.

### 1.4. Oxidative Stress and Its Association with Epilepsy

Multiple lines of experimental and clinical evidence link oxidative stress to epileptogenesis, seizure generation, and neuronal injury in chronic epilepsy [170,171,172,173]. Here, we briefly outline the key ROS sources, molecular targets, and regulatory systems (Figure 1) and their relevance to seizure threshold [170,171,172,173].

#### 1.4.1. Clinical Evidence

Although findings vary among studies, serum and cerebrospinal fluid samples from patients with epilepsy tend to show reduced total AOX status alongside elevated indices of oxidative damage, including abnormalities in glutathione-related parameters, MDA, protein carbonyls (PC), nitric oxide-related metabolites, and AOX enzyme activities [174,175,176,177,178,179,180,181,182,183,184]. In patients undergoing surgery for DRE, systemic redox biomarkers were reported as abnormal and, in some cases, normalized after surgical treatment [185]. Tissue analysis of resected epileptogenic regions identified lipid peroxidation (LPO), NADPH oxidase activation, and oxidative modification of aquaporin-4 in one study [186], and increased levels of superoxide anion, catalase, and DNA oxidation with no LPO in another [187]. Untreated patients showed higher MDA and PC levels than treated patients [188], and blood AOX parameters partially normalized after pharmacological seizure control [177].

#### 1.4.2. Sources of Reactive Oxygen Species (ROS) in Epilepsy

Reactive oxygen species generation in epilepsy arises from several enzymatic and non-enzymatic sources. NADPH oxidases, particularly NOX2, are expressed in neurons and microglia and are rapidly activated during seizure-related excitatory signaling [189]. NOX enzymes are dedicated superoxide-producing systems. In mouse models, pharmacological inhibition of NOX suppressed seizure initiation [189]. NMDAR signaling can induce superoxide production through NADPH oxidase-dependent mechanisms [190]. Mitochondria are another major source of ROS in epilepsy, particularly at complexes I and III of the electron transport chain, where electron leakage can generate superoxide [191]. Kainate-induced seizures increased mitochondrial superoxide production in the hippocampus, and this effect was attenuated in transgenic mice overexpressing mitochondrial superoxide dismutase-2 [192].

Additional enzymatic sources of ROS include COX-2, xanthine oxidase, and iNOS under inflammatory conditions [123,193]. Furthermore, NOX-derived ROS and mitochondrial ROS generation pathways reinforce one another, while inflammatory signaling further increases NOX expression and activity, thereby linking OS and neuroinflammation [172,194].

#### 1.4.3. Oxidative Damage: Molecular Targets and Consequences

Accumulation of ROS and RNS in epilepsy causes broad molecular injury affecting lipids, proteins, and nucleic acids. Lipid peroxidation of polyunsaturated membrane phospholipids generates reactive aldehydes (such as MDA and 4-HNE) that form adducts with proteins, alter membrane properties, and disrupt the normal function of receptors, transporters, and ion channels [171,186]. F2-isoprostanes, reliable markers of LPO, are elevated in experimental seizure models [195,196]. Resected tissue obtained from DRE patients showed high levels of 4-HNE protein adducts, accompanied by signs of elevated NOX2 activation [186]. Seizure-induced OS impacts mitochondrial DNA (mtDNA) more than nuclear DNA, and recurrent seizures lead to accumulation of mtDNA damage and impaired mtDNA repair capacity [197]. These forms of oxidative injury have been documented in both experimental epilepsy and human resected tissue [185,186,191,195,197]. Collectively, oxidative damage in epilepsy has long-lasting consequences: it disrupts membrane and protein function, mitochondrial DNA integrity, and may sustain neuronal loss and persistent hyperexcitability.

#### 1.4.4. Endogenous Antioxidant Defense Systems and Their Failure in Epilepsy

Enzymatic and non-enzymatic AOX systems maintain ROS within homeostatic limits under physiological conditions but become insufficient or dysregulated in epilepsy [170,173]. The superoxide dismutase (SOD) family catalyzes conversion of superoxide to hydrogen peroxide and oxygen, with three distinct compartment-specific isoforms: cytosolic Cu/Zn-SOD (SOD1), mitochondrial MnSOD (SOD2), and extracellular SOD (SOD3) [198,199]. In epilepsy models, SOD activity shows temporal and regional dysregulation, with compensatory increases early after acute seizures and depletion in chronic conditions [170,171].

Glutathione (GSH) peroxidases (GPxs) and catalase detoxify hydrogen peroxide and lipid hydroperoxides, though catalase activity is low in brain tissue [173,198]. GSH is required as a cofactor for GPx activity and must be continuously regenerated from oxidized glutathione (GSSG) by glutathione reductase using NADPH [200]. Astrocytes play a central role in maintaining neuronal GSH supply [201]. Brain GSH is depleted in epileptic patients [202], and glutathione-related redox balance is disrupted in epileptogenic tissue [198,200]. Critically, NADPH oxidase activity consumes NADPH pool required for GSH regeneration, creating metabolic competition between ROS production and antioxidant recycling, thereby favoring persistence of OS [46,194].

#### 1.4.5. The Nrf2-Keap1-ARE Pathway: Master Regulator of Antioxidant Response

Nuclear factor erythroid 2-related factor 2 (Nrf2) is a transcriptional regulator that coordinates expression of a broad set of AOX and cytoprotective genes whose promoters contain antioxidant response elements (AREs). Under normal conditions, Nrf2 remains in the cytoplasm, where Kelch-like ECH-associated protein 1 (Keap1) targets it for proteasomal degradation. Oxidative or electrophilic stress modifies reactive cysteine residues on Keap1, releasing Nrf2 to translocate to the nucleus, where it heterodimerizes with small Maf proteins and activates ARE-containing genes involved in glutathione synthesis, peroxide detoxification, thioredoxin signaling, and iron handling [203]. In the kainate rat model, pharmacological activation of Nrf2 with a Keap1 inhibitor reduced seizure frequency and severity and increased the proportion of seizure-free animals after SE [204]. Notably, combined treatment with the NADPH oxidase inhibitor and Keap1 inhibitor was more effective in reducing seizure frequency than either treatment alone [205].

#### 1.4.6. Ferroptosis: Iron-Dependent Oxidative Cell Death

Ferroptosis is a regulated form of cell death driven by iron-dependent LPO. Iron promotes ferroptosis via two routes: directly generating highly reactive oxidants through Fenton-type chemistry and through enzymatic LPO as a component of lipoxygenase enzymes [206]. The key suppressor of ferroptosis is glutathione peroxidase 4 (GPX4), which reduces lipid hydroperoxides to lipid alcohols using GSH [207,208,209]. Cystine/glutamate antiporter system x_c_^−^ (xCT/SLC7A11) provides cystine for GSH synthesis, and its inhibition triggers ferroptosis [207,210]. In parallel, the ferroptosis suppressor protein 1 (FSP1)-coenzyme Q10-NAD(P)H pathway regenerates reduced coenzyme Q10 as a lipophilic radical-trapping antioxidant [208,209].

In epilepsy models, ferroptosis is associated with GSH depletion, reduced GPX4 activity or expression, elevated 4-hydroxynonenal (4-HNE), iron accumulation, and characteristic mitochondrial ultrastructural changes [210,211,212,213]. In the kainate TLE model, inhibition of ferroptosis ameliorated cognitive impairment and neuronal injury [211]. In PTZ-kindling and pilocarpine models, the ferroptosis inhibitor ferrostatin-1 reduced seizures and restored GPX4 and GSH while decreasing 4-hydroxynonenal, MDA, iron accumulation, and *Ptgs2* expression [212]. Nrf2 regulation of GSH synthesis, iron handling, and expression of SLC7A11 and GPX4 directly influences resistance to ferroptosis [214,215], placing ferroptosis at the intersection of OS, iron metabolism, and the broader antioxidant response.

#### 1.4.7. Oxidative Modulation of Neuronal Excitability and Seizure Threshold

Oxidative stress directly modulates neuronal excitability through redox modification of ion channels, altering gating and inactivation kinetics and conductance [216]. Reduction appears to potentiate, and oxidation to inhibit, Ca^2+^ influx through NMDA receptors [217], and this redox sensitivity is mediated by three cysteine residues with subunit-specific kinetics [218], which might serve as a feedback mechanism to dampen excitability when ROS levels rise [219]. On the other hand, high-affinity glutamate transporters GLT-1 and GLAST also carry redox-sensitive sulfhydryl groups, and their oxidative modification reduces glutamate uptake, favoring extracellular accumulation and prolonged excitatory signaling [220]. Together, these effects provide a plausible mechanistic route by which oxidative imbalance can shift neuronal networks toward hyperexcitability.

OS also contributes to seizure susceptibility indirectly through BBB disruption. ROS and RNS activate matrix metalloproteinases and degrade tight junction and basal lamina components, while LPO products further compromise endothelial function [221,222]. The resulting BBB failure permits albumin extravasation into brain parenchyma, where albumin activates TGF-β signaling in astrocytes and promotes pro-epileptogenic remodeling, as discussed in the previous section [138,223]. Therefore, OS influences seizure threshold both directly, through modulation of neuronal and synaptic signaling, and indirectly, through destabilization of the vascular–neural interface.

### 1.5. Mitochondrial Dysfunction and Its Association with Epilepsy

Mitochondria serve as central integrators of neuronal energy metabolism, calcium homeostasis, and cellular stress responses, each of which is disrupted in epilepsy [49,224,225,226,227,228,229]. In this section, we outline the key mechanisms (Figure 1) and clinical evidence that establish the relevance of mitochondria to threshold management in epilepsy.

#### 1.5.1. Bioenergetic Failure and Neuronal Hyperexcitability

Under physiological conditions, at least half of neuronal ATP expenditure is devoted to restoration of ion gradients, and this demand rises further during sustained network activity [224,228]. When mitochondrial ATP production declines, Na^+^/K^+^-ATPase function becomes compromised; however, rather than producing a generalized membrane depolarization, the primary consequence appears to be failure of the synaptic vesicle cycle, with disproportionate impact on high-frequency firing inhibitory interneurons [230]. Consequently, impairment of mitochondrial function in these cells can weaken network inhibition and thereby lower seizure threshold [228,230].

In acquired epilepsy models, mitochondrial dysfunction is associated with impaired respiratory chain function, ATP depletion, and increased seizure susceptibility [191,192,231]. Conversely, the ketogenic diet’s antiseizure efficacy in the PTZ model was linked to increased mitochondrial biogenesis and improved bioenergetic function [232], suggesting that bioenergetic dysfunction is not merely a consequence of seizures but a mechanistically relevant contributor to persistent network hyperexcitability.

#### 1.5.2. Calcium Homeostasis and mPTP-Mediated Injury

Beyond energy production, mitochondria are a major site of intracellular Ca^2+^ buffering, taking up cytosolic Ca^2+^ through the mitochondrial calcium uniporter (MCU) and thereby limiting excitotoxic injury during periods of increased neuronal activity [233]. This buffering role is directly relevant to seizure-induced injury: MCU inhibition significantly attenuated neuronal death and reduced intracellular ROS following pilocarpine-induced SE [234]. Excessive mitochondrial Ca^2+^ loading, particularly during prolonged glutamatergic transmission, triggers opening of the mitochondrial permeability transition pore (mPTP), leading to collapse of ΔΨm, uncoupling of oxidative phosphorylation, ATP depletion, and release of cytochrome c [235,236,237]. In the low-Mg^2+^ seizure-like activity model, NMDAR-driven Ca^2+^ oscillations produced cyclosporine A-sensitive mitochondrial depolarization and ATP loss, and neuronal death was attenuated by pyruvate supplementation [226]. The threshold for mPTP opening is modulated by Ca^2+^ load and OS, which are perturbed in epileptic tissue [64,233,235,236], and the deletion of the cyclophilin D subunit of the mPTP complex abolishes the antiseizure effect of KD in spontaneously epileptic transgenic mice [238].

#### 1.5.3. DAMPs and Inflammasome Activation: Mitochondria-Inflammation Crosstalk

Damaged mitochondria propagate neuroinflammation through the release of damage-associated molecular patterns (DAMPs), including mitochondrial DNA, cardiolipin, and N-formyl peptides, that activate pattern-recognition receptors and trigger NLRP3 inflammasome assembly, processing pro-IL-1β and pro-IL-18 into their mature forms [49,227]. On the other hand, IL-1β potentiates NMDAR-mediated Ca^2+^ influx via Src-family-kinase-dependent phosphorylation of NR2A/2B subunits [239], amplifying the mitochondrial Ca^2+^ burden during glutamatergic activity and accelerating mPTP opening. Other inflammatory mediators, including TNF also impair mitochondrial respiratory function [240]. Thus, mitochondrial damage and inflammatory signaling form a self-amplifying loop.

#### 1.5.4. Mitophagy and Mitochondrial Biogenesis

Cells normally maintain mitochondrial health through coordinated quality control and regeneration. Damaged mitochondria are selectively cleared through the process of mitophagy via the PINK1-Parkin pathway [241], while peroxisome proliferator-activated receptor γ coactivator 1-α (PGC-1α) activates transcriptional programs leading to mitochondrial biogenesis. Hippocampal tissue from refractory TLE patients shows the accumulation of mitochondria with inadequate autophagic clearance [242]. Compensatory upregulation of PGC-1α and biogenesis machinery occurs acutely after prolonged seizures, while resveratrol enhances this response and reduces damage following SE in the kainate rat model [243]. Interventions that restore quality control show antiseizure effects: KD and caloric restriction enhance mitochondrial biogenesis and broader bioenergetic adaptations in the hippocampus [232,244]. mTOR inhibition promotes autophagy in experimental models, and its inhibitor, everolimus, improved seizure control in the majority of epileptic patients with tuberous sclerosis complex [245,246]. Together, these observations position mitochondrial quality control as a plausible target for seizure-threshold management.

#### 1.5.5. Primary Mitochondrial Epilepsies: Genetic Proof-of-Principle

The causal link between mitochondrial dysfunction and epilepsy is most directly demonstrated by primary mitochondrial diseases—genetic disorders, in which seizures are frequent and clinically prominent [247,248]. Mitochondrial encephalomyopathy with lactic acidosis and stroke-like episodes (MELAS; m.3243A>G variant in *MT-TL1* gene, which encodes mitochondrial tRNA^Leu^), myoclonic epilepsy with ragged-red fibers (MERRF; m.8344A>G variant in *MT-TK* gene, which encodes mitochondrial tRNA^Lys^), and *POLG*-related disorders, including Alpers–Huttenlocher syndrome with its refractory epilepsy and hepatic involvement, illustrate how impaired oxidative phosphorylation alone can drive seizures [247,248,249,250].

These syndromes, occupying the severe end of this spectrum, constitute proof-of-principle that mitochondrial dysfunction, even without a primary channelopathy or structural lesion, is sufficient to lower seizure threshold into the clinical range. This rationale extends to acquired epilepsies, where mitochondrial impairment is less severe but may still contribute substantially to threshold lowering [248,251].

Taken together, bioenergetic failure, disruption of Ca^2+^ homeostasis, inflammatory crosstalk, and breakdown of mitochondrial quality control converge with the neuroinflammatory and OS processes described in Section 1.3 and Section 1.4 to create a permissive metabolic environment for a chronically lowered seizure threshold. Additionally, since mitochondria are integral to synaptic transmission and synaptic plasticity through local ATP supply and Ca^2+^ buffering in the pre- and postsynaptic compartments, their impairment may contribute to epilepsy-related synaptic dysfunction and pathological plasticity that stabilizes hyperexcitable networks during epileptogenesis [252,253,254,255].

### 1.6. The Interconnectedness of Distal Mechanisms

The preceding sections have detailed neuroinflammation, OS, and mitochondrial dysfunction as distal causal factors that contribute to lowering seizure thresholds. Critically, these mechanisms do not operate in isolation. Instead, they form an interconnected triad in which each process can both drive and be amplified by the others, thereby creating self-reinforcing cycles that promote epileptogenesis and increase seizure susceptibility over time [46,79]. This interconnectedness is critical for rational therapeutic design because selective intervention at only one arm of the triad may be insufficient if the remaining two continue to sustain the same pathological state. We thus briefly summarize how these three pathophysiological domains mutually reinforce each other. This crosstalk is graphically depicted in Figure 1.

**Neuroinflammation ⇄ Oxidative Stress.** Neuroinflammation promotes OS, and OS amplifies inflammatory signaling. Activated microglia and other inflammatory cells generate reactive species through inducible enzymes such as NADPH oxidase (NOX2) and iNOS, thereby increasing production of superoxide, nitric oxide, and downstream oxidants such as peroxynitrite [79,189,256]. In the opposite direction, ROS activate redox-sensitive inflammatory pathways, including NF-κB, thereby enhancing the expression of cytokines such as IL-1β, TNF, and IL-6, as well as NOX2 and COX-2, further perpetuating inflammatory signaling [46,79]. Further coupling points include the redox-sensitive HMGB1 switch—whose disulfide and reduced isoforms differentially engage TLR4/MD-2 and CXCL12-CXCR4 signaling [111,112,257]—and the Keap1-Nrf2 pathway, through which OS induces protective counter-regulation that limits downstream inflammatory amplification [79,80,203]. Thus, inflammation increases oxidative burden, whereas OS strengthens inflammatory transcriptional and enzymatic programs. This two-way relationship is a central redox-inflammatory loop in epilepsy.

**Oxidative Stress ⇄ Mitochondrial Dysfunction.** OS damages mitochondria, whereas mitochondrial dysfunction further increases OS. Seizures increase metabolic demand, Ca^2+^ loading, and respiratory-chain stress, all of which favor mitochondrial ROS production and oxidative injury to lipids, proteins, and mitochondrial DNA [46,225,233]. In the opposite direction, oxidative damage to mitochondrial membranes, respiratory chain components, and AOX systems impairs oxidative phosphorylation, lowers ATP production, destabilizes membrane potential, and reduces the capacity of mitochondria to buffer further excitatory stress [225,226,233]. This establishes a feed-forward loop in which ROS impair mitochondrial function, and damaged mitochondria become progressively less able to restrain further ROS generation and energetic collapse.

**Neuroinflammation ⇄ Mitochondrial Dysfunction.** Mitochondrial dysfunction activates inflammatory signaling, whereas inflammatory mediators further worsen mitochondrial stress. Mitochondrial injury can result in the release of DAMPs, which activate innate immune pathways such as TLR9 signaling and the NLRP3 inflammasome [49,258,259]. These pathways promote maturation of IL-1β and IL-18 and amplify downstream inflammatory cascades. In the opposite direction, inflammatory mediators such as IL-1β increase NMDAR-mediated Ca^2+^ influx through Src-family kinase signaling, thereby driving mitochondrial Ca^2+^ overload, permeability transition, ATP depletion, and membrane injury [239].

**Distal Triad ⇄ Seizures.** Beyond their mutual reinforcement, the triad converges on the immediate machinery of seizure generation. Collectively, the distal triad promotes increased glutamatergic excitation through cytokine-dependent facilitation of NMDAR and AMPAR signaling and impaired glutamate clearance; weakened GABAergic restraint, through receptor remodeling, interneuron stress, and disturbed chloride and metabolic homeostasis; disrupted ionic homeostasis, through impaired astrocytic K^+^ buffering, Na^+^/K^+^-ATPase failure, and membrane depolarization; and reduced neuronal energy availability, through mitochondrial ATP depletion and bioenergetic insufficiency. In this way, the distal triad lowers the threshold at which immediate precipitants such as sleep deprivation, stress, fever, or other patient-specific triggers can precipitate seizures [14,41,43]. Seizures then feed back into the same distal triad by increasing Ca^2+^ loading, inflammatory signaling, ROS formation, and mitochondrial stress, thereby reinforcing the positive-feedback relationship through which seizures facilitate further seizures [46,79,233]. COX-2/PGE2/EP2 signaling provides an additional bridge, linking seizure-induced induction of inflammatory genes to sustained neuroinflammation, BBB dysfunction, and neuronal injury after SE [121,126].

This interconnectedness provides the mechanistic foundation for Part 2 of this review, which examines interventions aimed at interrupting these self-amplifying distal loops and their convergence onto proximal seizure-triggering mechanisms.

## 2. Part 2: Therapeutic Strategies for Threshold Management: Addressing Neuroinflammation, Oxidative Stress, and Mitochondrial Dysfunction

**Figure 2:**
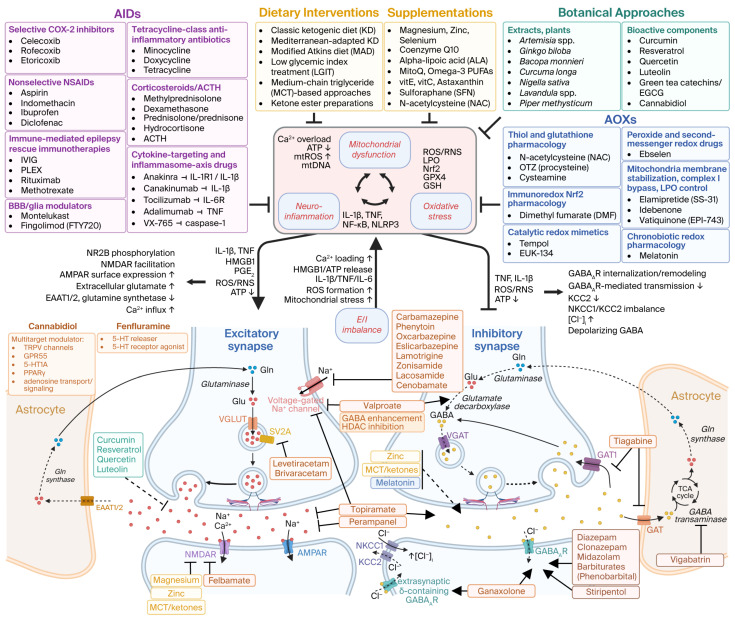
Schematic summary of the interventions reviewed in this article, arranged according to their predominant and selected secondary sites of action within epilepsy pathophysiology. ASMs are positioned mainly at excitatory and inhibitory synapses because they act predominantly on proximal seizure-generating mechanisms. AIDs, AOX pharmacological agents, and non-pharmacological approaches, including dietary interventions, supplementations, and botanical approaches, are positioned primarily around the central distal triad because they are discussed mainly as modulators of neuroinflammation, oxidative stress, mitochondrial dysfunction, and related upstream processes that lower seizure threshold. Selected non-ASM interventions with clearer direct excitability-modulating or synaptic actions are additionally placed near relevant synaptic elements. The distal triad promotes glutamatergic excitation, weakens GABAergic inhibition, and contributes to E/I imbalance, whereas seizure activity feeds back to reinforce the same distal mechanisms. All intervention classes and representative substances reviewed in the text are shown schematically; details are provided in the main text and in Tables S1 and S2. Abbreviations: 5-HT1A, serotonin receptor 1A; ACTH, adrenocorticotropic hormone; EAAT1/2, excitatory amino acid transporter 1/2; EGCG, epigallocatechin gallate; E/I, excitatory/inhibitory; EUK-134, synthetic superoxide dismutase/catalase mimetic; GAT, GABA transporter; Gln, glutamine; Glu, glutamate; GPR55, G protein-coupled receptor 55; IL-1R1, IL-1 receptor type 1; IL-6R, interleukin-6 receptor; IVIG, intravenous immunoglobulin; KCC2, K^+^-Cl^−^ cotransporter 2; MitoQ, mitoquinone; mtDNA, mitochondrial DNA; mtROS, mitochondrial reactive oxygen species; NKCC1, Na^+^-K^+^-2Cl^−^ cotransporter 1; NSAIDs, nonsteroidal anti-inflammatory drugs; OTZ, L-2-oxothiazolidine-4-carboxylate; PLEX, plasma exchange; PPARγ, peroxisome proliferator-activated receptor gamma; PUFAs, polyunsaturated fatty acids; RNS, reactive nitrogen species; TCA, tricarboxylic acid; TRPV, transient receptor potential vanilloid; VGAT, vesicular gamma-aminobutyric acid transporter; VGLUT, vesicular glutamate transporter; vitC, vitamin C; vitE, vitamin E. (Created in BioRender. Trofimov, A. (2026) https://BioRender.com/lfrolc1 accessed on 13 July 2026).

In Part 1, we reviewed the ‘vicious cycle’ in epilepsy, in which neuroinflammation, oxidative stress, and mitochondrial dysfunction interact bidirectionally with seizure activity, mutually perpetuating one another and reinforcing a chronically lowered threshold. Part 2 turns to pharmacological and non-pharmacological strategies in epilepsy—from ASMs, antioxidant and anti-inflammatory agents, to dietary and botanical interventions—tracing how they engage the distal mechanisms and with what strength of evidence. Conventional ASMs act primarily on proximal excitability, but often also influence the distal pathways [82], some in favorable, while others in unfavorable direction [260,261,262]. This secondary property may be relevant to drug resistance and long-term disease course [12,13]. Throughout this part, we distinguish mechanistic target engagement, preclinical antiseizure or antiepileptogenic evidence, preliminary clinical or translational observations, and interventions supported by stronger clinical outcome data; biomarker normalization is not interpreted as established clinical efficacy or disease modification unless supported by appropriate long-term seizure outcomes. The interventions and their mechanisms discussed in Part 2 are graphically summarized in Figure 2.

### 2.1. Pharmacological Approaches and the Distal Pathophysiological Triad

This section characterizes conventional ASMs by their differential effects on the distal triad—anti-inflammatory and AOX in some cases, pro-inflammatory or pro-oxidant in others (Section 2.1.1)—and then reviews anticonvulsant and disease-modifying effects reported for anti-inflammatory medications and AOXs in experimental and clinical epilepsy, and how these effects intersect with standard ASM therapy (Section 2.1.2 and Section 2.1.3).

#### 2.1.1. Anti-Inflammatory and Antioxidant Properties of Conventional Antiseizure Medications

For each major ASM class, we briefly summarize the principal antiseizure mechanism and then consider anti- vs. pro-inflammatory and anti- vs. pro-oxidant effects (Table S1). This is intended to distinguish drugs that may relieve the distal inflammatory and oxidative burden from those that may instead aggravate it.

##### Sodium Channel Blockers

Traditional Na^+^ channel blockers, including carbamazepine, phenytoin, oxcarbazepine, eslicarbazepine, lamotrigine, zonisamide, lacosamide, and cenobamate, limit repetitive firing by stabilizing inactivated voltage-gated Na^+^ channels. They remain first-line options in many epilepsies but differ substantially in their inflammatory and redox profiles.

- **Carbamazepine (CBZ)** illustrates this mixed profile particularly well. In peripheral inflammation models, it reduced yeast-induced hyperalgesia and edema, exudate volume, and PGE_2_-like activity [263]. In LPS-stimulated mixed glia, CBZ suppressed nitric oxide release, secretory phospholipase A_2_ activity, and PGE_2_ production with related evidence for downregulation of iNOS-linked microglial signaling [264,265]. In rat hippocampus, CBZ, but not valproate, lowered IL-1β and TNF expression under basal and LPS/seizure conditions, and astrocyte studies suggest reduced IFN-γ/TNF-induced glutamate release through A_2A_ receptor-dependent mechanisms [266,267]. In contrast, most redox findings point to a pro-oxidant pattern. Children receiving CBZ monotherapy showed reduced total AOX capacity and higher OS indices [268,269,270], while adults showed reduced paraoxonase-1 and acetylcholinesterase activities [271]. Preclinical studies further link CBZ to hepatic OS, GSH depletion, reduced SOD/GPx/CAT activities, mitochondrial dysfunction, and oxidative stress in brain microvascular endothelial cells [272,273,274,275]. Thus, CBZ combines measurable anti-inflammatory actions with a predominantly pro-oxidant, mitochondria-challenging profile in both systemic tissues and the BBB. Independent of these mechanisms, CBZ carries an HLA-A*31:01-associated risk of severe cutaneous hypersensitivity reactions [276], a pharmacogenetic safety consideration relevant to its overall risk–benefit profile.
- **Phenytoin (PHT)** and **fosphenytoin** limit repetitive firing through prolonged Na^+^ channel inactivation. Unlike CBZ, PHT has little convincing evidence for direct central anti-inflammatory activity. Clinical data instead associate chronic PHT treatment with elevated homocysteine, adverse lipid and CRP profiles, other vascular-risk markers, and case reports of severe cutaneous hypersensitivity reactions [277,278,279,280]. Its oxidative profile is clearly less favorable. In rodents, chronic PHT increased brain MDA and protein carbonyls, lowered GSH and AOX enzymes, and produced cognitive impairment responsive to AOX co-treatment [281]. In isolated rat hepatocytes, PHT induced ROS formation, GSH depletion, mitochondrial swelling, ΔΨm loss, and ATP reduction, while taurine or melatonin attenuated these effects [282]. Human studies similarly associate older enzyme-inducing ASMs, including PHT, with higher protein carbonyls or LPO and a less favorable oxidant-antioxidant balance than newer ASMs [278,283].
- **Oxcarbazepine (OXC)**, **lamotrigine (LTG)**, and **lacosamide (LCM)** appear largely neutral or mildly favorable in redox terms. OXC lowered serum nitric oxide and MDA in children with new-onset epilepsy, increased erythrocyte SOD and GPx in adults, and did not aggravate brain oxidative markers in PTZ-kindled mice [284,285,286]. LTG increased TGF-β1 without increasing TNF in stimulated glial cultures and prevented seizure-related brain MDA elevation in PTZ kindling, while preserving glutathione and antioxidant enzymes [287,288]. In chronic stress and PTZ-kindling models, LTG also improved behavioral outcomes, reduced hippocampal damage, and reinforced endogenous antioxidant defenses [289,290,291]. Clinical evidence is limited, but in one study, LTG treatment was associated with reduced serum TNF in patients with epilepsy and comorbid depressive symptoms [292]. LCM, which enhances slow Na^+^ channel inactivation, reduced hippocampal MDA, normalized catalase and SOD, maintained glutathione, and limited neuronal loss, IL-1β, TNF, and spontaneous motor seizures in pilocarpine/status epilepticus models [293,294,295]. However, this protective effect followed an inverted-U dose-response, with lower doses protective against seizures and OS but high doses aggravating both [296].
- **Zonisamide (ZNS)**, which also inhibits T-type Ca^2+^ channels, shows the most consistent protective profile within this group. It reduced microglial Nav1.6, Iba1 activation, TNF and gp91^phox^ expression, scavenged hydroxyl and nitric oxide radicals, increased astrocytic glutathione and SOD-related transcripts, and limited oxidative DNA injury and caspase-3 activation [297,298,299,300,301,302].

Overall, Na^+^ channel blockers are heterogeneous. CBZ and PHT carry the clearest oxidative or mitochondrial liabilities, whereas OXC, LTG, LCM, and ZNS appear neutral to protective, especially regarding seizure-related oxidative stress.

##### GABAergic Enhancers

GABAergic enhancers reinforce inhibitory tone through GABA_A_ receptor modulation or increased extracellular GABA. Benzodiazepines and barbiturates act as positive allosteric modulators of GABA_A_Rs, tiagabine inhibits GABA transporter (GAT)-1, vigabatrin inhibits GABA transaminase, and ganaxolone potentiates mainly extrasynaptic δ-containing GABA_A_Rs [303,304,305,306,307].

- **Benzodiazepines** such as **diazepam (DZP)**, **clonazepam**, and **midazolam** are used mainly for SE and seizure clusters, but DZP also affects immune and redox pathways. In carrageenan paw edema and pleurisy models, high-dose DZP (10 to 20 mg/kg) reduced edema, leukocyte influx, and exudate volume through an adrenal-dependent mechanism linked to peripheral benzodiazepine receptors and corticosterone [308]. With repeated dosing, this protection faded as corticosterone responses adapted [309]. In LPS-stimulated macrophages and dendritic cells, DZP lowered IL-12, TNF, IL-6, nitric oxide, and CD40 while increasing IL-10; in vivo, it improved survival after LPS shock and ameliorated experimental autoimmune encephalomyelitis [310]. It also reduced striatal LPO in acute stress and, combined with exercise, lowered MDA and GSSG while raising prefrontal GSH after LPS [311,312]. These effects are best interpreted as short-term supportive actions, not chronic anti-inflammatory therapy.
- **Barbiturates**, especially **phenobarbital (PB)**, remain effective for generalized, neonatal, and refractory status-related seizures, but their chronic non-electrophysiological profile is less favorable. PB-containing regimens are linked to reduced folate and vitamin B_6_/B_12_ status, increased homocysteine, and elevated lipid hydroperoxides or OS indices [268,313,314,315]. It is therefore best viewed as a strong seizure-suppressing GABAergic ASM with enzyme-induction-related metabolic and redox burden.
- **Tiagabine (TGB)** shows a more coherent anti-inflammatory and antioxidant profile. In BV2 cells, it limited NF-κB nuclear translocation and reduced LPS- or toxin-induced nitric oxide, IL-1β, and TNF release [316]. In PTZ-kindled mice, TGB lowered hippocampal IL-1β and IL-6, normalized BDNF/TrkB (brain-derived neurotrophic factor and its receptor) signaling, reduced microglial burden, improved anxiety-like behavior and cognition, decreased MDA, and restored SOD and GPx [317]. It also attenuated OS in primary astrocytes [318] and reduced infarct volume in ischemia models [319,320].
- **Vigabatrin (VGB)** reduced peripheral inflammatory responses and acute spinal ischemia–reperfusion injury markers [321,322]. However, prolonged or high-dose exposure produces retinal toxicity, including light-dependent retinal degeneration and S-enantiomer-driven retinal and systemic toxicity [323,324,325].
- **Valproate (Valproic Acid, VPA)** presents a more inconsistent, model-dependent profile. It can suppress TNF and IL-6 through NF-κB inhibition in vitro but did not reduce hippocampal IL-1β or TNF in an LPS/4-aminopyridine model where CBZ was effective [266,326]. Long-term treatment may promote weight gain, insulin resistance, and metabolic syndrome, supporting low-grade systemic inflammation rather than direct CNS cytokine activation [327,328]. Redox findings are mixed, but VPA is generally less pro-oxidative than classic enzyme inducers [268,329,330,331]. Serious toxicity in susceptible patients involves interference with mitochondrial β-oxidation and hepatotoxic intermediates [332,333].
- **Ganaxolone (GNX)** is a neurosteroid GABA_A_R modulator. Most mechanistic information still derives from allopregnanolone, which suppresses TLR4/NF-κB and NLRP3-linked responses and supports antioxidant and mitochondrial parameters [334,335,336,337,338]. Direct GNX studies report improved myelin debris clearance, reduced seizure burden and thalamic neuroinflammation after perinatal asphyxia, and anti-inflammatory or mitochondrial-protective effects in non-epilepsy models [339,340,341].

Thus, GABA-enhancing ASMs range from acutely anti-inflammatory agents such as DZP and TGB to agents with well-documented tissue-specific liabilities, such as VGB retinal toxicity and VPA mitochondrial hepatotoxicity.

##### Glutamate Modulation

Glutamate-modulating ASMs dampen excitatory transmission and may interrupt the excitotoxic loop that activates glia and amplifies ROS and RNS [342,343,344,345].

- **Felbamate (FBM)** acts mainly as an NR2B-preferring NMDAR antagonist with additional GABA_A_ activity. At clinically relevant concentrations, it inhibited NMDA responses, potentiated GABA responses, and reduced NMDA/glycine-evoked Ca^2+^ rises in hippocampal neurons [346,347]. In gerbil forebrain ischemia, prophylactic and delayed FBM reduced CA1 neuronal loss and infarcted area [348,349,350]. In rat traumatic brain injury (TBI), FBM was associated with lower serum IL-1β, IL-4, IL-6, and TNF, improved histology, and a more favorable TBARS/GSH/SOD profile, although the strongest effect was seen with LEV + FBM [351]. Its main clinical limitation is rare metabolite-linked aplastic anemia and hepatic failure, related to reactive atropaldehyde formation [351,352].
- **Topiramate (TPM)** combines AMPA/kainate receptor antagonism, Na^+^ channel block, GABA_A_ potentiation, and weak carbonic anhydrase inhibition. In LPS-challenged microglia, it suppressed IL-1β and IL-6, and at higher concentration TNF, while attenuating LPS-induced mitochondrial metabolic changes [353]. In vivo, TPM attenuated methylphenidate-induced amygdala TNF, IL-1β, LPO, mitochondrial GSSG, and CREB/BDNF disruption, and prevented cocaine-related NF-κB, oxidative, and cognitive changes [354,355]. It lowered PTZ-related blood LPO, improved AOX markers, normalized early hippocampal catalase and MDA after pilocarpine SE, and modulated H_2_O_2_-induced oxidative injury in PC12 cells [293,356,357]. In spinal cord injury, TPM reduced MDA, protein carbonyls, endothelial NOS, inducible NOS, and APAF-1 immunoreactivity, increased GSH, with improved recovery [358]. However, primary astrocyte cultures showed stress-related ROS, MDA, iNOS, and HSP70 responses at tested concentrations [359].
- **Perampanel (PER)**, a selective non-competitive AMPAR antagonist, has broad anti-inflammatory and AOX evidence in brain-injury models. After TBI, PER lowered IL-1β, IL-6, and TNF, reduced microglial activation, preserved the BBB/NVU (neuro-vascular unit) integrity, and improved lesion and functional outcomes [360,361]. Similar effects were reported in focal ischemia, neonatal stroke, intracerebral hemorrhage, and subarachnoid hemorrhage, with decreased cytokine expression, oxidative injury, necroptosis, pyroptosis, apoptosis, and ferroptosis-related damage, enhanced reparative markers, and attenuated necroptotic, pyroptotic, or apoptotic signaling [362,363,364,365,366,367]. A case series suggests possible utility in refractory and super-refractory status epilepticus with suspected inflammatory etiology, but this remains observational evidence [368].

Overall, glutamate-modulating ASMs appear neutral to protective regarding the distal triad, although much PER and TPM evidence comes from injury rather than epilepsy models.

##### SV2A Modulators and Other Novel Agents

Synaptic vesicle glycoprotein 2A (SV2A) modulators act upstream of classic ion channels. Levetiracetam (LEV) and brivaracetam (BRV) bind synaptic vesicle protein 2A, fine-tune Ca^2+^-dependent vesicle exocytosis, and dampen hypersynchronous vesicle release without major direct postsynaptic receptor effects.

- **Levetiracetam (LEV)** has strong epilepsy-related anti-inflammatory evidence. In post-SE and TLE models, it reduced reactive microglia and astroglia, IL-1β/IL-1R1 expression, *Fosl1*-associated inflammatory induction, and A2 astroglial markers [369,370,371,372]. Post-status treatment also limited CD11b^+^CD45^high^ leukocyte infiltration, including neutrophils and monocytes, downregulated broad cytokine and chemokine responses, and improved BBB integrity [370,373]. In glial models, LEV increased TGFβ1, reduced activated phagocytic microglia, reversed inflammation-associated astroglial dysfunction, and downregulated AP-1/*Fosl1*-dependent pro-inflammatory programs [371,374,375,376]. Clinical evidence remains limited, but in children receiving VPA, add-on LEV produced a greater reduction in serum CCL2 than VPA monotherapy, while IL-1β remained unchanged [377]. Its redox profile is generally consistent: in pilocarpine seizures and chronic TLE models, LEV reduced LPO, nitrite/nitrate, and H_2_O_2_, while preserving GSH, CAT, and SOD-related AOX capacity, although MDA, carbonyls, and 8-OHdG were not uniformly changed [378,379,380,381].
- **Brivaracetam (BRV)** is a high-affinity SV2A ligand with roughly 10-fold to 30-fold higher binding than LEV and some additional weak Na^+^ channel effects [382]. BRV reduced allodynia, dorsal horn Iba1^+^ microglia, TNF, and CD3^+^ T-cell infiltration in neuropathic pain [383], and lowered TNF, TBARS, and infarct volume while increasing IL-10, glutathione, SOD, and catalase after cerebral ischemia–reperfusion [384]. It also suppressed astroglial L-glutamate release and reduced plasma-membrane SV2A/Cx43 expression under inflammatory or fast-ripple-like stimulation [385,386]. However, therapeutically relevant BRV increased morphologically activated microglia in mixed cultures, and chronic kainate-TLE experiments reported increased microglial activation and astroglial density in several circuit regions [387,388]. Thus, BRV may be protective in acute injury models, but its inflammatory profile in established epilepsy remains unresolved.

Several syndrome-directed agents provide a translational contrast.

- **Cannabidiol (CBD)**, a component of *Cannabis sativa*, is a plant-derived modulator of transient receptor potential vanilloid (TRPV) channels, G-protein coupled receptor 55 (GPR55), 5-HT_1A_, PPARγ, and adenosine transport. It reduces seizures in Dravet and Lennox–Gastaut syndromes in randomized trials [389,390,391]. Experimentally, CBD lowers pro-inflammatory mediators, microgliosis, leukocyte infiltration, HMGB1 release, oxidative/nitrative damage, and mitochondrial dysfunction across CNS and systemic injury models [392,393,394,395,396,397,398,399,400,401]. In chronic pilocarpine epilepsy, CBD activated hippocampal autophagy and antioxidant defense while improving epilepsy-related behaviors [400,402]. Context-dependent mitochondrial impairment has been reported at higher experimental exposures, and clinically relevant interactions with clobazam and VPA require monitoring [396,403,404].
- **Stiripentol (STP)**, a GABA_A_-modulating add-on for Dravet syndrome, protected neuron-astrocyte cultures from oxygen-glucose deprivation or glutamate injury and may shift energy use through lactate dehydrogenase inhibition [405,406]. Direct anti-inflammatory evidence is limited, but post-activation microglia studies show reduced *Ptgs2* expression and cytokine/chemokine release [407]. Ischemia–reperfusion studies show reduced astrocyte damage, BBB leakage, and CA1 neuronal death [408].
- **Fenfluramine (FFA)**, a 5-HT releaser and 5-HT_2_ agonist now used as low-dose adjunctive therapy in Dravet syndrome, reduced plasma TNF and IL-1β while increasing IL-10 after LPS in rats [409]. In *Scn1a*^+/−^ mice, a Dravet syndrome model, it also reduced activated microglia, myelin debris, TUNEL^+^ cells, and mortality [410]. N-nitrosofenfluramine produces mitochondrial dysfunction and oxidative hepatotoxicity, but this is an impurity-related toxicological issue rather than evidence that therapeutic FFA is pro-oxidative [411,412].

Overall, ASMs should not be placed uniformly on either side of the distal triad. Older enzyme-inducing drugs, especially PHT, PB, and partly CBZ, have the clearest oxidative or metabolic liabilities. Several newer agents, including ZNS, TGB, LCM, TPM, LEV, PER, and CBD, reduce inflammatory or oxidative measures in experimental studies. BRV and VPA remain more context-dependent. While these secondary profiles may matter when designing future threshold-oriented strategies, the distinctions drawn here derive largely from preclinical data of varying strength, and their relevance to clinical outcomes remains to be tested prospectively in biomarker-stratified studies.

#### 2.1.2. Antiseizure and Disease-Modifying Properties of Anti-Inflammatory Drugs in Epilepsy

Recognition that neuroinflammation contributes to seizure generation and epileptogenesis has prompted investigation of anti-inflammatory drugs (AIDs) as potential anticonvulsant and disease-modifying agents. Several classes of AIDs show seizure-modulating properties in preclinical models and, increasingly, in selected clinical settings (Table S2). A recurrent theme is timing dependence: early inflammatory signaling can be adaptive, limiting excitotoxicity and supporting repair, whereas persistent neuroinflammation sustains hyperexcitability and pharmacoresistance. The AID classes reviewed below illustrate this across mechanisms and clinical contexts.

##### Selective COX-2 Inhibitors

Cyclooxygenase-2 (COX-2) selective inhibitors (coxibs) have received attention because seizures rapidly induce COX-2, increase PGE_2_ signaling, and may promote hyperexcitability, BBB dysfunction, and P-glycoprotein upregulation, with possible implications for ASM exposure and pharmacoresistance [121,123,413,414,415,416,417,418].

- **Celecoxib** reduces PGE_2_ synthesis and can indirectly influence glutamatergic transmission, BBB signaling, and drug-transport regulation [121,419,420]. In acute PTZ models, celecoxib increased seizure threshold or delayed seizure onset in several rodent studies, although effects varied with dose and model parameters [420,421]. In contrast, celecoxib did not alter seizure generation or propagation in amygdala kindling, showing that COX-2 blockade is not uniformly anticonvulsant [422]. After pilocarpine SE, celecoxib inhibited hippocampal synaptic reorganization, astrogliosis and aberrant neurogenesis, with MAPK/ERK-related signaling implicated [423]. It also reduced seizure susceptibility in LGI1-related autosomal dominant lateral temporal epilepsy, consistent with inflammatory contribution even in inherited hyperexcitability states [424].
- **Rofecoxib** increased the intravenous PTZ seizure threshold in mice, with evidence implicating adenosinergic signaling [425]. However, prophylactic, prandial rofecoxib failed to attenuate acute PTZ seizures, kindling acquisition, or an established kindled state [426]. In kainate models, rofecoxib reduced hippocampal cell death [427] but did not reliably improve later learning deficits or sustained neurodegeneration endpoints [428]. Rofecoxib itself was withdrawn from clinical use for cardiovascular toxicity, so this evidence is now of mechanistic rather than therapeutic interest.
- **Etoricoxib** reduced seizure parameters and brain OS indices in PTZ-kindled rats, but dose-sensitive regimens were associated with learning and memory impairment [429]. In WAG/Rij rats, early long-term etoricoxib reduced later spontaneous spike-wave discharges, with effects persisting after withdrawal, providing one of the clearer antiepileptogenic signals for COX-2 inhibition [430]. Clinical signals remain sparse and cautionary, because a case report described a generalized tonic–clonic seizure temporally associated with etoricoxib exposure, though a single report cannot establish causality [431].
- Experimental COX-2 inhibitors, such as **NS-398** and **SC-58236** offer the clearest evidence for this timing dependence. In kainate models, early selective inhibition aggravated seizures and neuronal death, whereas delayed inhibition prevented later PGE_2_ elevation and reduced delayed hippocampal loss [413,414]. In the lithium–pilocarpine SE, post-SE NS-398 produced hippocampal neuroprotection without changing electrographic SE duration or intensity, and diazepam co-administration enhanced this protection [432]. SC-58236 reduced PGE_2_ when given during the latent period but did not prevent epilepsy or modify later spontaneous EEG seizures [415]. Moreover, earlier or chronic-phase use could even increase mortality or seizure frequency [416].

Thus, COX-2 inhibition more often supports post-insult neuroprotection than reliable acute seizure suppression or established antiepileptogenesis. Clinical antipyretic trials in febrile seizures also show that simple fever or prostanoid suppression does not prevent recurrence [433,434].

##### Nonselective NSAIDs and Antipyretics

Nonselective NSAIDs and antipyretics act upstream of several prostanoid branches whose roles change over time after an epileptogenic insult. Early signaling can support adaptive responses, whereas later signaling may contribute to persistent pathology.

- **Aspirin** (acetylsalicylic acid) given before pilocarpine SE shortened SE latency and increased mortality, arguing against indiscriminate prophylactic use around epileptogenic insults [417]. Conversely, aspirin after lithium-pilocarpine SE reduced later spontaneous recurrent seizures, neuronal loss, mossy fiber sprouting, and aberrant neurogenesis [435]. Aspirin also modulated the effects of ASMs in combination paradigms [436,437] but showed no activity in maximal electroshock screening [438].
- **Indomethacin** reduced PTZ-induced seizure-like behavior and downregulated *il1b* and *cox2* expression in zebrafish larvae [439], while older provocation work showed antagonism of PGE_2_-related pro-seizure effects [440]. However, kainate studies again show that prostaglandin suppression can produce paradoxical effects depending on early versus late signaling [413]. Indomethacin also did not show anticonvulsant activity in maximal electroshock screening [438].
- **Ibuprofen** did not prevent febrile-seizure recurrence in randomized trials, including intermittent treatment during febrile illnesses and antipyretic strategies combined with rectal diclofenac or acetaminophen [433,434]. In chronic PTZ models, however, ibuprofen reduced seizure burden and improved inflammatory or OS markers, including EEG spike activity and prostanoid measures [441,442].
- **Diclofenac** showed anticonvulsant and biomarker effects in PTZ-kindled mice and a rotenone corneal-kindling model of DRE [443,444,445]; however, its clinical evaluation as an antipyretic for preventing recurrent febrile seizures yielded negative results [434].

Therefore, acute antipyresis or nonselective COX inhibition is not a reliable antiseizure strategy, although selected NSAID regimens can modify provoked seizures or downstream injury biology. Fenamate NSAIDs, however, may show antiseizure activity through non-COX mechanisms, including GABA_A_ receptor modulation, as reported in a human stem cell-derived neuroglial cell culture study [446].

##### Corticosteroids

Corticosteroids are the most clinically established anti-inflammatory agents used in DRE and inflammatory epilepsies, with empirical use extending back to the report by Sorel and Dusaucy-Bauloye (1958) describing adrenocorticotropic hormone (ACTH) efficacy in infantile spasms [447]. ACTH-responsive effects have also been demonstrated in experimental models of infantile spasms [448,449]. Corticosteroids suppress IL-1β, IL-6, TNF, COX-2-linked programs, microglial activation, and BBB inflammation through glucocorticoid receptor signaling. In a recent multicentre case series of steroid therapy in drug-resistant epilepsies, 62.7% of patients achieved at least 50% seizure reduction at 6 months and 6.6% achieved seizure cessation; methylprednisolone 30 mg/kg/day for 3 days was the most effective protocol in that cohort [450]. Preclinical studies also support anti-inflammatory effects of dexamethasone, which reduced hippocampal TNF and IL-1β and serum TNF in a kindling model [451] and showed seizure-modifying effects in a Theiler’s murine encephalomyelitis virus (TMEV) model, experimental febrile status epilepticus, and early-life seizure models [452,453,454].

- **High-dose IV methylprednisolone** (IVMP; steroid pulse therapy) and pulsatile dexamethasone regimens can reduce seizure frequency or interictal epileptic activity burden in subsets of pediatric and genetic DRE, although complete seizure freedom is uncommon [450,455,456,457]. In autoimmune encephalitis with new-onset seizures, first-line immunotherapy frequently includes high-dose steroids combined with IVIG or plasma exchange and is associated with high seizure-remission rates at follow-up, enabling later ASM reduction in responders [458].
- **Oral prednisolone or prednisone regimens** have produced seizure freedom or substantial seizure reduction in subsets of pediatric intractable epilepsy and epileptic encephalopathies [459,460].
- **Hydrocortisone and related schedules** have also been used in continuous spike-waves during slow sleep and EE-SWAS/ESES presentations, with syndrome-specific and variable results [450,461,462,463]. These data support the broader view that corticosteroids can be clinically meaningful when epileptic activity is closely linked to immune dysregulation, developmental encephalopathy, or sleep-activated epileptiform burden.

Overall, corticosteroids show the clearest antiseizure value when immune-driven network instability is plausible. Chronic use is limited by infection, hypertension, hyperglycemia, growth suppression, and psychiatric adverse effects.

##### Tetracycline Class Anti-Inflammatory Antibiotics

Tetracycline derivatives are of mechanistic interest because they attenuate microglial activation, IL-1β and TNF signaling, and matrix metalloproteinase-9 (MMP-9)-linked BBB and extracellular-matrix remodeling, which lie upstream of post-insult network reorganization [464,465].

In the 6 Hz model, minocycline, doxycycline, and tetracycline showed dose-dependent protection only at relatively high doses [464]. In amygdala kindling, minocycline increased latency to severe seizure stages, reduced generalized seizure duration, prevented seizure-associated changes in glutamatergic and GABAergic receptor subunits, and reduced TNF-receptor expression [466,467].

Post-insult treatment paradigms, which better approximate clinical intervention timing, provide more direct evidence. In lithium-pilocarpine epilepsy, early minocycline reduced microglial activation, IL-1β/TNF elevation, neuronal loss, and later spontaneous recurrent seizure frequency, duration, and severity [468]. In the TMEV model, acute minocycline treatment reduced acute seizure burden and improved selected long-term behavioral outcomes [469]. In electrically induced SE, however, minocycline improved some behavioral outcomes but did not prevent spontaneous seizures [470]. Clinically, evidence remains limited to isolated reports [471,472]. Doxycycline prevented seizure genesis and stabilized perineuronal nets in a kindling-related extracellular-matrix study, but aggravated spike-wave discharges in WAG/Rij rats at MMP-inhibitory doses [473,474].

Thus, tetracycline-class agents illustrate both disease-modifying potential and circuit-specific pro-seizure risk. Notably, a recent study from the Zaitsev group found that a 7-day minocycline course initiated after juvenile SE remodeled microglia toward surveillant phenotypes, restored NMDA-dependent synaptic plasticity, and reduced anxiety-like behavior without improving neuronal survival, supporting network-function modification as a disease-modifying mechanism distinct from classic neuroprotection [475].

##### Cytokine-Targeting Therapies and Inflammasome Pathway Drugs

Cytokine-targeting therapies are of mechanistic interest because IL-1β, IL-6, and TNF participate in rapid seizure-facilitating signaling, BBB dysfunction and longer glial-vascular programs implicated in epileptogenesis. Clinical translation depends on phenotype, timing, and endpoint selection, especially FIRES/NORSE vs. chronic focal DRE, and acute super-refractory SE vs. chronic epilepsy [476,477].

- **Anakinra** (recombinant IL-1 receptor antagonist) blocks IL-1α/IL-1β signaling through IL-1R1. In lithium-pilocarpine SE, post-insult anakinra reduced later seizure burden and improved behavioral outcomes [478], while combination with lamotrigine showed that effects can diverge across seizure, histological, and behavioral endpoints [479]. Independent studies also show that IL-1β/IL-1R1 blockade may be neuroprotective without preventing epilepsy, dissociating neuroprotection from disease modification [476]. Clinically, an early case report described seizure improvement with anakinra alongside cytokine normalization in FIRES [477], and an international pediatric FIRES cohort study found that earlier treatment was associated with shorter ventilation, ICU, and hospital duration [480]. Late introduction of anakinra was also associated with marked seizure reduction and subsequent seizure freedom in a patient with adult-onset Rasmussen’s encephalitis [481]. Chronic-phase reports suggest seizure reduction in selected patients, but complete seizure freedom is uncommon and attribution to anakinra specifically remains difficult in these uncontrolled settings [482,483,484,485].
- **Canakinumab** (anti-IL-1β monoclonal antibody) selectively neutralizes IL-1β; epilepsy evidence remains limited to a single case report of a patient with persistent elevation of inflammatory markers without clear autoimmune or genetic causes who responded to sequential anakinra and canakinumab [486].
- **Tocilizumab** (anti-IL-6 receptor monoclonal antibody) blocks IL-6R signaling. In WAG/Rij rats, IL-6R blockade reduced absence-seizure activity, with suppressive effects that persisted after treatment withdrawal [487]. In adult NORSE, tocilizumab terminated SE in most patients in a small, uncontrolled case series [488], and FIRES reports suggest benefit in some anakinra-refractory cases [483,489].
- **Anti-TNF therapies**, including **adalimumab**, are complicated by the receptor-specific and context-dependent biology of TNF. In Rasmussen encephalitis, open-label adalimumab reduced seizures or slowed progression in some patients in a small, uncontrolled case series [490].
- **Inflammasome and caspase-1 inhibitors**, exemplified by **VX-765**, reduce IL-1β maturation. In mice, VX-765 reduced acute seizures and dose-dependently suppressed drug-resistant chronic epileptic activity [491]; however, the evidence base remains limited, and reduced inflammation should not be equated with antiepileptogenesis unless spontaneous seizures are reduced [476].

##### Broad Immunotherapies and Repurposed Anti-Inflammatory Drugs

Broad immunotherapies target seizures with an identifiable immune driver, such as autoimmune encephalitis, NORSE/FIRES, Rasmussen encephalitis, or selected epileptic encephalopathies.

- **Intravenous immunoglobulin** (IVIG) modulates Fc receptors, complement, and cytokines. In autoimmune encephalitis it often forms part of first-line therapy with steroids and sometimes plasma exchange, while rituximab is used as second-line escalation [458]. In refractory childhood epilepsies, older controlled and open studies reported seizure reduction in subsets, but responses were heterogeneous and likely enriched in immune-suspected phenotypes [492,493,494,495].
- **Plasma exchange** (PLEX/TPE) removes circulating antibodies and inflammatory mediators; despite the lack of concrete evidence of its efficacy, international expert consensus includes it among first-line immunotherapies for NORSE/FIRES [496].
- **B-cell depletion with rituximab** (anti-CD20) can produce additional seizure remission in autoimmune encephalitis after first-line treatment failure and may reduce seizure burden in Rasmussen encephalitis, but evidence remains observational [458,497,498,499].
- **Methotrexate** is used for refractory immunotherapy of severe autoimmune CNS disease; seizures may improve as part of syndrome control, but methotrexate itself can cause neurotoxicity and seizures, particularly at high-dose or intrathecal exposure [499,500,501,502,503].
- Among repurposed small molecules, **montelukast** (CysLT_1_ antagonist) blocks cysteinyl-leukotriene signaling; it suppressed kindled and pilocarpine-induced seizures [504] and reduced PTZ-related seizure endpoints while improving oxidative, inflammatory, and BBB-related markers in several studies [505,506,507,508]. **Fingolimod** (FTY720) is an immunomodulator with central glial effects; across models, it reduced post-insult neuroinflammation, seizure burden, and seizure-associated P-glycoprotein upregulation, which may reduce pharmacoresistance via improved ASM delivery [509,510,511,512,513,514]. **Colchicine** is relevant mainly in familial Mediterranean fever, where seizure improvement is most plausibly mediated by suppression of systemic inflammatory triggers rather than intrinsic antiseizure activity; its narrow therapeutic index argues against general epilepsy adjunct use [515,516,517,518,519].

##### Acute vs. Chronic Inflammation: Therapeutic Implications

Across AID classes, the central distinction is temporal and etiological. Acute inflammation after an epileptogenic insult is not uniformly pathological, because it includes debris clearance, repair signaling, and mixed prostanoid outputs. Indiscriminate early suppression can therefore be neutral or harmful [413,415,416,433,434].

In established epilepsy, chronic neuroinflammation is more consistently maladaptive, with persistent glial activation, cytokine elevation, BBB dysfunction, and pharmacoresistance-relevant injury biology. Anti-inflammatory interventions are most justified in immune-driven phenotypes mentioned above [458,477,480,483,488,497].

Finally, reductions in cytokines, gliosis, or neuronal loss are biologically meaningful but do not constitute disease modification without long-term spontaneous-seizure outcomes on appropriate EEG follow-up [470,476].

#### 2.1.3. Antioxidant and Mitochondria-Active Pharmacology with Anticonvulsant and Disease-Modifying Potential in Epilepsy

As discussed in Section 1.4, OS in epilepsy is both a consequence of intense neuronal firing and an upstream amplifier of network instability: redox-sensitive modulation of ion channels, neurotransmitter handling, neuroimmune signaling, and mitochondrial function can lower seizure threshold and sustain postictal injury [171,520,521]. Antioxidant (AOX) pharmacology therefore serves here as a mechanistic probe of the distal inflammatory-redox-bioenergetic coupling. We restrict coverage to defined drug-class AOX or mitochondria-active agents, emphasizing glutathione buffering, lipid peroxidation (LPO), Nrf2 signaling, and mitochondrial redox homeostasis.

##### Thiol and Glutathione Pharmacology: N-Acetylcysteine

**N-acetylcysteine** (NAC) is a thiol donor and cysteine prodrug that supports glutathione (GSH) synthesis and also reshapes extracellular glutamate tone through the cystine-glutamate exchanger system x_c_^−^ (xCT/SLC7A11), thereby linking redox buffering to excitatory transmission and glial-neuronal coupling [200,522]. NAC is a prescription medicine for established antidotal and mucolytic indications, while also marketed as a supplement in some settings [523,524]. Its use in the supplement context is addressed in Section 2.2.2, and here we focus on its anticonvulsant pharmacology as a defined pharmacological agent.

In experimental epilepsy models, NAC can replenish neuronal and astroglial GSH, reduce LPO, and constrain redox-sensitive inflammatory amplification, including DAMP-linked immunoredox cascades activated during epileptogenesis [200,520,525]. However, exchange via system x_c_^−^ may also alter extracellular glutamate and metabotropic signaling, making the net effect on excitability dose-, timing-, and model-dependent [526].

In acute PTZ seizures, NAC at 300 to 600 mg/kg i.p. delayed seizure onset, reduced behavioral severity and EEG spike measures, lowered malondialdehyde (MDA), and increased SOD activity [527]. In a rat KA-TLE model, oral NAC at 100 mg/kg delayed seizure activity, increased survival of hippocampal neurons, improved spatial memory, and modulated mTOR-related signaling [528]. A related study reported improved hippocampal mitochondrial dynamics and membrane potential with mTOR downregulation, supporting mitochondrial target engagement in an epilepsy-relevant context [529]. In a rat model of acquired epilepsy induced by electrical SE, a two-week post-insult regimen consisting of NAC plus sulforaphane reduced OS during epileptogenesis, delayed the first spontaneous seizure, prevented seizure progression between 2 and 5 months, reduced late seizure frequency, rescued cognition, and suppressed disulfide HMGB1 generation [525].

Clinical data in epilepsy patients remain limited. High-dose NAC improved myoclonus and neurophysiological measures in a small sibling cohort with Unverricht-Lundborg disease [530]. A separate report described increased serum GSH paralleled by seizure improvement, without benefit for myoclonus or ataxia, in one genetically confirmed patient, whereas three other clinically diagnosed patients showed variable response and notable side effects [531]. NAC is nonetheless translationally attractive because of its drug-grade availability and established general safety profile, but evidence for epilepsy-specific efficacy remains insufficient outside research settings.

Its main limitation is context dependence: benefit is clearest in post-SE and acquired TLE models, whereas chronic NAC aggravated spike-wave discharges in WAG/Rij absence epilepsy despite improving depressive-like and cognitive comorbidities [525,526,528]. Related thiol donors, including L-2-oxothiazolidine-4-carboxylate, increase cysteine or GSH pools through the same mechanism, but direct antiseizure evidence is limited [532,533]. Thus, the thiol-buffering approach is mechanistically coherent, but NAC remains the only agent in this subgroup with meaningful epilepsy-specific validation.

##### Immunoredox Nrf2 Pharmacology: Dimethyl Fumarate

**Dimethyl fumarate** (DMF) is an approved immunomodulatory drug in multiple sclerosis and inflammatory dermatology. Its mechanism lies at the interface of immune regulation and redox control [534,535,536].

DMF activates the KEAP1-Nrf2 axis through electrophilic modification of KEAP1 cysteines and related control points, inducing HO-1, NQO1, and other cytoprotective programs while suppressing NF-κB-linked inflammatory transcription [536,537]. Monomethyl fumarate also activates hydroxycarboxylic acid receptor 2 (HCAR2/HCA2), providing an additional route for innate immune modulation [538]. Structural analyses support multiple DMF binding modes at the KEAP1 Kelch domain, consistent with combined covalent and noncovalent Nrf2 regulation [539].

In PTZ kindling, DMF reduced kindling-related pathology, improved hippocampal neuronal outcomes, activated Nrf2-linked AOX responses, and shifted inflammatory and apoptotic signaling toward a less injurious state [540,541,542]. In a kainate-induced SE model of acquired TLE, post-SE DMF increased Nrf2 activity, reduced hippocampal cell loss, decreased spontaneous seizure burden, and improved behavioral deficits; seizure reduction persisted after drug elimination, supporting disease-modifying potential [543]. A complementary kainate study identified DMF as an inhibitor of gasdermin D-mediated pyroptosis and reported fewer spontaneous recurrent seizures, lower IL-1β and IL-18, and fewer clasmatodendritic astrocytes [544].

Thus, current evidence positions DMF as a disease-modifying, anti-inflammatory-redox adjunct for post-insult or inflammatory-redox epilepsy phenotypes, rather than a high-potency acute ASM or a general add-on for drug-resistant epilepsy. Its translational constraints are lymphopenia, infection-risk monitoring, and the need for carefully selected populations [534,535].

##### Catalytic Redox Mimetics: Tempol and Mn-Catalytic Complexes

**Tempol** (4-hydroxy-TEMPO) is a membrane-permeable nitroxide with redox-cycling catalytic behavior and SOD-mimetic properties [545]. It is useful in epilepsy as a mechanistic probe because it targets superoxide-linked radical chemistry without direct interaction with classical seizure receptors or ion channels [63,520].

In acute picrotoxin seizures, tempol reduced the total number and duration of seizures, with stronger effects at 150 mg/kg [546,547]. Seven-day peripheral LPS exposure increased hippocampal OS and kainate susceptibility, while intracerebroventricular tempol reduced oxidative-damage indices and ameliorated the LPS-associated increase in seizure susceptibility [548]. Tempol’s reversal of LPS-associated seizure susceptibility indicates that inflammation-induced OS causally contributes to threshold lowering, rather than being merely a downstream consequence of seizures.

**Mn-based catalytic AOXs**, including salen-manganese EUK compounds, combine SOD- and catalase-like activities. In systemic KA seizures, EUK-134 markedly reduced OS indices and attenuated limbic neuronal damage, although seizure latency and duration changed little [549]. Catalytic redox correction can therefore reduce seizure-associated tissue injury even when acute seizure expression remains largely unchanged [63,549].

No formal epilepsy trials exist for tempol or EUK-134; both currently serve as target-validation tools rather than clinical candidates. The class also illustrates a translational distinction: seizure suppression, seizure-threshold elevation, and neuroprotection after seizures are related but separable endpoints.

##### Peroxide and Second-Messenger Redox Drugs: Ebselen

**Ebselen** is a glutathione peroxidase (GPx)-mimetic organoselenium compound that reduces hydroperoxides and RNS under excitotoxic load [550,551]. Ebselen also inhibits inositol monophosphatase (IMPase) in vitro, and lowers anterior cingulate myo-inositol in humans, indicating central target engagement within a lithium-mimetic pathway [552,553,554]. A 7T magnetic resonance spectroscopy study also showed reductions in glutamate, glutamine, and Glx, linking phosphoinositide and glutamate-related metabolic tone [553,554].

Preclinical epilepsy-model evidence for ebselen remains limited. In a mouse pilocarpine model designed to probe lithium-like seizure liability, ebselen did not potentiate seizures and instead reduced pilocarpine-driven tremor and *c-fos* activation [555]. This supports a net protective profile in a cholinergic excitotoxic context.

Short-course oral ebselen showed tolerability and efficacy signals in noise-induced hearing loss as well as acceptable tolerability in early-phase stroke and mood-disorder trials [556,557,558]. However, epilepsy-specific controlled trials are absent, and its multi-target profile complicates mechanistic attribution in ASM polytherapy. Its most realistic epilepsy use is short-course post-SE or post-injury testing with biochemical endpoints, rather than chronic unselected adjunctive treatment.

##### Mitochondria-Active Quinones and Membrane-Stabilizing Agents: Elamipretide, Idebenone, Vatiquinone

**Elamipretide** (SS-31) is a mitochondria-targeted tetrapeptide that binds cardiolipin and stabilizes inner mitochondrial membrane bioenergetics [559,560]. This pharmacology is relevant to seizure networks, where mitochondrial depolarization, ROS amplification, ATP failure, and interneuron vulnerability can sustain excitatory gain [520].

SS-31 attenuated ferroptosis in the hippocampus of epileptic rats through p38 MAPK-related signaling, with reduced LPO, preservation of GPX4, and normalization of Nrf2-related AOX responses [561].

Human epilepsy trials are absent. Clinical development in primary mitochondrial myopathy showed pharmacokinetic feasibility but mixed efficacy, including failure to meet primary endpoints in MMPOWER-3 [562,563]. Thus, SS-31 is best suited for biomarker-defined mitochondrial epilepsy contexts, not as a general antiseizure intervention. In the threshold-management framework, it represents a mitochondrial membrane-stabilizing strategy that may be most informative when mitochondrial injury can be documented before or during treatment.

**Idebenone** is a short-chain benzoquinone and synthetic coenzyme Q analog. It may support electron transfer downstream of dysfunctional complex I through NQO1-dependent reduction, thereby improving ATP generation under conditions of mitochondrial stress, while secondarily attenuating oxidative injury [564,565].

In pilocarpine-treated rats, idebenone pretreatment at 100 to 200 mg/kg i.p. for 3 days prolonged seizure latency, reduced seizure occurrence, improved survival, and attenuated hippocampal oxidative damage, including LPO, nitrate/nitrite accumulation, DNA fragmentation, glutathione depletion, and loss of Na^+^/K^+^-ATPase activity [566]. In a rotenone corneal-kindling model of mitochondrial DRE, idebenone at 10 to 40 mg/kg i.p. for 10 days reversed ASM refractoriness while increasing ATP, NQO1, and GSH and reducing TBARS [565].

No epilepsy-directed clinical trials exist, but idebenone has a clinical safety literature in mitochondrial and neurodegenerative disorders [564,567]. Its low oral bioavailability and reliance on NQO1-linked reduction constrain dose translation and patient selection.

**Vatiquinone (EPI-743)** is a redox-active quinone analog developed for mitochondrial diseases, with emphasis on lipid-peroxidation- and ferroptosis-related injury.

In patient-derived cellular systems relevant to mitochondrial epilepsy phenotypes, it prevented ferroptosis induced by GPX4 inhibition and GSH depletion and reduced LPO [568]. In vivo studies, however, found that strong in vitro anti-ferroptotic activity did not translate into survival or disease-progression benefit in the *Ndufs4*^−/−^ (Leigh syndrome) and GPX4-deficient mouse models, although vatiquinone may have reduced seizure risk in the *Ndufs4*^−/−^ model [569].

Early mitochondrial-disease reports suggested tolerability and open-label stabilization or improvement, while Friedreich ataxia testing provided additional chronic neurogenetic safety context [570,571,572,573]. A subsequent double-blind, placebo-controlled trial in mitochondrial disease-associated refractory epilepsy (MIT-E; NCT04378075; *n* = 68) did not meet its primary endpoint of reduced motor seizures, though topline results reported by press release indicated a treatment effect on seizure frequency overall and benefit on secondary endpoints, including SE and disease-related hospitalizations, in the largest subgroup, Leigh syndrome [574]. At present, seizure efficacy remains uncertain and likely requires biomarker-guided subgrouping.

##### Chronobiotic Redox Pharmacology: Melatonin

**Melatonin** is a neurohormone with radical-scavenging capacity, inducible AOX regulation, and strong circadian and sleep effects, all of which map onto seizure threshold through sleep disruption, inflammatory stress, and redox imbalance [575].

Mechanistically, melatonin signals through MT1/MT2 receptors and also engages receptor-independent AOX chemistry, while experimental studies show attenuation of ER stress and mitochondrial disturbance, kainate-induced hippocampal neurodegeneration and oxidative stress, microglial activation, and AhR–Nrf2-dependent neuroinflammatory signaling [575,576,577,578]. Post-SE melatonin treatment in kainate models also delayed or attenuated spontaneous seizure activity and reduced neuronal damage, although it did not consistently prevent epileptogenesis or associated behavioral and circadian disturbances [579,580].

Clinical epilepsy data are modest but informative: several trials measured sleep or AOX biomarkers rather than seizure frequency alone. In children with epilepsy receiving valproate monotherapy, add-on melatonin improved sleep behavior [581], quality of life [582], and AOX-enzyme activity in randomized double-blind placebo-controlled studies [583]. In children receiving carbamazepine, add-on melatonin increased glutathione reductase activity without significantly altering carbamazepine or carbamazepine-epoxide levels [584]. A small pilot study in patients with intractable epilepsy also reported that adjunctive melatonin reduced diurnal seizure frequency [585]. A crossover study with sustained-release melatonin reduced sleep latency and wake after sleep onset without clear worsening of spike density or seizure frequency [586]. In adults with idiopathic generalized tonic–clonic seizures on valproate, add-on melatonin reduced seizure-severity scores and improved sleep quality, but seizure frequency and EEG abnormalities were not clearly changed [587].

A Cochrane review concluded that evidence was insufficient for firm antiseizure-efficacy claims [588], while a 2024 meta-analysis found improved sleep latency and reduced seizure severity, but still emphasized small trials and heterogeneous endpoints [589]. A randomized trial in infantile epileptic spasms syndrome evaluated melatonin as add-on to ACTH plus magnesium sulfate and provided additional pediatric controlled data, although it addresses a specific syndrome and treatment context [590]. Therefore, melatonin functions as a sleep-redox threshold stabilizer with possible seizure-severity benefit, rather than as a direct antiseizure agent.

##### Concluding Remarks

Despite their mechanistic diversity, AOX agents act through integrated immunoredox and bioenergetic nodes that couple directly to network excitability. DMF exemplifies KEAP1-Nrf2-driven immune-redox regulation [541,543], NAC reinforces thiol buffering while modifying glutamate handling [525,526], and tempol probes whether catalytic superoxide control can reverse inflammation-sensitized seizure susceptibility [548]. Ebselen connects peroxide control with measurable CNS metabolic engagement in humans [553], while elamipretide, idebenone, and vatiquinone shift the focus toward mitochondrial membrane stability, complex-I bypass, and LPO/ferroptosis biology [561,565,568,569]. Melatonin occupies a pragmatic adjunct niche, with reproducible sleep benefits and variable seizure endpoints [588,589].

Overall, AOX drugs may stabilize seizure threshold by reducing LPO and inflammatory amplification, preserving mitochondrial bioenergetics, and shifting glial-neuronal signaling toward a less excitable set point. This effect may be most visible during latent epileptogenesis or in patients with demonstrable redox-bioenergetic vulnerability. However, most evidence remains preclinical or syndrome-restricted, and clinical development should use biomarker-stratified designs rather than broad unselected DRE populations and should include safety endpoints relevant to ASM polytherapy.

#### 2.1.4. Concluding Perspective on Pharmacological Threshold Management

The pharmacological evidence reviewed in Section 2.1 shows that epilepsy-directed drugs cannot be understood solely through their immediate effects on ion channels, neurotransmitter receptors, or synaptic release. Conventional ASMs differ substantially in how they influence neuroinflammation, OS, and mitochondrial function, and these differences may be relevant for long-term seizure control, tissue vulnerability, and pharmacoresistance. In parallel, several anti-inflammatory, immunomodulatory, AOX, and mitochondria-active agents have demonstrated antiseizure, neuroprotective, or disease-modifying properties in selected models and clinical contexts, particularly when treatment is aligned with a defined inflammatory or metabolic mechanism. At the same time, the available evidence also makes clear that no single pharmacological axis is sufficient across all epilepsies. Effects are strongly dependent on timing, syndrome, model, and endpoint, and reduction in inflammatory or oxidative markers cannot be equated automatically with prevention of spontaneous recurrent seizures. Therefore, the central conclusion of this section is not that threshold management can be reduced to one additional drug class, but that seizure threshold is shaped by a broader distal pathophysiological landscape than conventional ASM pharmacology usually captures. This provides the rationale for moving from narrow, single-mechanism distal targeting toward an integrated threshold-management approach that combines proximal ASM therapy with pharmacological—and non-pharmacological—strategies engaging the inflammatory, redox, mitochondrial, metabolic, and glial mechanisms.

### 2.2. Dietary Approaches in Epilepsy

In the following section, we summarize preclinical and clinical evidence for the efficacy of nutritional approaches as add-on treatments in epilepsy, focusing on mechanistic links to inflammatory, redox, and mitochondrial pathways.

#### 2.2.1. Ketogenic Approaches: Ketone Bodies as Metabolic Modulators

The ketogenic diet (KD), a high-fat, very-low-carbohydrate regimen mimicking key metabolic features of fasting, has been in continuous clinical use since the 1920s and remains the most extensively studied dietary therapy for DRE [591,592,593,594,595]. A Cochrane systematic synthesis of controlled KD trials in pediatric DRE found that KD outperformed usual care in terms of seizure freedom and ≥50% seizure reduction (risk ratios 3.16 and 5.80, respectively), though all evidence was rated low to very low certainty due to small study populations and lack of blinding [596]. Proposed anticonvulsant mechanisms of KD, extensively studied and reviewed over the years [244,260,597,598,599,600,601], broadly converge on the distal pathophysiological triad detailed in Part 1. Because of its well-established clinical use, with consistent evidence of efficacy and ample evidence across all three triad components, we review it here in detail as the paradigm case for metabolic threshold modulation.

**Anti-inflammatory effects.** The primary ketone body β-hydroxybutyrate (BHB) directly inhibits the NLRP3 inflammasome by reducing ASC oligomerization and speck formation through mechanisms independent of general starvation-induced molecular cascades [599]. BHB also acts as a ligand for the HCA2 (GPR109A) receptor on microglia and myeloid cells, biasing innate immune signaling toward a less pro-inflammatory phenotype with reduced NF-κB activity and attenuated oxidative-inflammatory coupling [602,603]. Notably, the neuroprotective effect of KD was lost in *Hca2*^−/−^ mice [602].

**Antioxidant effects.** Ketone bodies activate the Nrf2 pathway, upregulating antioxidant enzymes including SOD, catalase, and glutathione peroxidases, and KD induces systemic Nrf2 activation in rats [604]. In a rat TLE model, KD exposure was reported to activate the Nrf2/HO-1/GPX4 cascade and suppress ferroptosis-linked oxidative injury [605]. Furthermore, BHB functions as an endogenous class I HDAC inhibitor, increasing histone acetylation and enhancing transcription of FOXO3a-regulated antioxidant genes, sustaining AOX protection beyond the immediate presence of ketone bodies [598].

**Mitochondrial biogenesis and function.** KD has been shown to increase mitochondrial number and bioenergetic capacity in hippocampal tissue of KD-fed animals [232]. In spontaneously epileptic *Kcna1*^−/−^ mice, both KD and ketone bodies reduced spontaneous recurrent seizures and raised the threshold for mitochondrial permeability transition pore (mPTP) opening in mitochondrial preparations from KD-fed animals [238]. In the pilocarpine model, KD reduced spontaneous seizures, prevented hippocampal neuronal injury and apoptosis, and rescued seizure-induced loss of membrane potential [606].

**Metabolic substrate provision.** Ketone bodies can serve as an alternative mitochondrial fuel that can partially bypass the impaired glucose utilization documented in epileptic tissue [607,608], with BHB and acetoacetate entering the brain via monocarboxylate transporters and feeding directly into the tricarboxylic acid (TCA) cycle [609]. Notably, MCT1 expression on hippocampal microvessels is reduced in drug-resistant mesial TLE with hippocampal sclerosis, suggesting that ketone transport capacity may itself be perturbed in a subset of patients and could shape individual responsiveness to ketogenic therapy [610]. FDG-PET studies in humans on KD indicate regionally variable reductions in brain glucose metabolism, consistent with therapeutic effects deriving from both the presence of ketones as alternative substrates and the relative reduction in glucose flux [611].

**Clinical evidence.** Clinical evidence generally supports the efficacy of add-on ketogenic treatments. Seizure freedom rates up to 55% and responder rates up to 85% have been reported across clinical trials and systematic syntheses [596]. A randomized controlled trial demonstrated that 38% of children with DRE assigned to 4:1 KD achieved greater than 50% seizure reduction at 3 months vs. 6% in the control group (*p* < 0.0001) [612]. The classic 4:1 KD restrictiveness makes it difficult to adhere to. While less restrictive variants, such as modified Atkins diet (MAD) and low glycemic index therapy (LGIT), also showed efficacy and improved feasibility, they did not meet the prespecified noninferiority criterion vs. classic KD in a randomized trial in pediatric DRE (median seizure reduction at 24 weeks: 66% with classic KD, 45% with MAD, 54% with LGIT) [613]. A trial comparing 4:1 vs. 3:1 ketogenic ratios reported seizure-free rates of 55% vs. 30.5% at 3 months, with better tolerability at the lower ratio [614]. In children with refractory epilepsy, 52% assigned to MAD group achieved at least 50% seizure reduction at 3 months vs. 11.5% of controls [615]. Adult evidence is more heterogeneous. A controlled trial of MAD add-on treatment reported 35.3% responders at 2 months [616]. However, in general, adult cohort and trial data report wide response ranges with high individual variability, and long-term adherence is a practical constraint [617,618,619].

The use of medium-chain triglycerides (MCTs; triglycerides of caprylic (C8) and capric (C10) acids) in ketogenic formulations leverages their capacity for hepatic ketogenesis without significantly reducing carbohydrate intake. MCT-based treatments offer anticonvulsant effects in pediatric populations but are frequently limited by gastrointestinal adverse effects [620,621,622,623]. Similarly, ketone ester preparations can raise circulating BHB without dietary restructuring and have shown neuroprotective and bioenergetic effects in non-epilepsy experimental models as well as in memory-impaired adults [624,625,626,627]. To date, 2 trials are currently recruiting participants to assess the efficacy of add-on exogenous ketone esters for pediatric DRE and refractory SE (NCT05670847, NCT05674552). Absolute contraindications include primary carnitine deficiency, carnitine palmitoyltransferase I and II deficiency, other β-oxidation defects, and pyruvate carboxylase deficiency [628,629,630,631,632,633,634].

Despite heterogeneity of response across trials, individual variability, and apparently better outcomes in pediatric than adult populations, add-on ketogenic treatment in DRE is a well-established multifactorial intervention that targets both neuronal excitability and the underlying neuroinflammatory, redox, and bioenergetic mechanisms.

#### 2.2.2. Dietary Supplementation and Over-the-Counter (OTC) Drugs

Beyond dietary regimens, several dietary supplements and selected OTC agents have demonstrated anticonvulsant or seizure-threshold-modifying activity in preclinical studies and a growing number of clinical signals. Here we review those that have accumulated most evidence, focusing on plausible links to neuroinflammation, OS, and mitochondrial bioenergetics [171,200,520].

##### Mineral Cofactors of Excitability and Antioxidant Enzymes

**Magnesium.** As a voltage-dependent blocker of the NMDA receptors [635,636] and a cofactor for many mitochondrial enzymes [637], magnesium reduces glutamatergic excitotoxicity and the associated calcium overload that can drive mitochondrial dysfunction and OS. Intravenous magnesium sulfate is used as first-line therapy for eclamptic seizures, and a systematic review revealed a trend towards improved seizure control in non-eclamptic RSE, with 50% seizure recurrence upon magnesium withdrawal [638]. Several studies reported lower plasma magnesium in adults and children with epilepsy [639,640]. A retrospective chart review of 22 DRE cases concluded that oral magnesium supplementation reduced seizure days [641], while epidemiological analyses linked higher dietary magnesium intake to lower epilepsy prevalence, with attenuated inflammation estimated to account for half of this effect [642,643]. Given that low magnesium intake is common in Western diets, ensuring adequate intake has a favorable risk-benefit profile as an adjunct therapy in DRE, although dose, formulation, and gastrointestinal tolerance should guide supplementation decisions [644,645].

**Zinc.** Zinc modulates both GABAergic and glutamatergic transmission. At physiological concentrations, it stimulates GABA synthesis by enhancing pyridoxal phosphate formation, thereby increasing GAD activity [646] and inhibits NMDA receptors through both voltage-dependent and voltage-independent mechanisms [647,648]. As a cofactor for copper-zinc SOD1, zinc is also involved in redox homeostasis [649]. Lower circulating zinc levels have been reported across various epilepsy cohorts, with cross-sectional data suggesting a greater deficit in DRE [650,651,652]. Importantly, zinc is an acute-phase reactant. Infection and inflammation can lower circulating zinc concentrations, making cross-sectional comparisons challenging and contributing to within-subject variability in febrile illnesses [653,654,655]. A randomized, double-blind, placebo-controlled trial in children with idiopathic intractable epilepsy found that 6 months of oral zinc supplementation at 1 mg/kg/day improved seizure control vs. placebo [656]. Given zinc’s toxicity at high doses and interactions with inflammatory pathways, high-dose supplementation without monitoring zinc status is discouraged [652,656].

**Selenium.** Selenium is a cofactor for glutathione peroxidase (GPx) and thioredoxin reductase. Lower serum selenium levels have been reported in epilepsy cohorts relative to controls, further reduced in patients receiving valproate monotherapy [657,658]. In PTZ-based animal models, selenium supplementation reduced seizure susceptibility and lipid peroxidation [659,660]. While no controlled human trials of selenium as an antiseizure adjunct therapy have been conducted to date, existing evidence and expert opinion support monitoring selenium status and correcting deficiency, particularly in patients on valproate [658,661].

##### Mitochondria and Membrane Redox Support

**Coenzyme Q10.** CoQ10 functions both as an electron carrier in the mitochondrial respiratory chain and a lipophilic antioxidant protecting membranes from lipid peroxidation, while also playing a role in FSP1-CoQ10-NADPH pathway [208,209]. Strongest clinical evidence exists for primary mitochondrial epilepsies. A clinical case report in MELAS described improvement after CoQ10 administration [662]. In a case series in patients with *COQ8A* coenzyme CoQ10 deficiency and adult-onset SE, high-dose supplementation (3–4 g/day) resulted in a significant clinical improvement [663,664]. In acquired epilepsy, observational data links lower serum CoQ10 levels to higher seizure frequency and reduced quality of life in DRE cohorts [665,666]. CoQ10 administration and co-administration with ASM also have been shown to reduce seizure severity in animal models [281,667].

**Alpha-lipoic acid (ALA).** ALA is a mitochondrial cofactor, a broad-spectrum AOX active in aqueous and lipid phases, and a metal chelator [668]. While no controlled human trials exist for epilepsy, its clinical use is established in neuropathy, with favorable tolerability [669]. Animal studies support its potential therapeutic relevance in epilepsy. In a rat pilocarpine model, pretreatment with ALA (10 mg/kg i.p.) abolished seizures and reversed pilocarpine-associated reductions in brain Na^+^/K^+^-ATPase and δ-aminolevulinate dehydratase activity, providing a mechanistically interpretable link between metabolic enzyme preservation and seizure suppression in a cholinergic excitotoxic context [670]. In PTZ-kindled rats, ALA reduced seizure burden and improved markers of oxidative stress [671].

**Omega-3 polyunsaturated fatty acids** (PUFAs). Docosahexaenoic acid (DHA, 22:6n-3) and eicosapentaenoic acid (EPA, 20:5n-3) have well-characterized anti-inflammatory and neuroprotective profiles. DHA-derived specialized pro-resolving mediators (SPMs, including resolvins, protectins, and maresins) support active resolution of neuroinflammation while EPA and DHA compete with arachidonic acid at COX and LOX pathways, shifting synthesis away from pro-inflammatory prostanoids; their membrane incorporation may additionally modulate ion channel function [672,673]. Clinical trials of omega-3 supplementation in epilepsy have yielded mixed results. One randomized controlled trial reported a transient seizure reduction at 6 weeks with 1 g EPA plus 0.7 g DHA, which was not sustained at 12 weeks [674], whereas a subsequent larger one-year trial in 99 patients with DRE found significant reductions in seizure incidence with both EPA-rich and DHA-rich formulations [675]. Another smaller study (*n* = 21) reported negative results using 2.2 g/day combined EPA and DHA over 12 weeks [676]. In contrast, a randomized placebo-controlled crossover study in DRE reported that low-dose fish oil reduced seizures vs. placebo [677]. A triple-blind, placebo-controlled trial (360 mg EPA + 240 mg DHA, 16 weeks, *n* = 50) reported reduced seizure frequency and duration alongside decreases in serum TNF and IL-6 [678]. A 2022 systematic review and meta-analysis concluded that omega-3 supplementation reduced seizure frequency across available trials, while emphasizing heterogeneity and limited trial numbers [679]. A 2025 meta-analysis focused on DRE identified moderate-certainty evidence of benefit at 0.3–1.7 g/day, and found that larger and longer trials showed more consistent benefit [680], while a dose–response network meta-analysis suggested a non-monotonic effect [681]. Given a generally favorable safety profile, supplementation at 1–2 g/day combined EPA + DHA appears to be a reasonable adjunct where systemic inflammation is evident or omega-3 intake is low [682]. Notably, a two-sample Mendelian randomization study revealed that genetically predicted increase in blood omega-3 fatty acids levels was associated with a higher risk of epilepsy [683], complicating direct causal inference and reinforcing that omega-3 effects in epilepsy are likely phenotype- and context-dependent.

##### Vitamins and Carotenoids as Classical Antioxidants

**Vitamin E (α-tocopherol).** Vitamin E is a lipid-soluble radical scavenger that concentrates in membranes and inhibits LPO. Preclinical studies show mixed results. In one study, α-tocopherol failed to prevent seizures in maximal electroshock, PTZ threshold, and kindling paradigms, but delayed seizure onset in the iron-induced model [684]. In a rat pilocarpine model, acute and chronic treatment with α-tocopherol reduced hippocampal LPO and other markers of oxidative stress [685,686], without significant reduction in seizure frequency [686]. In one pediatric controlled clinical trial, 400 IU/day reduced seizure frequency in DRE [687]. One adult placebo-controlled crossover trial at the same dose showed no therapeutic efficacy [688]. However, another double-blind placebo-controlled trial in 65 ASM-treated patients found that 6 months of 400 IU/day reduced monthly seizure frequency and OS markers while raising catalase and glutathione [689]. 

**Vitamin** C (ascorbate). Vitamin C is a water-soluble AOX that can recycle vitamin E and directly scavenge reactive species. In mouse models, high-dose ascorbate prolonged latency to seizures [690]. Co-administration of vitamin C with vitamin E before pilocarpine SE reduced behavioral seizures and hippocampal neuron loss [691]. While clinical data on vitamin C in epilepsy remain sparse, one pediatric double-blind placebo-controlled RCT reported that a combined therapy with vitamins C and E (100 mg/day and 200–400 IU/day, 8 weeks) reduced seizure frequency compared to placebo [692].

##### Targeted Redox Pathway Modulators and Sleep-Threshold Adjuncts

**Sulforaphane** (SFN), a dietary isothiocyanate from broccoli sprouts, activates Nrf2 transcription, upregulating glutathione synthesis, SOD, catalase, heme oxygenase-1 (HO-1), and NAD(P)H quinone dehydrogenase 1 (NQO1) [203]. In a rat TLE model, post-SE SFN upregulated Nrf2 pathway gene expression, restored glutathione levels, and reduced neuronal loss in CA1 and CA3 [693]. In mice, repeated dosing elevated seizure thresholds in 6 Hz and fluorothyl seizure models and improved hippocampal mitochondrial bioenergetics but provided no protection in a PTZ tonic seizure model [694]. Notably, a separate mouse study revealed a dose constraint: at 200 mg/kg (approaching the murine LD50 of ~212 mg/kg) SFN was proconvulsant, lowering thresholds for myoclonic and clonic seizures in the PTZ and 6 Hz tests [695]. The same study found that a lower SFN dose (100 mg/kg) increased serum and brain carbamazepine concentrations and potentiated its anticonvulsant efficacy in the maximal electroshock test—a pharmacokinetic interaction that could equally raise toxicity risk in humans, given carbamazepine’s narrow therapeutic index [695]. No controlled human epilepsy trials have been conducted.

**N-acetylcysteine (NAC)** is an antioxidant with mucolytic properties used as treatment in a few medical conditions, including acetaminophen toxicity, also widely available as an OTC supplement in many jurisdictions [171,523,524,696]. In a rat kainate TLE model, oral NAC pretreatment reduced mTOR activation and restored mitochondrial fission-fusion balance [529]. In PTZ rodent studies, NAC produced dose-dependent delays in seizure onset under both acute and chronic dosing, accompanied by reduced markers of oxidative stress [527,697]. NAC also demonstrated synergistic anticonvulsant effects with valproate at multiple dose ratios and additive effects with phenytoin, while simultaneously elevating hepatic and renal glutathione, suggesting it could play a hepatoprotective adjunct role in valproate-treated patients [698,699]. In non-epilepsy clinical domains, oral NAC is generally well tolerated [700].

**Summary.** Across the dietary and supplementation approaches with clinically or pre-clinically demonstrated efficacy in the treatment of epilepsy, the shared feature of these interventions is their capacity to engage the distal pathophysiological triad. The clinical evidence varies considerably across this spectrum. The efficacy of add-on ketogenic therapy is well-supported, with some controlled trials in pediatric DRE reporting responder rates over 50% [596], albeit revealing substantial inter-individual variability and phenotype dependence. The reviewed supplementation approaches have meaningful preclinical rationale but generally lack support in epilepsy-specific controlled trials. This evidence gap could in part reflect challenges in conducting clinical trials for natural substances, such as their unpatentability [701], among others. Taken together, available preclinical and clinical evidence justifies positioning such dietary and OTC interventions in our framework not merely as “adjuncts” to ASM therapy, but as agents that contribute to raising the seizure threshold, engaging the distal triad as a primary therapeutic target rather than as a secondary property of proximal-mechanism-oriented therapy. Translating this mechanistic rationale into clinical evidence will require research designs suited to interventions that work through additive effects across multiple pathways—a methodological challenge we will discuss in Part 3.

### 2.3. Botanical Interventions in Epilepsy: Extracts and Plant-Derived Compounds

Many medicinal plant species combine antioxidant (AOX), anti-inflammatory, mitochondria-supporting, and excitability-modulating actions, but most epilepsy-specific evidence remains preclinical and is reported at the level of extracts rather than isolated compounds. This section evaluates botanicals as pharmacological candidates, not as alternatives to ASMs, and considers preparations and compounds with existing evidence for anticonvulsant effects or engagement of the inflammatory-redox-mitochondrial triad. Evidence for whole extracts is distinguished from evidence for purified constituents throughout, since compounds such as curcumin, thymoquinone, rhynchophylline, and safranal can have clearer mechanistic profiles than their parent plants, whereas an extract may contain additional active, inactive, or even proconvulsant constituents.

#### 2.3.1. *Artemisia* Genus

*Artemisia* is a large Asteraceae genus that includes wormwoods, mugworts, and sagebrushes. Several species are used in Central Asian and other traditional medical systems. *Artemisia* plants contain sesquiterpene lactones, flavonoids, phenolic acids, terpenoids, coumarins, and essential-oil constituents that may reduce OS, attenuate inflammatory signaling, and modulate GABAergic or glutamatergic transmission [702,703]. In acute seizure tests, essential oil and ethanol extract of *A. annua* increased latency to picrotoxin- and pilocarpine-induced convulsions and protected against PTZ- and strychnine-induced seizures, although the authors interpreted the overall profile mainly as CNS depressant activity [704]. *A. dracunculus* essential oil raised the threshold for maximal electroshock- and PTZ-induced seizures in a dose-dependent manner [705].

*Artemisia vulgaris* (mugwort) preparations have consistent antioxidant and anti-inflammatory evidence, although epilepsy-specific mechanistic data remain limited. Ethanol extract and phenolic-rich fractions inhibited xanthine oxidase, scavenged DPPH/ABTS radicals, suppressed LPS-induced NO production in RAW264.7 cells, and reduced carrageenan-induced paw edema in mice [706,707]. Kazakhstan populations also show high phenolic content and free-radical scavenging activity [708].

In experimental colitis, hydroalcoholic *A. vulgaris* extract increased colonic SOD activity and glutathione, and lowered MDA, TNF and NO levels, supporting systemic inflammatory-redox modulation [709]. Direct seizure evidence is narrower. A zebrafish PTZ study reported anticonvulsant activity of *A. vulgaris* preparations, but detailed links to brain OS, neuroinflammation, and mitochondrial function require clarification [710].

*Artemisia glauca*, a closely related species native to Central Asia, is chemically interesting because its extract contains coumarin derivatives, including jusan coumarin. However, no published epilepsy, neuronal injury, or standard neuroinflammatory datasets yet support seizure-threshold claims for this species [702,711]. It should therefore be treated as a future phytochemical candidate, not as an evidence-supported antiepileptic botanical. In our ongoing unpublished work, crude root extracts of *A. vulgaris* and *A. glauca* showed preliminary protective effects in PTZ-based seizure paradigms in adult CD-1 mice, supporting further evaluation of *Artemisia* preparations with defined phytochemical profiles, seizure endpoints, and inflammatory-redox readouts. Systematic evaluation of *A. glauca* in epilepsy-relevant models therefore represents a promising direction for future work.

The strongest *Artemisia* evidence comes from *A. persica*. In mice, hydroalcoholic *A. persica* extract dose-dependently raised the PTZ seizure threshold, increased GSH and SOD in brain and serum, lowered MDA, and downregulated cortical NR2A/NR2B expression. Ketamine potentiated this anticonvulsant effect, suggesting convergence between reduced NMDA-mediated excitation and improved redox status [712]. In PTZ-kindled mice, repeated *A. persica* treatment reduced seizure severity and mortality, normalized oxidative markers, and improved passive avoidance and Morris water maze performance [713]. Essential oil preparations delayed PTZ-induced seizures, reduced seizure scores, decreased NO and MDA, increased total AOX capacity, and attenuated IL-1β and TNF elevations; flumazenil sensitivity further suggested a GABA_A_ receptor component [714,715]. *A. persica* extracts thus combine proximal NMDAR/GABA_A_ modulation with distal AOX and anti-inflammatory effects. Related *Artemisia* extracts preserved mitochondrial membrane potential and reduced caspase-3 activity in H_2_O_2_-exposed PC12 cells [716], while 3,5-dicaffeoylquinic acid from *A. argyi* improved cognition and preserved mitochondrial function in trimethyltin-injured mouse brain [717].

At the level of isolated compounds, *A. indica*-derived carnosol, ursolic acid, and oleanolic acid positively modulated recombinant α1β2γ2L GABA_A_Rs and produced flumazenil-sensitive anticonvulsant, anxiolytic, and antidepressant-like effects in mice without motor impairment at active doses [718]. Ursolic acid separately reduced seizure burden, cognitive impairment, hippocampal microglial activation, TNF, IL-1β, and oxidative damage in pilocarpine-induced TLE [719]. Oleanolic acid, administered before PTZ, prolonged the latency to myoclonic jerks and clonic seizures, lowered seizure scores and increased brain GSH, SOD, catalase and acetylcholinesterase activities in mice [720].

*A. capillaris* extracts also show neuromodulatory activity, including cholinergic neuroprotection after ischemia, sedative-hypnotic effects, GABA_A_-linked chloride influx, and anticonvulsant activity in mouse seizure models [721,722,723]. These findings suggest that some *Artemisia* preparations influence both inhibitory transmission and distal inflammatory-redox mechanisms. However, *Artemisia* safety is preparation-dependent. Thujone-rich species such as *A. absinthium* contain α-thujone, which suppresses GABA_A_-mediated currents and has convulsant liability at sufficient exposure [724,725]. Chemical profiling and determination of thujone-exposure limits are therefore essential prerequisites for clinical use in epilepsy [726,727].

#### 2.3.2. *Ginkgo biloba*, *Bacopa monnieri*, *Curcuma longa*, *Nigella sativa*, and *Lavandula officinalis*

*Ginkgo biloba* leaf extract, usually studied as standardized EGb 761, has pleiotropic actions on the brain that are directly relevant to seizure threshold and epileptogenesis. In PTZ kindling, EGb 761 delayed myoclonic jerks, reduced seizure scores, attenuated kindling progression, restored brain AOX defenses, and lowered MDA and NO [728]. In lithium-pilocarpine TLE, a standardized *Ginkgo* fraction reduced spontaneous recurrent seizures, improved memory and affective behavior, and inhibited hippocampal mTOR hyperactivation [729]. Mechanistically, EGb 761 suppresses LPS-induced microglial TNF, IL-1β, IL-6, chemokines, and COX/PGE_2_-linked signaling [730,731], while also stabilizing mitochondrial membrane potential, respiratory control, ATP production, and neuronal bioenergetics [732,733,734].

However, *Ginkgo* also contains ginkgotoxin, especially in seeds and non-standardized products. Ginkgotoxin interferes with vitamin B_6_-dependent GABA synthesis and can provoke seizures [735,736]. Case reports describe seizure worsening or new-onset seizures after *Ginkgo* exposure [737,738,739]. High-dose EGb 761 increased spike-wave discharges in WAG/Rij rats [740]. Standardized EGb 761 extracts are associated with anti-inflammatory, AOX, and mitochondria-stabilizing effects in experimental TLE, whereas non-standardized products can retain ginkgotoxin and other proconvulsant constituents. The same botanical source can therefore either support or lower seizure threshold depending on the preparation.

*Bacopa monnieri* is mainly known as a cognitive enhancer, with human trials showing selected memory or attention benefits in non-epileptic cohorts [741,742,743,744].

Experimental epilepsy evidence suggests that cognitive and antiseizure effects may share redox and transmitter mechanisms [745]. Brahmi vati and a bacoside-A rich *B. monnieri* fraction delayed PTZ seizure onset, lowered seizure scores, increased brain glutathione, and reduced MDA and acetylcholinesterase activity [746]. In pilocarpine-epileptic rats, *B. monnieri* or bacoside preparations improved behavioral deficits and restored altered GABA_A_ receptor expression and function in cerebellum, hippocampus, and cortex [747,748,749]. *B. monnieri* preparations also modulated 5-HT_2C_ receptors in cerebellar and hippocampal regions, consistent with monoaminergic effects relevant to affective comorbidity and network excitability [750,751].

*Bacopa* also reduces oxidative damage and increases GSH, catalase, SOD, and GPx in cytosolic and mitochondrial brain fractions from mice fed *B. monnieri* [752]. In vitro, standardized extracts preserved mitochondrial membrane potential and complex I activity in MPP^+^/paraquat-challenged SK-N-SH neuroblastoma cells [753] and suppressed inflammatory pathways, including caspase-1 and matrix metalloproteinase-3 activity, in N9 microglial cells and cell-free assays [754]. In PTZ-exposed rats, *B. monnieri* also partially normalized cholinergic and ATPase-related parameters across brain regions, supporting broader metabolic stabilization during ictal stress [755]. Thus, the experimental evidence includes seizure-score outcomes, receptor normalization, mitochondrial redox support, and partial correction of energy-linked enzymatic disturbances, but no controlled trials have tested *Bacopa* for antiseizure efficacy in patients with epilepsy.

Curcumin, the principal curcuminoid from *Curcuma longa*, has one of the strongest preclinical datasets among plant-derived compounds. In PTZ kindling, oral curcumin (100–300 mg/kg) increased latency to myoclonic and tonic-clonic seizures, reduced seizure scores and frequency, and fully prevented PTZ-induced cognitive impairment, restored brain GSH and lowered MDA [756]. In pilocarpine SE, i.p. curcumin (100–300 mg/kg) increased seizure threshold and improved hippocampal redox balance, although MDA did not fully normalize [757]. In KA-induced TLE models, curcumin pretreatment reduced behavioral seizure expression, hippocampal neuronal loss, mossy fiber sprouting, LPO, and nitrosative injury [758]. When administered during the latent period after KA-induced SE, curcumin did not prevent spontaneous recurrent seizures, but reduced their severity and protected cognition, supporting a trajectory-modifying effect within an epileptogenesis-relevant treatment window [759]. In chronic PTZ kindling, long-term curcumin (100 mg/kg, oral) restored mitochondrial complex I and IV activities, reduced ROS, LPO, protein carbonyls, and glutathione depletion, preserved mitochondrial ultrastructure in hippocampus and cortex [760], attenuated microglial and astrocytic activation, and lowered hippocampal and cortical levels of IL-1β, IL-6, TNF and MCP-1 [761]. Studies in rat models further implicated the PPARγ/PTEN/Akt pathway and NLRP3 inflammasome-linked inflammatory signaling [762,763].

Curcumin’s poor oral bioavailability has prompted formulation strategies to improve delivery: solid lipid nanoparticles, liposomal curcumin, and micronized preparations improved seizure-related and neuroprotective outcomes in experimental models [764,765,766]. A pediatric randomized crossover trial of nanomicelle curcumin reported reduced seizure counts in intractable epilepsy, but this remains an early clinical signal requiring replication [767].

A 2025 meta-analysis of 23 rodent studies confirmed consistent effects of curcumin on seizure latency, cognitive performance, and markers of OS and inflammation [768]. Together with the pediatric nanomicelle trial [767], curcumin is the only botanical in this section with both full preclinical engagement of the inflammatory-redox-mitochondrial triad and an initial human epilepsy trial, though it is still best treated as a compound-level candidate: the positive studies used purified curcumin or defined formulations, and their effects do not extend to dietary turmeric powders with unknown bioavailability.

*Nigella sativa* seed oil and its main quinone, thymoquinone (TQ), combine inhibitory, redox, and inflammatory mechanisms. Intracerebroventricular TQ (200–400 μM) in rats delayed PTZ-induced seizure onset, shortened seizure duration, and reduced lethality; flumazenil and naloxone attenuated this effect, implicating benzodiazepine-sensitive GABA_A_ sites and opioid receptors [769]. Systemic TQ also raised seizure thresholds in PTZ and maximal electroshock models in mice and reduced mortality [770]. In PTZ kindling, dietary *Nigella* oil decreased seizure scores, limited cortical and hippocampal LPO, and restored GSH and SOD activities [771]. Later TQ studies showed suppression of PTZ kindling, reversal of memory deficits, normalization of brain oxidative/nitrosative stress markers, and lower inducible nitric oxide synthase (iNOS) expression [772,773]. TQ also activates Nrf2/ARE signaling and increases HO-1, NQO1, and GST in neurodegeneration models [774].

TQ potentiated valproate’s anticonvulsant effect in PTZ and MES models [775] and enhanced phenytoin’s anticonvulsant effect against MES-induced convulsions, partly by reducing mTOR pathway hyperactivation and neuroinflammation [776]. *N. sativa* therefore remains an experimental multi-target adjunct candidate, not a clinically validated herbal ASM: these interactions have not been tested in patients, and no studies establish a safe add-on dose or define interactions with valproate, phenytoin, benzodiazepines, or other ASMs in humans.

*Lavandula officinalis* reduced seizure scores and mortality, prolonged latency to myoclonic and generalized seizures, and lowered hippocampal NO and MDA levels in PTZ-kindled mice [777]. Lavender monoterpenes, including linalool and linalyl acetate, modulate excitability-relevant targets such as voltage-operated Ca^2+^ channels and serotonergic transporters, and protect cells from glutamatergic toxicity [778,779,780].

In non-epilepsy brain injury models, lavender oil increased SOD, GPx, catalase, and GSH, reduced LPO and DNA fragmentation, and lowered mitochondrial ROS, MDA, and protein carbonyls while preserving the GSH/GSSG ratio [781,782]. Linalool also reduces mitochondrial ROS and Ca^2+^ overload and preserves mitochondrial membrane potential under glutamate/NMDA toxicity [783]. Standardized oral lavender oil (for example Silexan) has anxiolytic efficacy in non-epilepsy trials, but epilepsy-specific trials are lacking [784,785].

*Lavandula dentata* oil and ethanol extract also modulated neuroinflammation, OS, ion-channel and electrolyte activity, and Treg/Th17-related histopathology in a pilocarpine rat model, suggesting effects that may extend beyond *L. officinalis* to other *Lavandula* species when preparations are chemically characterized [786]. However, evidence for direct anti-seizure efficacy remains entirely preclinical, and essential-oil composition varies strongly across species, chemotypes, and extraction procedures, which complicates dose translation. Lavender’s anxiolytic and sleep-related actions may be useful for patients whose seizures are stress- or sleep-sensitive, but this represents an indirect threshold-stabilizing rationale rather than evidence of antiseizure efficacy in humans.

#### 2.3.3. Additional Botanicals with Complementary Distal and Proximal Mechanisms

Several plant-derived polyphenols converge on inflammatory-redox hubs, especially NF-κB suppression, Nrf2 activation, and mitochondrial protection [787].

**Resveratrol**, a stilbene abundant in grapes and berries, increased latency to generalized PTZ seizures and potentiated the anticonvulsant effects of valproate and diazepam. This effect was reversed by a nonselective adenosine receptor antagonist (theophylline) but not by a selective A2 receptor antagonist [788]. In PTZ kindling and KA models, it reduced seizure scores, neuronal injury, cognitive and affective deficits, hippocampal cytokines, oxidative stress, and mitochondrial dysfunction, with mechanisms involving SIRT1/PGC-1α, Nrf2, and inflammatory cytokine suppression [243,789,790,791,792,793,794,795].

**Quercetin**, a flavonoid abundant in the flavonol glycoside fraction of standardized *Ginkgo biloba* extract, dose-dependently increased seizure latency and reduced seizure severity in PTZ and kindling models, improved survival, and lowered brain TNF, IL-1β, LPO, and NF-κB activation, with effects varying by dose, schedule, and model [796,797,798,799,800]. Its neuroprotective and Nrf2-linked mechanisms have been reviewed elsewhere [801,802,803,804].

**Flavonoid luteolin** reduced kainate- or PTZ-induced seizures, hippocampal glutamate accumulation, microglial activation, neuronal death, and TLR4/IκBα/NF-κB signaling, improved spatial memory, with recent evidence for GADD45B-linked stress-response modulation [805,806,807].

**Green tea catechins**, especially **(-)-epigallocatechin-3-gallate (EGCG)** reduced PTZ-kindling severity and cognitive deficits, normalized OS markers in hippocampus, suppressed the hippocampal TLR4/NF-κB/IL-1β signaling after lithium-pilocarpine SE, and decreased interictal-like activity ex vivo [808,809,810,811,812]. Enhanced delivery may increase efficacy, as EGCG-functionalized selenium nanoparticles showed stronger anticonvulsant, AOX, anti-inflammatory, and anti-apoptotic effects in PTZ seizures [813].

Whereas the evidence for compounds discussed above is mechanistically anchored primarily in the distal inflammatory-redox-mitochondrial triad, the remaining botanicals in this section add to that more proximal, receptor- or channel-specific mechanistic evidence.

**Valerian root** is traditionally used as a sedative and sleep aid. Pharmacological studies demonstrate that valerenic acid and related sesquiterpenes act as positive allosteric modulators at GABA_A_Rs; valerenic acid and *Valeriana officinalis* extracts delayed PTZ seizures in zebrafish and mice, although controlled epilepsy trials are absent [814,815,816,817,818].

*Passiflora incarnata* extracts elicit dose-dependent GABA_A_ currents in hippocampal neurons; in vivo, anticonvulsant effects against PTZ-induced seizures were seen with some but not all extraction methods, while behavioral testing found anxiogenic rather than anxiolytic effects across extracts [819]. In rodent seizure models, hydroalcoholic *P. incarnata* preparations increased seizure latency and reduced PTZ-induced convulsions, with pharmacological dissection implicating benzodiazepine-site and opioid-receptor components in some designs [820,821]. However, a 2025 comparative extract study reported only modest, non-significant electroshock protection despite biochemical anti-inflammatory and AOX signals [822]. This discrepancy reinforces that preparation, dose, and model selection can determine whether favorable biomarker-level activity translates into seizure suppression.

The Chinese herb *Uncaria rhynchophylla* (Gouteng) contains rhynchophylline and isorhynchophylline, tetracyclic oxindole alkaloids and noncompetitive NMDAR antagonists [823]. In KA and pilocarpine TLE models, *U. rhynchophylla* extract or rhynchophylline reduced behavioral and electrographic seizures, hippocampal injury, S100B/RAGE signaling, IL-1β, BDNF changes, persistent sodium current (I_NaP_), and NR2B-related signaling [824,825,826,827]. Rhynchophylline thus links plant-derived pharmacology to NMDAR and persistent sodium current, not only to nonspecific AOX effects. 

**Safranal**, a constituent of saffron (*Crocus sativus*), reduced PTZ-induced seizure duration, delayed the onset of tonic convulsions, and protected against seizure-related death in mice [828]. In rats, safranal’s protection against PTZ-induced seizures involved GABAergic and opioid-system components [829], and a 2024 PTZ study found that safranal reduced seizure severity and hippocampal injury while suppressing NF-κB signaling and mitochondrial apoptosis markers [830]. **Crocin**, another saffron carotenoid, was ineffective in some acute PTZ conditions [828], but later reduced kindling, SE, spontaneous recurrent seizures, hippocampal TNF, and neuronal loss in TLE models, while increasing cortical BDNF [831,832].

**Rosmarinic acid**, a phenolic constituent of *Melissa officinalis* and other Lamiaceae prolonged seizure latency and reduced seizure incidence in PTZ and pilocarpine models [833,834], while *Melissa officinalis* essential oil reduced PTZ-kindling severity, mortality, brain NO, and MDA [835].

Kava (*Piper methysticum*) and its **kavalactone**-rich preparations delayed PTZ seizures in zebrafish [836], but despite its historical use for convulsions, concerns regarding potential hepatotoxicity and other toxic effects limit its suitability for chronic use in epilepsy [837].

Together, these compounds and botanicals illustrate two evidentiary patterns. Resveratrol, quercetin, luteolin, and EGCG converge on the same distal NF-κB/Nrf2/mitochondrial triad addressed throughout this section, with effects reproduced across multiple PTZ- and kainate-based models. *Valeriana*, *Passiflora*, *Uncaria*, saffron/crocin, rosmarinic acid, and kava instead act through more specific proximal targets—GABA_A_ and NMDAR modulation, persistent sodium-current suppression, and benzodiazepine- or opioid-site interactions—though several also show that preparation and extraction method can determine whether biochemical or receptor-level activity translates into a measurable anticonvulsant effect, as with *Passiflora*’s extraction-dependent reversal from anxiolytic to anxiogenic outcomes and the non-significant electroshock protection reported in [822]. None of the compounds reviewed in this subsection has been tested in a controlled human epilepsy trial, and isolated-compound efficacy should not be assumed to extend to chemically undefined extracts from parent plants or multi-herb formulations.

#### 2.3.4. Clinical Perspective

From a clinical perspective, two issues deserve emphasis: herb–drug interactions and quality control. Many of the plants discussed here contain multiple bioactive constituents that can inhibit or induce cytochrome P450 enzymes or affect drug transporters, which creates a realistic risk of pharmacokinetic and pharmacodynamic interactions with ASMs. Classical examples include *Ginkgo biloba* preparations that have precipitated breakthrough seizures in previously stable, well-treated patients [737,738]. Proposed mechanisms include ginkgotoxin, a vitamin B_6_ antagonist concentrated in Ginkgo seeds, and CYP2C19-mediated induction that lowered serum phenytoin and valproate levels in one reported case [738]. Broader surveys of herb–drug interactions show that many reports are poorly documented yet still identify a subset of well-substantiated cases, supporting a cautious stance when combining herbal products with drugs that have a narrow therapeutic index such as ASMs [838,839,840]. In addition, variability in plant species, growing conditions, extraction methods and labeling, as well as occasional contamination or adulteration, means that the actual content of commercial preparations may differ substantially from what is assumed from product labeling or study specifications [841].

In terms of clinical evidence, only a few plant-derived interventions have been tested in controlled epilepsy trials. The clearest example is purified cannabidiol from *Cannabis sativa*, which in randomized controlled trials for Dravet syndrome produced a roughly 20–25 percentage point greater reduction in convulsive seizure frequency than placebo and a responder rate of about 40% for a 50% seizure reduction [389]. This level of evidence contrasts sharply with most other herbs considered in this section. For *Artemisia* spp., *Bacopa monnieri*, *Curcuma longa* (curcumin), *Nigella sativa*, *Lavandula* spp., *Ginkgo biloba* and others, the evidence base remains almost entirely in vivo and in vitro, with no adequately powered, blinded trials in people with epilepsy [841,842]. Cannabidiol itself illustrates another translational issue, since its efficacy is tightly linked to highly standardized formulations and it shows clinically relevant interactions with clobazam and other drugs through CYP2C19 and CYP3A4-related mechanisms, with liver-enzyme monitoring required in some co-medication contexts [389].

Taken together, experimental studies suggest that extracts of *Artemisia* species, *Ginkgo biloba*, *Bacopa monnieri*, *Curcuma longa*, *Nigella sativa*, *Lavandula* spp., and several other medicinal plants can lower seizure incidence or severity in animal models, often while preserving or improving cognitive performance. Mechanistically, these interventions converge on AOX and anti-inflammatory pathways, including suppression of NF-κB signaling and activation of Nrf2-dependent cytoprotective genes, together with modulation of ion channels and neurotransmitter systems such as GABA_A_Rs and NMDARs [243,520,796,843]. Polyphenols such as curcumin and resveratrol, and flavonoids such as quercetin and luteolin, also engage mitochondrial function and biogenesis, which links herbal approaches directly to the redox and bioenergetic mechanisms discussed earlier in this review [243,520,796].

However, these herbs cannot yet be considered stand-alone antiepileptic treatments. Human data are scarce, dosing regimens are poorly standardized, and the pharmacokinetic behavior of complex mixtures is largely unknown. Side effects range from relatively mild gastrointestinal or sedative complaints (for example with *Bacopa* or lavender) to more serious concerns such as hepatotoxicity with kava and proconvulsant or neurotoxic constituents in some *Artemisia* and *Ginkgo* preparations [737,738,838,839].

Unlike the dietary and supplemental approaches discussed above, herb–drug interaction risk means the herbal compounds cannot simply be recommended as add-ons to ASM therapy; their pharmacology and interaction profile need to be characterized before clinical use. Many of the reviewed herbal and polyphenolic compounds combine defined anticonvulsant activity with anti-inflammatory and antioxidant engagement of the distal triad. Future work should move beyond proof-of-concept animal studies toward isolation of active constituents, systematic screening for anticonvulsant activity, rigorous pharmacological and interaction characterization, and well-designed clinical trials in defined epilepsy syndromes to support new antiepileptic drug development.

## 3. Part 3: Translational Frameworks for Threshold Management in Drug-Resistant Epilepsy

Parts 1 and 2 assembled substantial mechanistic evidence: neuroinflammation, oxidative stress, and mitochondrial dysfunction form a tightly interconnected distal triad that progressively lowers seizure threshold and may contribute to pharmacoresistance, and a range of pharmacological, dietary, botanical, and nutritional interventions can engage these pathways. Yet anti-inflammatory, antioxidant, and mitochondria-directed approaches remain peripheral to epilepsy clinical practice—even in DRE, where both the need and the mechanistic rationale are greatest. Part 3 addresses this translational gap. We argue that the barriers are not primarily evidential but structural: the absence of a unified conceptual framework integrating distal pathophysiology, precipitating factors, and patient agency; inadequate infrastructure for stratifying which patients benefit from which interventions; and trial designs poorly suited to multicomponent, context-dependent interventions. Section 3.1 develops the unified framework that positions every therapeutic class within a single coherent model of threshold regulation rather than viewing ASM as the central strategy with everything else as adjuncts. Section 3.2 examines the structural barriers—biomarker validation, drug development, and trial design—and the methodological innovations required. Section 3.3 synthesizes the research agenda and considers opportunities for incremental clinical implementation.

### 3.1. Threshold Dynamics: Precipitating Factors, Patient Agency, and the Expanded Reservoir Analogy

Parts 1 and 2 established how the distal triad lowers seizure threshold and drives pharmacoresistance. Two further elements complete the picture: precipitating factors, which act as acute amplifiers of the same chronic vulnerabilities, and the patient’s capacity to monitor and modify them directly. This section integrates both with the distal pathophysiological triad into a unified threshold management framework, culminating in the revised reservoir analogy (Section 3.1.3).

#### 3.1.1. Precipitating Factors and the Reflex–Spontaneous Continuum

Section 1.1 introduced the distinction between proximal and distal causal factors in epilepsy, which collectively modulate the seizure threshold. While this framework captures genetic, structural, and chronic pathophysiological contributors to threshold lowering, it incompletely addresses the temporal dynamics of seizure occurrence: why does a patient with chronically low threshold experience a seizure at this particular moment rather than another? This question directs attention to precipitating factors, acute perturbations that interact with chronic vulnerabilities to push threshold-lowered neural networks into seizure activity.

Traditional epilepsy nosology divides seizures into reflex seizures, which are “objectively and consistently evoked by a specific afferent stimulus or by activity of the patient,” and spontaneous seizures, which occur without identifiable immediate triggers [43,844]. At one end of this continuum lie classic reflex seizures with highly observable, reproducible extrinsic triggers, such as photosensitive seizures, hot-water epilepsy, or reading epilepsy; at the other lie seizures conventionally labeled “spontaneous” but which may be precipitated by triggers that are intrinsic, complex, temporally delayed, or simply unobserved [43,844]. However, as Irmen et al. (2015) point out, this dichotomy may be artificial: “reflex seizures and spontaneous seizures may be the two extremities of a conceptual continuum on which seizures are generated by extrinsic or intrinsic triggers,” with the distinguishing feature being not the presence or absence of triggers but rather their observability, reproducibility, and complexity [43].

##### Clinical Evidence and Relevance

In a systematic study of adult epilepsy clinic patients, Ferlisi and Shorvon (2014) found that 97% of patients could identify at least one seizure precipitant [14]. The most frequently reported precipitants were stress (82%), sleep deprivation (71%), and fatigue (68%) [14]. Other commonly cited factors included hormonal changes during the menstrual cycle, fever, alcohol consumption, missed medications, and specific emotional states. The distribution of precipitating factors also varies across epilepsy syndromes: patients with psychological comorbidities reported a significantly higher percentage of seizures with identifiable precipitants; patients with extratemporal epilepsy more frequently report seizures during sleep; and TLE patients more commonly identify emotional or cognitive precipitants [14].

In a study of 150 children with DRE, Aird (1988) reported that by manipulating seizure-inducing factors, it was possible to achieve about 50% seizure reduction in 20% and complete seizure control in 14% of patients [845]. “Reviews of the literature have suggested that this more comprehensive approach to the therapeutic management of epilepsy has not been adequately exploited,” he wrote in 1988—a statement that largely holds true to this day [845].

##### Mechanistic Links: Precipitants as Acute Activators of Distal Pathophysiology

**Figure 3:**
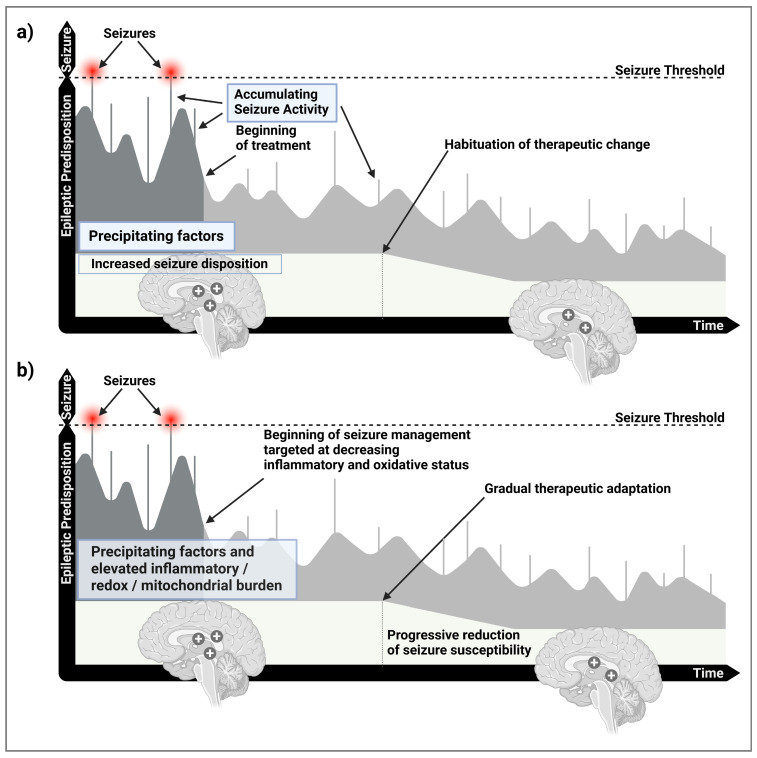
Adaptation and mechanistic remapping of the Tang et al. threshold model. (**a**) Author-redrawn, integrated version of the original Tang et al. (2014) [846] two-panel model, combining fluctuating seizure activity, seizure threshold, precipitating factors, and epileptic predisposition over time, together with the treatment/habituation elements, into a single panel. (**b**) Mechanistic reinterpretation in which acute precipitants act on a background of elevated neuroinflammatory, oxidative, and mitochondrial burden. Anti-inflammatory and antioxidant management is depicted as progressively lowering seizure susceptibility by reducing this distal burden. (Created in BioRender. Trofimov, A. (2026) https://BioRender.com/lfrolc1 accessed on 13 July 2026).

Importantly, precipitating factors are not completely independent of the chronic distal vulnerabilities described in Part 1. Instead, they often function as their acute exacerbators. A patient with lower baseline neuroinflammation and OS requires a larger perturbation to cross the seizure threshold and vice versa. This means that interventions targeting baseline distal pathology should theoretically raise the threshold for precipitant-triggered seizures as well as spontaneous ones—a prediction compatible with the dynamic threshold model of Tang et al. (2014) [846] (Figure 3a) in which seizure occurrence reflects the interaction between baseline epileptic disposition and superimposed precipitating factors over time, here extended to highlight the role of inflammatory, oxidative, and mitochondrial burden (Figure 3b). Below, we will briefly review evidence linking the most common precipitants to the distal triad.

**Psychological stress.** Acute psychological stress activates the hypothalamic–pituitary–adrenal axis, while chronic psychological stress increases proinflammatory cytokine production, elevates ROS, and dysregulates glutamatergic neurotransmission [847]. In animal models, injections of corticotropin-releasing factor produce seizures, and corticosterone often exhibits proconvulsant activity [848]. In humans, self-reported stress predicted seizures in the next 24 h in a dose-dependent manner [41]. A 2025 systematic review concluded that higher stress levels cluster with poorer seizure control, polytherapy, depression, and lower social support—a pattern consistent with stress acting as a chronic amplifier of baseline network vulnerability [849].

**Sleep deprivation** has been extensively studied at the network level. In the *Kcna1*^−/−^ mouse model, repeated sleep deprivation exacerbated seizures and reduced GABAergic tonic inhibition in dentate granule cells [850], thereby directly affecting the E/I balance. In parallel, sleep deprivation also acts through distal pathways. A single night of total sleep deprivation increases circulating IL-6 levels and activates NF-κB signaling pathways in peripheral blood monocytes [851,852], while chronic partial sleep restriction produces sustained elevation of inflammatory markers and OS [853], mirroring the chronic inflammatory and oxidative vulnerabilities that maintain low seizure thresholds in active epilepsy [42].

**Fever**, which is particularly relevant in pediatric epilepsy, triggers pyrogenic cytokine release and activates immune cascades [854]. In patients with pre-existing inflammatory and oxidative burdens, this represents a compounded metabolic and inflammatory challenge. Temperature elevation and infection-related inflammation contribute in parallel: hyperthermia alone can precipitate seizures in susceptible networks, while IL-1 family cytokines independently amplify excitability through immune–neuronal coupling [854,855]. Dravet syndrome illustrates the interaction between genetic vulnerability (SCN1A mutations affecting sodium channel function) and acute metabolic perturbations [856]. Recent genetic studies implicate PGE2–EP3 signaling, IL10, and synaptic machinery including GABRG2 and SCN1A/SCN2A [857].

**Hormonal fluctuations** during the menstrual cycle, reported as a precipitant by a substantial minority of women with epilepsy (catamenial epilepsy) have a well-characterized proximal mechanism involving neurosteroid modulation of GABA_A_ receptors [858,859]. On the other hand, the perimenstrual period involves changes in inflammatory cytokine profiles, and neurosteroid withdrawal may interact with inflammatory signaling [860,861,862,863], illustrating that even precipitants with well-established proximal mechanisms are not necessarily distal-independent.

#### 3.1.2. Patient Agency in Threshold Management: From External to Internal Locus of Control

The evidence reviewed in 3.1.1 has a direct implication for patient agency in epilepsy, where seizures represent abrupt, involuntary loss of bodily autonomy. Locus of control (LOC)—the degree to which individuals believe they can influence events affecting their lives [864]—has been studied since the 1980s, primarily as a psychosocial variable influencing quality of life and treatment engagement [865,866,867,868]. However, the mechanistic view established above reframes and extends the significance of LOC in epilepsy. If psychological stress chronically amplifies neuroinflammatory and oxidative burden, as reviewed above, then reducing perceived uncontrollability has a direct impact on the seizure threshold, making internal LOC and self-efficacy [869] not just coping strategies but threshold-modifying factors.

This framing is consistent with existing evidence. A 2020 systematic review synthesizing over 20 studies found that internal LOC in epilepsy correlates with better self-efficacy, adaptive coping, and higher quality of life [867,870]. A structured interview study of 100 adults with poorly controlled epilepsy reported that over 90% identified at least one seizure precipitant, 52% reported consciously trying to avoid precipitants, and 47% said they could sometimes stop their seizures from happening [871], while systematic trigger tracking has been shown to generate a subjective sense of control [872]. Importantly, higher self-efficacy—confidence in one’s ability to execute threshold-relevant behaviors—is associated with better medication adherence and lifestyle-management behaviors [873,874], and structured self-management programs have demonstrated improvements in seizure frequency, quality of life, and medication adherence in patients who can identify precipitants or prodromes [875,876,877].

The scope of patient agency also maps onto the observability gradient implicit in the reflex-spontaneous continuum. At the most observable end, highly observable triggers, such as photosensitivity, afford straightforward avoidance with clear cause-and-effect relationships [43,878]. Less observable but identifiable precipitants, such as sleep deprivation and stress, require sustained self-monitoring but remain directly accessible to patient initiative; these are also the factors that most acutely activate inflammatory and oxidative pathways [14,41,851,879]. At the least observable end lie the chronic distal vulnerabilities detailed in Part 1: not directly perceivable but addressable through anti-inflammatory diet, exercise, and avoidance of proinflammatory or prooxidant exposures [880,881,882,883].

Patient agency thus extends across the full continuum from identifiable triggers to addressing the chronic biological terrain that determines whether any given perturbation tips the network into seizure. Mechanistic grounding of the distal triad should, in principle, support adherence and self-efficacy. When patients understand that behavioral factors not only serve as acute triggers but also activate the pathophysiological mechanisms that maintain chronically low thresholds, this further strengthens the rationale for self-management strategies. This framing also positions patients as contributors to research: systematic self-monitoring of threshold-relevant exposures and seizure outcomes generates personalized data that conventional trial designs cannot capture and that, aggregated, could feed the phenotyping frameworks and N-of-1 designs discussed in Section 3.2.

#### 3.1.3. Revisiting Lennox and Lennox’s ‘Reservoir’ Analogy

In recent years, it has become common to represent the concept of epileptic threshold with a graph plotting time against “epileptic predisposition” or “seizure probability” with a fixed line representing the “seizure threshold” [846,879,884,885]. Cumulative predisposing factors are visualized as a fluctuating curve, like a mountainous landscape, that approaches and recedes from the threshold line above, with periodic triggering events creating sharp spikes. When these spikes cross the threshold line, seizures occur (Figure 3). A variation in this figure has been adopted by many educational resources intended for the general public. This visualization effectively shows temporal variability in predisposition and resilience. However, it suffers from a fundamental conceptual flaw: the threshold appears as a static, separate entity hovering above the very factors that determine it. In reality, the cumulative burden of predisposing factors is itself the threshold; they are the same phenomenon, not distinct entities. Furthermore, this visualization fails to capture the fact that the threshold itself is modifiable, appearing constant when it is in fact dynamically shaped by the pathophysiological processes we have discussed.

**Figure 4:**
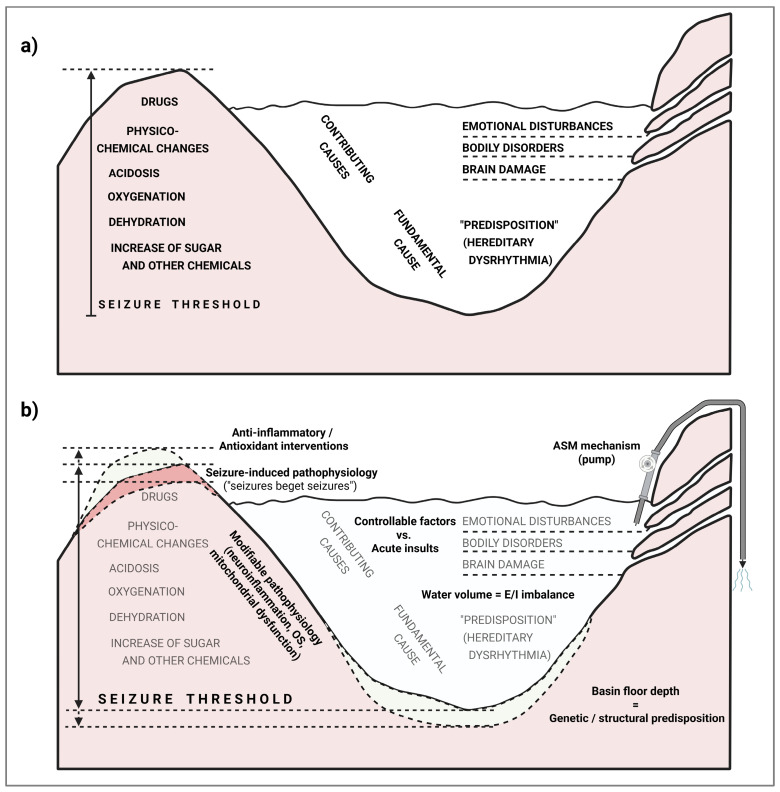
Seizure threshold ‘reservoir’ conceptualization. (**a**) Author-redrawn refinement of the original ‘reservoir’ model, proposed by Lennox and Lennox-Buchthal (1960) [17] and reproduced in Shorvon et al. (2014) [15], preserving its overall geometry while clarifying the relation among seizure threshold, contributing causes, and predisposition. (**b**) Conceptual remapping of the ‘reservoir’ analogy in which water volume represents acute excitation/inhibition imbalance, basin floor depth represents genetic or structural predisposition, and bank height represents modifiable distal pathophysiology, including neuroinflammation, OS, and mitochondrial dysfunction. Tributary inflows represent controllable factors and acute insults. Overflow corresponds to seizure occurrence, while recurrent seizures further erode the banks, increasing future seizure susceptibility. Antiseizure medications primarily lower effective water load, whereas anti-inflammatory and antioxidant interventions reinforce or rebuild the banks. (Created in BioRender. Trofimov, A. (2026) https://BioRender.com/lfrolc1 accessed on 13 July 2026).

An analogy that better captures the complex causation in epilepsy, and reveals opportunities for therapeutic intervention, is the ‘reservoir’ concept originally proposed by Lennox and Lennox-Buchthal (1960) [17] and reproduced in Shorvon et al. (2014) [15]. However, we propose a conceptual revision that more accurately reflects current understanding of epilepsy pathophysiology—we show both the original analogy (Figure 4a) and our proposed refinement (Figure 4b). Table 2 explicitly maps the relevant concepts related to epilepsy to different parts of the reservoir image.

The original Lennox and Lennox reservoir analogy depicts a water basin with a bank on one side and a slope on the other, with streams flowing in to supply additional water. When water rises above the bank and overflows, this represents a seizure. As described by Shorvon et al. (2014) [15]: the threshold is the *bank*; *water already present* in the reservoir (below the level of incoming streams) represents the person’s predisposition; *supplying streams* represent contributory conditions such as acquired brain lesions, bodily function disorders, and emotional disturbances; the *bank height* is shaped by metabolic factors: glucose levels, oxygenation, hydration status, acid-base balance, physicochemical changes, and drugs; *overflow* represents seizure occurrence [15].

We propose the following refined mapping: the threshold is *the entire basin*—consisting of both bedrock depth and bank height—not the bank alone; *basin floor depth* represents genetic and structural factors (distal, largely immutable predisposition); *bank height* represents the metabolic, inflammatory, and oxidative state (distal but modifiable through the interventions discussed in Part 2); *water volume* represents the current excitation/inhibition balance (proximal); *tributary inflow* represents both controllable factors (precipitants, behavioral triggers) and acute insults (e.g., head injury, acute infection); *overflow* represents seizure occurrence (Table 2).

This revision better integrates current understanding of epilepsy pathophysiology. First, a person with epilepsy has, by definition, a shallower overall basin, requiring less inflow to trigger overflow. The basin’s reduced capacity reflects both immutable factors (i.e., genetic) and modifiable factors (bank height diminished by ongoing neuroinflammation, OS, and other factors). Second, the same analogy extends naturally to provoked seizures in non-epileptic individuals: acute extreme insults (severe hypoglycemia, acute sodium dysregulation, high fever) represent sudden massive tributary inflow that can cause overflow even in a deep, well-maintained basin.

Critically, this analogy captures the bidirectional nature of epilepsy causation discussed in Section 1.6: seizures themselves erode the banks through seizure-induced neuroinflammation and OS, progressively shallowing the basin (“seizures beget seizures”).

**Table 2:** Remapping the reservoir analogy.

Epilepsy Concept	Original Lennox-Lennox Analogy	Revised Analogy
Seizure threshold	Bank (fixed height)	Entire basin (bedrock depth + bank height)
Genetic/structural predisposition	Water already present below stream level	Basin floor depth (immutable)
Modifiable pathophysiology (neuroinflammation, OS, mitochondrial dysfunction)	Bank height (shaped by metabolic factors)	Bank height (explicitly modifiable)
Acute excitation/inhibition imbalance	(not clearly distinguished)	Water volume
Precipitants/acute insults	Supplying streams (undifferentiated)	Tributary inflow (split: controllable vs. acute insults)
Seizure occurrence	Overflow	Overflow
Seizure-induced pathophysiology (“seizures beget seizures”)	(absent)	Seizures erode the banks
ASM mechanism	(absent)	Pumping water out
Anti-inflammatory/AOX interventions	(absent)	Reinforcing/rebuilding banks

The revised analogy also makes it visible why current treatment paradigms are incomplete and suggests a more comprehensive approach. In this conceptual framework, traditional ASMs *pump* water out after it begins rising (symptomatic management of proximal excitation/inhibition imbalance); precipitant identification and management control tributary flow (behavioral and environmental modifications to reduce acute inflow); anti-inflammatory, AOX, and metabolic interventions reinforce and rebuild eroded banks (address distal pathophysiology to restore threshold capacity).

The revised analogy aligns with the emerging narrative that sees ASMs as symptomatic treatment [4,5]. They provide essential acute management, but do not repair or reinforce the eroded banks, which are the underlying pathophysiology that determines threshold capacity. The interventions discussed in Part 2, in contrast, target bank reinforcement. This explains why threshold-modifying interventions may show benefit even when they do not fully prevent seizures: they increase basin capacity, requiring greater provocation for overflow.

We propose that this revised reservoir analogy provides a shared conceptual framework useful for both clinical reasoning and patient education, making visible the rationale for integrative approaches that address both proximal and distal pathophysiology.

### 3.2. Toward Personalized Threshold Management: Phenotyping, Drug Development, and Trial Design

Section 3.1 integrated the distal triad, precipitating factors, and patient agency into a unified threshold management framework. Translating that framework into practice depends on two further structural gaps: how patients are characterized, which drugs get developed, and how trials are designed. Here we examine each gap through the lens of the threshold management framework.

#### 3.2.1. Biomarker Assessment and Phenotyping

The three-domain structure of the distal triad implies that patients may differ in the relative prominence of each pathophysiological domain, and that these differences could guide personalized intervention selection [46,82,230,886]. A patient with predominantly inflammatory pathology might benefit most from anti-inflammatory treatment or interventions, while one with prominent OS might respond preferentially to AOX strategies. This section examines the feasibility and limitations of mechanistic phenotyping, particularly in DRE [887,888].

Mechanistic phenotyping assumes that patients differ meaningfully in which pathophysiological domain predominates, and that these differences are detectable through biomarkers. Candidate markers exist for each domain of the distal triad: inflammatory cytokines (IL-1β, IL-6, TNF) in serum or CSF [886,889] and activated microglia on PET neuroimaging to visualize neuroinflammation in vivo [94,890,891,892]; lipid peroxidation markers (MDA, 4-HNE) and AOX capacity (GSH/GSSG) for OS [63,171,185]; and CSF lactate-to-pyruvate ratios or magnetic resonance spectroscopy of N-acetylaspartate for mitochondrial dysfunction [893,894].

The central methodological obstacle is not the absence of markers but their interpretation. Elevated biomarker levels reflect both chronic pathophysiological burden and acute seizure activity since causality is bidirectional: inflammation and OS drive seizures, but seizures also acutely elevate both [887,888]. A cross-sectional measurement therefore cannot reliably distinguish chronically high vulnerability from a post-seizure spike. Addressing this requires standardized interictal measurement windows (e.g., ≥72 h post-seizure) to capture background rather than acute response, and longitudinal tracking within the same patient to separate trait from state.

Most patients will likely present with mixed profile rather than a single-domain phenotype, consistent with the mechanistic overlap described in Section 1.6 and requiring multimodal rather than single-target intervention. The most efficient path to accumulating the phenotyping data the field requires is to incorporate standardized biomarker panels as secondary outcomes in trials of distal mechanism-targeted interventions, where they simultaneously establish measurement norms and assess target engagement [887,888].

#### 3.2.2. Incorporating Mechanistic Considerations in ASM Development

ASM development over the past three decades has focused almost exclusively on proximal mechanisms, such as ion channel function, neurotransmitter modulation, and synaptic transmission. This has generated numerous compounds with diverse mechanisms yet failed to change long-term outcomes in DRE [9,887]. At the same time, many ASMs exert pro-inflammatory and pro-oxidative effects (Table S1) that may themselves contribute to pharmacoresistance. A rational step that follows from the proposed threshold management framework is to incorporate distal mechanisms into development strategies through three complementary approaches. First, novel dual-action compounds could deliberately combine traditional anticonvulsant mechanisms with anti-inflammatory or AOX properties (for example, sodium channel blockade with Nrf2-Keap1-ARE pathway activation [80,895])—a profile already documented for valproate, levetiracetam, and perampanel [894]. Second, systematic screening and repositioning of approved anti-inflammatory and AOX agents with established safety profiles offers a faster route to clinical testing. Several candidates (celecoxib, minocycline, aspirin), already show anticonvulsant properties preclinically [888,896], and trials should incorporate biomarker-driven patient selection and target-engagement endpoints alongside seizure frequency outcomes [887]. Botanical compounds with established safety profiles, including those detailed in Section 2.3, could be prioritized for rigorous clinical evaluation. Third, mechanistic phenotyping could guide rationally assembled combination therapies—anti-inflammatory ASMs paired with anti-inflammatory agents for high-inflammatory phenotypes, AOX-favorable ASMs combined with AOX agents or interventions for OS-predominant patients—as a principled, mechanistically grounded alternative to empirical polypharmacy.

#### 3.2.3. Clinical Trial Design for Threshold-Modifying Interventions

Despite strong mechanistic rationale and supportive preclinical data, high-quality evidence for interventions targeting distal mechanisms remains relatively scarce, reflecting both the general methodological challenges of evaluating complex interventions and the specific design difficulties of epilepsy trials [897,898,899,900]. The standard randomized controlled trial (RCT) assumes a single active intervention with direct, measurable effects on outcomes, standardizable across participants and detectable within feasible follow-up periods [897,901]. These assumptions hold reasonably well for conventional ASMs but poorly for threshold-modifying distal-mechanism interventions. Rather than directly suppressing seizure generation and producing rapid, large effects, they may yield smaller incremental benefits, requiring longer observation periods and sustained behavioral adherence [897,899,900]. Null or mixed results from nutritional, antioxidant, or behavioral single-component trials [586,676,688,902] are therefore better interpreted as methodological mismatch than as disproving the underlying mechanistic hypotheses. Omega-3 fatty acid supplementation trials, for example, show results consistent with modest, heterogeneous effects requiring adequate dose range, duration, and patient selection [674,675,676,678,679,680].

While the traditional RCT asks: “Does intervention X reduce seizure frequency compared to placebo?”, for threshold-modifying interventions, the more appropriate question may be: “Does a multicomponent program addressing inflammation, OS, mitochondrial dysfunction, sleep hygiene, stress management, and precipitant avoidance raise seizure threshold sufficiently to produce clinically meaningful benefit?” This approach acknowledges additive effects across multiple domains and does not require any single component to carry the full therapeutic burden [897,899]. Several methodological approaches are better suited to answering this question. 

**Pragmatic randomized trials** comparing a comprehensive threshold management program to standard care would allow implementation flexibility while maintaining experimental control through randomization [901,903]. Primary outcomes would be ≥50% responder rates over 6–12 months, with secondary outcomes including quality of life, biomarker changes, and treatment satisfaction. **N-of-1 trial designs**, in which individual patients serve as their own controls through multiple crossover periods, address inter-individual variability [904,905,906]. **Adaptive platform trial designs** could evaluate multiple threshold-modifying interventions simultaneously, with biomarker stratification guiding allocation: patients with high inflammatory markers could be preferentially assigned to anti-inflammatory arms and those with OS phenotypes to AOX-oriented interventions, sharing control groups and enabling multi-hypothesis testing within a single trial infrastructure [907,908].

All of these designs share a common prerequisite: systematic tracking of seizure occurrence alongside the multiple variables that influence seizure threshold. Current practice relies on patient recall and seizure diaries with variable compliance [909,910,911]—methods poorly suited to capturing precipitant patterns, medication adherence, and lifestyle variables that threshold management requires. Smartphone applications designed for comprehensive threshold tracking, where patient data could be synchronized with clinical data, could address this gap while simultaneously generating research datasets of greater scale and granularity than conventional trials typically produce [912,913,914,915,916,917,918,919,920]. Several epilepsy-specific seizure tracking applications currently exist, though none fully implements the threshold management framework proposed here [913,914,915,921,922]. Methodological challenges for observational use include confounding, selection bias, and variable data completeness, requiring careful privacy and consent frameworks [912,923]. Triangulating RCT evidence with real-world observational data offers the most comprehensive path toward establishing which threshold-management strategies work in which combinations, for which patients, and under what conditions [897,899,924].

### 3.3. Integrating the Framework: From Mechanisms to Management

The framework developed across this review generates a set of priorities spanning basic science, clinical trial methodology, and health technology.

1. **Biomarker validation and standardization**. Current inflammatory, OS, and mitochondrial dysfunction markers require validation in large, well-characterized epilepsy cohorts with standardized measurement protocols. Establishment of reference ranges, clinically meaningful thresholds for phenotype categorization, and assessment of biomarker stability over time are prerequisites for mechanistic phenotyping [185,886,925,926,927].
2. **Phenotype-treatment matching studies**. Do patients with elevated inflammatory biomarkers respond preferentially to anti-inflammatory treatments and interventions? Do those with OS predominance show superior responses to AOX strategies? Trials stratifying patients by mechanistic profile and evaluating differential treatment responses would validate the phenotyping approach and guide clinical implementation [887,888,928].
3. **Drug development and botanical validation incorporating distal mechanisms**. Systematic screening of existing anti-inflammatory and AOX agents for anticonvulsant activity, design of novel dual-mechanism compounds, and rigorous clinical evaluation of botanical compounds with established preclinical profiles—with standardized active compound content and biomarker-stratified efficacy trials—represent complementary near-term directions [46,887,928,929,930,931].
4. **Pragmatic trials of multicomponent threshold management programs, incorporating digital tools**. Comparisons of comprehensive threshold management vs. standard pharmacological optimization, incorporating biomarker outcomes alongside seizure frequency endpoints, would provide definitive evidence of clinical utility [897,899,901,903]. Validated threshold tracking infrastructure would simultaneously enable individual patient optimization and population-level observational research [911,914,916,917].
5. **Mechanistic studies of precipitant-pathophysiology interactions**. Translational studies measuring inflammatory, oxidative, and mitochondrial-health biomarkers before and after precipitant exposure would elucidate how acute precipitants interact with chronic distal vulnerabilities and identify additional intervention targets [14,41,847,851,852,854,856].
6. **Long-term outcomes and disease modification**. Longitudinal studies would establish whether interventions targeting distal mechanisms modify disease trajectory—slowing epilepsy progression, reducing development of pharmacoresistance, or improving long-term prognosis—rather than producing symptomatic management alone [887,892,932,933].

Translation of this framework into routine clinical practice faces multiple barriers. Time constraints in contemporary clinical practice limit feasibility of comprehensive precipitant assessment, dietary counseling, and behavioral intervention planning. Validated clinical decision support tools for mechanistic phenotyping and personalized intervention selection do not yet exist; and interventions such as KD require dietitian support that may not be readily accessible [596,897,899].

Despite these constraints, opportunities exist for incremental implementation. Systematic precipitant assessment can be incorporated into routine clinic visits through brief screening questionnaires, with seizure diaries capturing both seizure occurrence and potential contributing factors [14,41,909]. Selection of ASMs with favorable anti-inflammatory or AOX profiles when choosing among options with equivalent seizure-control efficacy represents a no-added-burden approach to incorporating mechanistic considerations.

Botanical interventions may offer particularly accessible entry points. Many herbal preparations are available without prescription, and patients often perceive them as more acceptable than additional pharmaceutical agents, although potential herb-drug interactions may limit polytherapy. Clinicians can proactively discuss evidence-based botanical options, recommend preparations with verified active compound content, and monitor for herb-drug interactions [737,738,841].

Digital health tools, once developed and validated, would substantially reduce the implementation burden: rather than requiring detailed precipitant review in every visit, apps would generate summaries of patient-identified patterns, and the same infrastructure would support the research agenda outlined in Section 3.2.3 [911,914,916].

Implementing this framework requires a conceptual reframing: ASMs remain essential, but within comprehensive threshold management, seizure suppression becomes a component in a coordinated system that also includes anti-inflammatory and AOX pharmacotherapy, dietary, behavioral, and botanical strategies—each addressing distinct vulnerability pathways. This is not a replacement for established treatment but a reconceptualization of what treatment is: coordinated optimization of the full excitability landscape rather than sequential pharmacological trials at its most distal point.

Critically, many of the most effective threshold modulators—sleep regulation, stress management, dietary consistency, precipitant avoidance—can only be implemented by patients themselves. Patient education about the mechanistic basis of threshold management, combined with standardized seizure, precipitant, and intervention-tracking tools, transforms patients from passive recipients of drug prescriptions into active partners in seizure control. Patients with DRE have the greatest stake in achieving seizure freedom. Supporting their understanding and agency is clinically essential. Digital tools, once validated, will further elevate this partnership to generate population-level data for research.

Although this review centers on DRE, where unmet need is greatest [887,934], the threshold management framework is not inherently restricted to this population. Patients on stable monotherapy with residual breakthrough seizures or significant side effects represent a natural extension. Beyond seizure outcomes, addressing the distal triad yields systemic benefits that extend beyond epilepsy management: interventions targeting chronic inflammation, oxidative stress, and mitochondrial dysfunction are expected to reduce cardiometabolic risk, support cognitive function, and improve metabolic health, all of which are meaningful gains for a population whose systemic health is already substantially burdened by the disease and its treatments.

## 4. Conclusions

Drug-resistant epilepsy remains a major unmet clinical problem. After failure of two appropriately chosen and tolerated antiseizure medications, the probability of sustained seizure freedom declines sharply, and many patients enter a prolonged course marked by recurrent seizures, cumulative morbidity, and reduced quality of life [9,10,935,936]. The present review argues that this clinical reality cannot be fully understood through proximal mechanisms alone. Neuroinflammation, oxidative stress, and mitochondrial dysfunction form a tightly interconnected distal pathophysiological triad that lowers seizure threshold, amplifies network instability, and may contribute to pharmacoresistance and disease progression [46,82,230,886,887]. In this framework, seizures are both a consequence and a driver of ongoing molecular injury, creating a self-reinforcing cycle that helps explain why established epilepsy can become progressively more difficult to control, and why interventions targeting only immediate seizure suppression may be insufficient. Addressing this cycle requires targeting proximal and distal mechanisms in tandem—not replacing antiseizure medication but embedding it within a broader strategy that also engages the pathways sustaining network injury, while leveraging patient agency.

The evidence reviewed supports the therapeutic relevance of this framework while underscoring its current limitations: several intervention classes can engage distal pathways in preclinical models and early clinical studies, but evidence strength varies substantially, and many promising approaches lack rigorous epilepsy-specific clinical validation [596,810,887,937,938,939]. Translating the framework into precision clinical practice will require standardized, interpretable biomarkers of distal dysfunction, mechanistic phenotyping to identify dominant drivers in individual patients, and trial designs suited to multicomponent, context-dependent interventions [185,890,892,897,899].

The immediate value of a threshold-based framework lies in organizing existing evidence, clarifying why adjunctive interventions targeting distal mechanisms may benefit some patients, and shifting clinical attention from repeated cycling through mechanistically similar drugs toward coordinated optimization of the full excitability landscape. Its longer-term promise is the development of biomarker-informed, personalized treatment strategies that reduce seizures, limit ongoing network injury, and modify disease trajectory. Realizing this promise requires not only research investment and clinical infrastructure, but a reconceptualization of the patient’s role: many of the most effective threshold modulators can only be implemented by patients themselves, and an engaged, informed patient is a prerequisite for comprehensive threshold management. For patients with DRE—and for the broader population living with epilepsy whose seizure control remains incomplete—this represents a meaningful expansion of what treatment can be. 

## Figures and Tables

**Table 1:** Representative review articles (2005–2026) analyzing the interplay between epileptic seizures, neuroinflammation, oxidative stress, and mitochondrial dysfunction.

Title	First Author (Year)	Journal	Key Focus	Ref.
**Seizures and inflammatory mediators**				
Brain inflammation in epilepsy: experimental and clinical evidence	Vezzani (2005)	Epilepsia	Early review proposing that brain inflammation is not only a consequence of seizures but also a contributor to epileptogenesis.	[51]
The role of inflammation in epilepsy	Vezzani (2011)	Nat. Rev. Neurol.	Reviews how proinflammatory cytokines and innate immune signaling promote seizures and epileptogenesis.	[45]
Inflammation and epilepsy: the foundations for a new therapeutic approach in epilepsy?	Walker (2012)	Epilepsy Curr.	Conceptual review arguing that immune modulation may offer new therapeutic avenues in epilepsy.	[53]
Epilepsy and brain inflammation	Vezzani (2013)	Exp. Neurol.	Summarizes glial-driven neuroinflammation in experimental and drug-resistant epilepsy and its therapeutic relevance.	[52]
Inflammatory mediators in human epilepsy: a systematic review and meta-analysis	de Vries (2016)	Neurosci. Biobehav. Rev.	Meta-analysis showing elevated proinflammatory cytokines and chemokines in human epilepsy.	[83]
The role of inflammation in the development of epilepsy	Rana (2018)	J. Neuroinflammation	Reviews central and peripheral inflammatory mechanisms, including BBB dysfunction, in epileptogenesis.	[84]
Role of innate immune receptor TLR4 and its endogenous ligands in epileptogenesis	Paudel (2020)	Pharmacol. Res.	Focuses on TLR4-related innate immune signaling as a driver and potential target in epileptogenesis.	[85]
Neuroinflammatory mechanisms of post-traumatic epilepsy	Mukherjee (2020)	Neurobiol. Dis.	Reviews how post-traumatic glial activation, cytokine upregulation, and BBB disruption promote epileptogenesis.	[86]
The role of neuroinflammation in post-traumatic epilepsy	Sun (2021)	Front. Neurol.	Summarizes neuroinflammatory mechanisms linking traumatic brain injury to post-traumatic epilepsy.	[87]
Novel approaches to prevent epileptogenesis after traumatic brain injury	Dulla (2021)	Neurotherapeutics	Reviews anti-inflammatory and antioxidant strategies to prevent epileptogenesis after traumatic brain injury.	[88]
Neuroinflammatory mediators in acquired epilepsy: an update	Chen (2023)	Inflammation Res.	Updates inflammatory mediators implicated in acquired epilepsy as biomarkers and therapeutic targets.	[44]
Neuroinflammation in epileptogenesis: from pathophysiology to therapeutic strategies	Li (2023)	Front. Immunol.	Reviews microglial, astrocytic, and peripheral immune mechanisms in epileptogenesis and anti-inflammatory targets.	[55]
Neuroinflammation and status epilepticus: a narrative review unraveling a complex interplay	Foiadelli (2023)	Front. Pediatr.	Reviews neuroinflammatory mechanisms and cytokine-targeted therapies in status epilepticus.	[54]
Inflammasomes at the crossroads of traumatic brain injury and post-traumatic epilepsy	Javalgekar (2024)	J. Neuroinflammation	Highlights inflammasome signaling as a mechanistic link between TBI and post-traumatic epilepsy.	[89]
Neuroinflammation and epilepsy: from pathophysiology to therapies based on repurposing drugs	Sanz (2024)	Int. J. Mol. Sci.	Reviews neuroinflammatory pathways in epilepsy and drug-repurposing approaches targeting them.	[56]
Modulation of neuroinflammation as a therapeutic strategy for the control of epilepsy	Ramos (2025)	Seizure	Therapeutically focused review of inflammatory pathways, biomarkers, and DRE-relevant targets in epilepsy.	[57]
Role of neuroinflammation factors as potential biomarkers of epilepsy: a narrative review	Zawadzka (2025)	Neurologia i Neurochirurgia Polska	Reviews neuroinflammatory mediators as candidate biomarkers of epilepsy activity and treatment response.	[90]
**Seizures and glia**				
Astrocyte immune responses in epilepsy	Aronica (2012)	Glia	Reviews reactive astrocytes as key inflammatory and homeostatic regulators in epilepsy.	[58]
Microglia–neuron communication in epilepsy	Eyo (2017)	Glia	Summarizes microglia-neuron communication in seizures and the dual roles of microglia in epilepsy.	[59]
Microglia in epilepsy	Yu (2023)	Neurobiol. Dis.	Comprehensive review of microglial phenotypes and functions, including links to drug resistance.	[60]
Astrocytes as a target for therapeutic strategies in epilepsy: current insights	Çarçak (2023)	Front. Mol. Neurosci.	Reviews astrocytic dysfunction in epilepsy and emerging astrocyte-targeted therapies.	[61]
The role of glial cells in the pathophysiology of epilepsy	Onat (2025)	Cells	Integrative review of astrocytic and microglial contributions to network hyperexcitability in epilepsy.	[62]
Metabolic reprogramming of astrocytes and microglia as a driver and therapeutic target in epileptogenesis	Zakharova (2026)	Biochemistry (Mosc.)	Reviews astrocyte and microglial metabolic reprogramming as a contributor to epileptogenesis and as a potential therapeutic target.	[91]
**Seizures, oxidative stress, and mitochondrial dysfunction**				
Oxidative stress and epilepsy: literature review	Aguiar (2012)	Oxid. Med. Cell. Longev.	Reviews oxidative stress and mitochondrial dysfunction in epilepsy and the potential of antioxidants.	[63]
Mitochondrial involvement and oxidative stress in temporal lobe epilepsy	Rowley (2013)	Free Radic. Biol. Med.	Summarizes evidence that mitochondrial impairment and oxidative damage contribute to temporal lobe epilepsy.	[64]
Oxidative stress associated with neuronal apoptosis in experimental models of epilepsy	Méndez-Armenta (2014)	Oxid. Med. Cell. Longev.	Reviews oxidative stress, mitochondrial dysfunction, and apoptosis in experimental epilepsy.	[65]
Metabolic dysfunction and oxidative stress in epilepsy	Pearson-Smith (2017)	Int. J. Mol. Sci.	Reviews metabolic dysfunction and redox imbalance as both causes and consequences of epilepsy.	[46]
Role of oxidative stress in epileptogenesis and potential implications for therapy	Borowicz-Reutt (2020)	Pharmacol. Rep.	Focuses on oxidative stress in epileptogenesis and antioxidant adjunctive therapy.	[66]
The oxidative stress in epilepsy-focus on melatonin	Kamieniak (2024)	Int. J. Mol. Sci.	Reviews oxidative stress in epilepsy with emphasis on melatonin as an antioxidant anticonvulsant.	[67]
Mitochondria and oxidative stress in epilepsy: advances in antioxidant therapy	Ji (2024)	Front. Pharmacol.	Summarizes mitochondrial dysfunction and antioxidant therapeutic strategies in epilepsy.	[68]
Mitochondrial dysfunction in epilepsy: mechanistic insights and clinical strategies	Zhang (2025)	Mol. Biol. Rep.	Mechanistic and clinically oriented review of mitochondrial dysfunction in epilepsy.	[69]
Oxidative stress and neuronal alteration: Mitochondrial dysfunction as a key player in intractable epilepsy—a narrative review	Raza et al. (2026)	Pathol. Res. Pract.	Narrative review of mitochondrial dysfunction and oxidative injury in intractable epilepsy.	[70]
**Interaction between inflammation and oxidative stress in epilepsy**				
Inflammation and reactive oxygen species in status epilepticus: biomarkers and implications for therapy	Terrone (2019)	Epilepsy Behav.	Reviews coupled inflammatory and oxidative responses in status epilepticus and their biomarker value.	[77]
Modulating neuroinflammation and oxidative stress to prevent epilepsy and improve outcomes after traumatic brain injury	Eastman (2020)	Neuropharmacology	Reviews combined anti-inflammatory and antioxidant strategies to prevent epilepsy after TBI.	[47]
Crosstalk between neuroinflammation and oxidative stress in epilepsy	Fabisiak (2022)	Front. Cell Dev. Biol.	Summarizes crosstalk between neuroinflammation and oxidative stress in epilepsy.	[79]
The interconnected mechanisms of oxidative stress and neuroinflammation in epilepsy	Parsons (2022)	Antioxidants	Reviews oxidative stress-neuroinflammation interactions and their impact on synaptic dysfunction in epilepsy.	[78]
The Keap1/Nrf2/ARE/HO-1 axis in epilepsy: crosstalk between oxidative stress and neuroinflammation	Manavi (2025)	Int. Immunopharmacol.	Focuses on the Nrf2/Keap1/HO-1 axis as a bridge between oxidative stress and neuroinflammation in epilepsy.	[80]
**Seizures and blood–brain barrier (BBB) dysfunction**				
Blood–brain barrier dysfunction, seizures and epilepsy	van Vliet (2015)	Semin. Cell Dev. Biol.	Core review on bidirectional interactions between BBB dysfunction, seizures, and epilepsy.	[71]
Role of blood–brain barrier dysfunction in the development of poststroke epilepsy	Meijer (2024)	Epilepsia	Reviews BBB disruption as an epileptogenic mechanism in post-stroke epilepsy.	[73]
Unveiling the hidden connection: the blood–brain barrier’s role in epilepsy	Han (2024)	Front. Neurol.	Broad narrative review of BBB breakdown and its translational relevance in epilepsy.	[74]
Evidence implicating blood–brain barrier impairment in the development of acquired epilepsy	Bernardino (2024)	J. Pharmacol. Exp. Ther.	Reviews experimental and translational evidence that BBB impairment contributes to acquired epilepsy.	[72]
The role of blood–brain barrier disruption in epilepsy	Suleymanova (2025)	Neurol. Int.	Mechanism-oriented review of BBB leakage and downstream inflammatory signaling in epilepsy progression.	[76]
Blood–brain barrier dysfunction in epilepsy	Huang (2025)	Int. J. Mol. Med.	Reviews mechanisms and therapeutic implications of BBB dysfunction in epilepsy.	[75]
**Integrative and translational perspectives**				
Therapeutic strategies to ameliorate neuronal damage in epilepsy by regulating oxidative stress, mitochondrial dysfunction, and neuroinflammation	Madireddy (2023)	Brain Sci.	Integrative review of major pathogenic mechanisms and therapeutic approaches in epilepsy, from neuroinflammation and oxidative stress to ASMs/AEDs, diet, neuromodulation, and surgery.	[82]
Epileptogenesis and epilepsy treatment: advances in mechanistic understanding, therapeutic approaches, and future perspectives	Mazhit (2026)	Int. J. Mol. Sci.	Integrative review linking epileptogenic mechanisms with pharmacological, surgical, and neuromodulatory treatment strategies.	[81]

## Data Availability

No new data were created or analyzed in this study. Data sharing is not applicable to this article.

## Supplementary Materials

The following supporting information can be downloaded at: https://www.mdpi.com/article/10.3390/ijms27156606/s1.

## Abbreviations

The following abbreviations are used in this manuscript:

4-HNE	4-hydroxynonenal
5-HT	5-hydroxytryptamine, serotonin
AED	antiepileptic drug
AID	anti-inflammatory drug
AMPA	α-amino-3-hydroxy-5-methyl-4-isoxazolepropionic acid
AMPAR	AMPA receptor
AOX	antioxidant
ASM	antiseizure medication
ATP	adenosine triphosphate
BBB	blood–brain barrier
BDNF	brain-derived neurotrophic factor
BHB	β-hydroxybutyrate
CD	cluster of differentiation
CNS	central nervous system
CoQ10	coenzyme Q10
COX-2	cyclooxygenase-2
CYP	cytochrome P450
DHA	docosahexaenoic acid
DRE	drug-resistant epilepsy
EEG	electroencephalography
EPA	eicosapentaenoic acid
FIRES	febrile infection-related epilepsy syndrome
FSP1	ferroptosis suppressor protein 1
GABA	gamma-aminobutyric acid
GABA_A_R	GABA receptor type A
GPx	glutathione peroxidase
GSH	reduced glutathione
GSSG	oxidized glutathione
HDAC	histone deacetylase
HIF-1α	hypoxia-inducible factor-1 alpha
HMGB1	high mobility group box 1
HO-1	heme oxygenase-1
Iba1	ionized calcium-binding adapter molecule 1
IL	interleukin
iNOS	inducible nitric oxide synthase
KA	kainic acid
KD	ketogenic diet
Keap1	Kelch-like ECH-associated protein 1
LDH	lactate dehydrogenase
LOC	locus of control
LPO	lipid peroxidation
LPS	lipopolysaccharide
MAD	modified Atkins diet
MDA	malondialdehyde
mPTP	mitochondrial permeability transition pore
NAD	nicotinamide adenine dinucleotide
NADPH	nicotinamide adenine dinucleotide phosphate, reduced form
NQO1	NAD(P)H quinone dehydrogenase 1
NF-κB	nuclear factor kappa B
NLRP3	NLR family pyrin domain-containing protein 3
NMDA	N-methyl-D-aspartate
NMDAR	NMDA receptor
NO	nitric oxide
NOS	NO synthase
NOX	NADPH oxidase
NR2A/2B	NMDAR subunit 2A/2B
Nrf2	nuclear factor erythroid 2-related factor 2
NSAID	nonsteroidal anti-inflammatory drug
OS	oxidative stress
OTC	over-the-counter drug
PGC-1α	peroxisome proliferator-activated receptor gamma coactivator 1-alpha
PGE_2_	prostaglandin E_2_
POLG	polymerase gamma
PTZ	pentylenetetrazole
RCT	randomized controlled trial
RNS	reactive nitrogen species
ROS	reactive oxygen species
SE	status epilepticus
Sirt3	sirtuin 3
SOD	superoxide dismutase
SV2A	synaptic vesicle glycoprotein 2A
TBI	traumatic brain injury
TGF-β	transforming growth factor beta
TLE	temporal lobe epilepsy
TLR4	Toll-like receptor 4
TMEV	Theiler’s murine encephalomyelitis virus
TNF	tumor necrosis factor
VPA	valproate/valproic acid
ΔΨm	mitochondrial membrane potential
