# Supplementary material for: Anti-Inflammatory and Antioxidant Strategies in Epilepsy: From Molecular Mechanisms to Threshold Management
Source: Int J Mol Sci. 2026 Jul 24;27(15):6606. doi: 10.3390/ijms27156606 (PMC13466846; doi:10.3390/ijms27156606)
Supplement: Supplementary file 1 [file ijms-27-06606-s001.zip › ijms-4272074-supplementary.pdf]

Supplementary Table S1. Anti- and pro-inflammatory and anti- and pro-oxidative / mitochondrial profiles of major antiseizure medications (ASMs).

ASM, mechanism / main anti-seizure action	Ref	Model / clinical condition	Anti- and pro-inflammatory effects	Anti- and pro-oxidative / mitochondrial effects
**Carbamazepine (CBZ)** **Na^+ channel blocker**	[1]	**rat infl models**: brewer's yeast / carrageenan	**Anti:** ↓infl hyperalgesia, ↓paw edema, ↓infl exudate, ↓PGE_2-like activity, ↓substance P in exudate.	-
Stabilizes axonal membranes; prevents repetitive firing.	[2]	**cell culture:** primary rat glia + LPS	**Anti:** dose-dependent ↓NO and ↓PGE_2 in LPS-stimulated glia (up to 77% and 88%, respectively).	-
Reduces seizure spread in focal epilepsy.	[3]	**cell culture:** microglial activation model	**Anti:** selective ↓LPS-induced microglial iNOS via ↓Akt activation.	-
	[4]	**rat hippocampus**: basal + LPS + pro-convulsant conditions	**Anti:** ↓hippocampal IL-1β / TNF under basal and LPS conditions; CBZ > VPA for anti-cytok effect.	-
	[5]	**cell culture:** primary astrocytes + IFNγ / TNF	**Anti:** ↓cytok-induced astroglial L-glutamate release via A_{2A}-linked signaling.	-
	[6]	**clinical, epilepsy pts**: CBZ + vitB12, 3 mo	**Anti:** ↓serum TNF, ↓hsCRP (high-sensitivity C-reactive protein); parallel ↓sz freq / duration. **Pro:** combo-tx only; Hcy risk ↑/persisted.	-
	[7]	**clinical genetics**: CBZ in pts with HLA-A*31:01 (human leukocyte antigen A*31:01 allele)	**Pro:** immune hypersensitivity context; HLA-A*31:01-linked SCAR risk: DRESS / SJS / TEN.	-
	[8]	**mouse:** CBZ liver-injury model	**Pro:** liver-injury infl context: ↑TLR4 / RAGE-related infl signaling.	**Pro:** ↓hepatic GSH, altered OS markers.
	[9]	**clinical**: epileptic children on CBZ	-	**Pro:** ↓TAC, ↑TOS, ↑OSI; plasma CBZ correlates with oxidant status.

	[10]	**clinical**: adult epilepsy pts, CBZ vs VPA monotherapy	-	**Pro:** ↓serum PON1 (paraoxonase 1), ↓AChE (acetylcholinesterase) with CBZ vs VPA.
	[11]	**clinical**: epilepsy monotherapy redox study	-	**Pro:** ↑nitrite levels, ↓SOD with CBZ monotherapy.
	[12]	**rat:** chronic CBZ hepatotoxicity ± NAC	-	**Pro:** ↑TBARS, ↑AST / ALT, ↓GSH, ↓SOD / GPx / CAT; NAC reversed redox + histol damage.
	[13]	**cell culture:** brain microvascular endothelial cells / BBB	-	**Pro:** ↓mito complex activity, ↓OCR, ↑lactate, barrier dysfunction; bioenergetic disruption / mito toxicity.
	[14]	**cell culture:** freshly isolated rat hepatocytes	-	**Pro:** ↑ROS, ↑LPO, ↓ΔΨm, ↓GSH, ↑GSSG; taurine / melatonin attenuate injury.
**Phenytoin (PHT)**<br>**Na^+ channel blocker**<br><br>Limits repetitive firing by prolonging Na^+ channel inactivation.<br><br>Effective for focal and generalized tonic-clonic seizures.	[15]	**clinical, meta-analysis:** epilepsy pts, PHT monotherapy	**Pro:** ↑Hcy, ↓folate, ↓vitB12 → systemic vascular-risk / low-grade infl background.	-
	[16]	**clinical, switch study**: epilepsy pts switched from PHT/CBZ to LEV/LTG	**Pro:** off PHT/CBZ → ↓CRP; off PHT specifically → ↓Hcy.	-
	[17]	**clinical, case report**: PHT hypersensitivity syndrome	**Pro:** DRESS / SJS / TEN overlap; idiosyncratic peripheral immune hypersensitivity, not chronic CNS pro-inf.	-
	[18]	**rat:** pilocarpine szs + PHT ± CoQ10	-	**Pro:** PHT-associated cognitive deficit + OS; CoQ10 attenuates OS / behavioral deficit.
	[19]	**cell culture:** freshly isolated rat hepatocytes	-	**Pro:** ↑ROS, ↑LPO, ↓GSH, ↑GSSG, ↓ΔΨm, mito swelling, ↓viability; taurine / melatonin protective.
	[20]	**clinical, cross-sectional**: old vs new ASM groups	-	**Pro:** old ASM group incl PHT → ↑protein carbonyl burden / less favorable redox balance vs newer ASMs.

**Oxcarbazepine (OXC) / Eslicarbazepine (ESL)**<br>**Na^+ channel blockers**<br>Block voltage-gated Na^+ channels; eslicarbazepine is the active metabolite.<br>Used mainly in focal seizures.	[21]	**clinical:** children with idiopathic epilepsy, OXC monotherapy, 3-6 mo	-	**Anti:** ↓serum NO, ↓MDA after OXC tx.
	[22]	**clinical:** epileptic pts on OXC monotherapy, 1 yr	-	**Anti:** erythrocyte SOD / GPx significantly changed after OXC tx; MDA significantly different vs pretreatment and controls.
	[23]	**mouse:** PTZ kindling	-	**Anti:** no significant alteration in OS parameters with OXC; unlike TPM, no ↑MDA / ↓GSH.
**Lamotrigine (LTG)**<br>**Na^+ channel blocker; glutamate release inhibitor**<br>Stabilizes Na^+ channels; inhibits glutamate/aspartate release.<br>Broad-spectrum for focal and generalized seizures.	[24]	**cell culture:** astrocyte-microglia co-culture ± infl conditions	**Anti:** ↑TGF-β1 without ↑TNF; ↓overall infl activity; mild ↓glial viability at higher conc.	-
	[25]	**clinical:** epilepsy pts with depressive symptoms	**Anti:** ↓serum TNF; anti-peripheral infl signal with improved depressive symptoms.	-
	[26]	**rat:** PTZ kindling	-	**Anti:** LTG alone did not ↑OS markers; attenuated PTZ-related OS vs kindled condition / vs CBZ-treated kindled rats.
	[27]	**rat:** PTZ kindling	-	**Anti:** ↓MDA; improved vascular-risk markers; ↓hippocampal damage.
	[28]	**rat:** chronic mild stress	-	**Anti:** ↑SOD / CAT and ↓protein carbonyls.<br>**Pro:** region-dependent ↑MDA.
	[29]	**rat:** experimental depression	-	**Anti:** ↓LPO; ↑GSH-Px, ↑GSH, ↑vitC in cortex.
	[20]	**clinical, cross-sectional:** old vs new ASM groups	-	**Anti:** newer ASM group incl LTG → more favorable redox profile vs old ASMs.
**Zonisamide (ZNS)**	[30]	**mouse:** MPTP PD model	**Anti:** ↓microglial Nav1.6, ↓Iba1, ↓TNF, ↓$gp91^{phox}$ → dampened neuroinfl signaling.	-

**Na^+ / T-type Ca^{2+} blocker**<br><br>Broad-spectrum: inhibits Na^+ and T-type Ca^{2+} channels; weak carbonic anhydrase inhibition.<br><br>Used in focal and generalized seizures.	[31]	**cell-free:** ESR (electron spin resonance) radical assay	-	**Anti:** dose-dependent scavenging of •OH and NO•.
	[32]	**rat:** ferric chloride brain injury	-	**Anti:** prevented ↑8-OHdG in cerebrum → ↓oxidative DNA injury.
	[33]	**cell culture:** PC12 cells + MPP^+	-	**Anti:** attenuated oxidative toxicity; modulated Ca^{2+} influx and ↓caspase-3 activity; also reported ↓LPO / ↑GSH / ↑GSH-Px.
	[34]	**cell culture / mouse:** astrocytes + 6-OHDA PD model	-	**Anti:** ↑GSH and GSH synthesis-related molecules in astrocyte-targeted neuroprotection context.
	[35]	**cell culture / rat:** astrocytes + 6-OHDA PD model	-	**Anti:** ↑astrocytic anti-oxidative factor mRNAs incl Cu/Zn-SOD and MnSOD.
**Lacosamide (LCM)**<br><br>**Na^+ slow-inactivation enhancer**<br><br>Enhances slow Na^+ channel inactivation; reduces repetitive firing without affecting physiological fast firing.<br><br>Adjunct for focal seizures.	[36]	**rat:** pilocarpine SE, 24 h	**Anti:** weak ↓IL-1β; no clear TNF change.	**Anti:** ↓MDA, normalized CAT; high-dose LCM restored SOD toward ctrl.
	[37]	**rat:** chronic post-SE TLE / epileptogenesis	**Anti:** ↓hipp IL-1β / TNF; ↓neuronal damage; ↓spont motor szs.	**Anti:** ↑SOD, ↑GSH, ↓CAT, ↓LPO in hipp.
	[38]	**mouse:** LPS- and LPS+PILO-induced neuroinf-mediated szs	**Anti:** ↓infl cytokines; ↓hipp kynurenine.	**Anti:** ↓OS markers in LPS and LPS+PILO groups.
	[39]	**mouse:** pilocarpine SE	**Pro:** very high-dose LCM showed inverted-U response with tendency to worse SE / OS outcome at highest dose.	**Pro:** dose-dependent caution for OS; not a consistent pro-OS signal at lower doses.
**Diazepam (DZP)**<br><br>**Benzodiazepine, $GABA_A$ positive allosteric modulator**	[40]	**rat:** carrageenan paw edema / pleurisy / vascular permeability	**Anti:** ↓paw edema, ↓pleural exudate, ↓leukocyte count, ↓vascular permeability; corticosterone-linked anti-infl effect.	-

Binds benzodiazepine site on $GABA_A$ receptor → ↑Cl^- influx (↑channel opening frequency). Stops ongoing seizures, including status epilepticus, and provides short-term seizure prophylaxis.	[41]	**cell culture / mouse:** macrophages / DCs (dendritic cells) + LPS; LPS-septic shock; EAE (experimental autoimmune encephalomyelitis)	**Anti:** macrophages / DCs → ↓IL-12, ↓TNF, ↓IL-6, ↓NO, ↓CD40; septic shock → ↓death, ↑IL-10-shift; EAE → ↓clinical severity / infl readouts.	-
	[42]	**rat:** carrageenan edema after acute vs 21-day DZP	**Anti:** acute DZP ↓CIPE and ↑corticosterone; chronic DZP lost anti-infl effect → tolerance of anti-infl / corticosterone response.	-
	[43]	**rat:** acute restraint stress	-	**Anti:** ↓striatal LPO, mild ↑SOD activity, ↑mito function.
	[44]	**mouse:** LPS + DZP and/or swimming exercise	-	**Anti:** DZP alone no clear AOX effect; DZP+exercise ↓MDA / GSSG, ↑GSH in PFC > either alone → combo-dependent AOX benefit.
**Phenobarbital (PB)** **Barbiturate, $GABA_A$ agonist**	[45]	**clinical:** epilepsy outpatients on stable AED tx	**Pro:** PB grouped with enzyme-inducing AEDs; ↓folate / vitB6, ↑Hcy → systemic vascular-risk / low-grade infl background.	-
Prolongs $GABA_A$ channel openings; at higher doses can directly open Cl^- channel. Controls generalized and neonatal seizures.	[46]	**clinical:** adults with epilepsy; PB / PHT / PRD (pyridoxine-dependent epilepsy) group vs controls	**Pro:** PB-containing inducer group → ↑p-tHcy (post-load total homocysteine), ↓serum and erythrocyte folate.	-
	[47]	**clinical:** large prospective epilepsy cohort on AEDs	**Pro:** PB associated with ↓vitB12; low folate / vitB12 associated with ↑Hcy.	-
	[48]	**clinical:** epileptic children on PB monotherapy	-	**Pro:** ↑serum lipid hydroperoxides, ↑OSI vs controls; PB redox profile less favorable than VPA.
**Tiagabine (TGB)** **GABA reuptake inhibitor**	[49]	**cell culture / mouse:** BV-2 microglia + LPS / MPP^+; MPTP PD model	**Anti:** ↓microgl activation; BV-2 cells: ↓NF-κB nuclear translocation, ↓infl mediators; in vivo ↓nigral microgliosis, partial nigrostriatal protection.	-

Inhibits GAT-1 GABA transporter, increasing synaptic GABA levels and prolonging inhibitory post-synaptic potentials.<br><br>Adjunct for focal seizures.	[50]	**rat:** focal cerebral ischemia / MCAO-reperfusion	**Anti:** ↓infarct vol, improved neurobehavioral outcome; neuroprotection compatible with ↓secondary neuroinfl.	-
	[51]	**rat:** focal embolic cerebral ischemia	**Anti:** ↓brain infarct vol after post-embolization TGB; indirect anti-infl relevance.	-
	[52]	**mouse:** PTZ kindling	**Anti:** ↓neuroinfl markers expression; improved sz, behavioral and cognitive outcomes.	**Anti:** ↓brain OS; study conclusion links benefit to reduced OS + BDNF/TrkB modulation.
	[53]	**cell culture:** primary cortical astrocytes + OS	-	**Anti:** astrocyte protection vs OS; ROS / iNOS / HSP-related stress readouts improved.
**Vigabatrin (VGB)**<br><br>**GABA transaminase inhibitor**	[54]	**rat:** carrageenan granuloma / edema	**Anti:** in vivo anti-infl activity in carrageenan-type models; ↓granuloma / edema-related infl burden.	-
Irreversibly inhibits GABA-T, raising brain GABA levels.<br><br>Used for infantile spasms and refractory focal epilepsy.	[55]	**rabbit:** spinal ischemia-reperfusion	**Anti:** ↓secondary tissue injury / edema in acute ischemia-reperfusion setting.	**Anti:** ↓oxidative injury markers, improved AOX status, less tissue damage; strongest AOX signal in acute injury model.
	[56]	**clinical:** children with infantile spasms on VGB	**Pro:** retinal toxicity / visual-field injury risk rises with duration; retina-specific chronic toxicity, not global brain pro-infl.	-
	[57]	**rat:** VGB enantiomers under high-light retinal-toxicity conditions	-	**Pro:** retinal and systemic toxicity linked to the S-enantiomer; retina-specific toxic liability, not therapeutic anti-AOX signal.
	[58]	**mouse:** VGB retinal toxicity	**Pro:** retinal toxicity enhanced by phototransduction / light exposure; retina-specific degenerative injury.	-

**Valproate (VPA)**<br>**Multi-mechanism: ↑GABA, Na^+/Ca^{2+} block, HDAC inhibition**<br>Increases GABA levels, blocks Na^+ channels, modulates T-type Ca^{2+} currents.<br>Broad efficacy across many seizure types.	[4]	**rat hippocampus:** basal / LPS / pro-convulsant conditions	**No effect.** (CBZ and vinpocetine, but not VPA, ↓IL-1β / TNF expression)	-
	[59]	**clinical:** children with epilepsy; VPA monotherapy, 16 wk	**Anti:** ↓serum CCL2; no change in IL-1β.	-
	[48]	**clinical:** children with epilepsy; VPA monotherapy vs CBZ / PB / untreated	-	**Anti:** improved oxidant / antioxidant balance vs CBZ / PB groups; no change total peroxide / OSI signal vs controls.<br>**Pro:** ↓uric acid vs untreated group.
	[11]	**clinical:** monotherapy redox study; VPA vs LTG / CBZ / controls	-	**Pro:** in VPA group, only ↓CAT activity observed; discrete redox shift, not strong AOX effect.
	[60]	**clinical:** children with epilepsy; 1 yr VPA	-	**Pro:** obese VPA-treated subgroup → ↓vitE, ↑MDA, ↑lag phase vs non-obese VPA pts / ctrls; context-dependent pro-OS with metabolic vulnerability.
**Ganaxolone (GNX) / allopregnanolone (ALLO)**<br>**Neurosteroid $GABA_A$ positive allosteric modulator, with relevance to extrasynaptic δ-containing receptors**<br>GNX: synthetic ALLO analog; ↑tonic GABAergic inhibition; approved for seizures assoc with CDKL5 deficiency disorder.	[61]	**neonatal lamb:** GNX, perinatal asphyxia / neonatal sz	**Anti:** ↓SE sz number, ↓total sz dur; ↓neuronal degeneration in hippocampal CA1 / CA3 + cortex; ↓thalamic neuroinfl.	-
	[62]	**ovariectomized rat:** GNX, lysolecithin focal demyelination, corpus callosum	**Anti:** ↑microgl myelin-debris clearance, ↑remyelination / myelin proteins; no clear change in IL-1β, TNF, IL-6, IL-4, IL-10, astrocytosis.	-
	[63]	**cell culture / mouse:** GNX, APP (amyloid precursor protein)-transfected SH-SY5Y cells; Aβ1-42 AD model	**Anti:** ↓TNF, ↓IL-1β, ↓Aβ toxicity / tau-linked changes, ↓neuronal apoptosis.	**Anti:** ↓mROS (mitochondrial ROS), restored ΔΨm / mito fx.

	[64]	**cell culture / mouse:** ALLO, RAW264.7 macrophages + LPS; brain / immune models	**Anti:** ↓TLR4-dep pro-infl signaling; ↓TRAF6 / p-TAK1 / NF-κB / pCREB / HMGB1 / MCP-1 (monocyte chemoattractant protein 1) / TNF.	-
	[65]	**cell culture:** ALLO, astrocytes	**Anti:** ↓inflammasome indicators, ↓apoptotic signaling.	**Anti:** ↑ATP generation, ↑mito fx / dynamics, ↓OS.
**Felbamate (FBM)**<br>**NMDA receptor antagonist; GABA$_A$ modulator**	[66]	**rat:** TBI; FBM and LEV arms	**Anti:** FBM group, ↓IL-1β / IL-4 / IL-6 / TNF; ↓vascular congestion / mononuclear infiltr / hemorrhage / neuronal degen; ↓Purkinje cell degen.	**Anti:** FBM group, ↓TBARS, ↑GSH, ↑SOD; combo > FBM alone.
Blocks NMDA receptors with relative NR2B preference; also modulates GABA$_A$ and high-frequency excitatory firing.<br>Effective in Lennox-Gastaut syndrome and refractory focal / generalized seizures.	[67]	**gerbil:** global ischemia / delayed CA1 apoptosis	**Anti:** ↓delayed CA1 neuronal apoptosis / neuron loss; post-hoc neuroprotection.	-
	[68]	**gerbil:** transient forebrain ischemia, 7- and 28-day study	**Anti:** ↓ischemic histol damage in cortex, hippocampal CA1 / CA4, thalamus, striatum.	-
	[69]	**mechanistic toxicology / metabolism**	-	**Pro:** atropaldehyde formed in vivo; glutathione conjugation / mercapturate pathway identified; toxic-metabolite relevance.
**Topiramate (TPM)**<br>**AMPA/kainate receptor antagonist; GABA$_A$ modulator**	[70]	**cell culture:** primary rat microglia + LPS	**Anti:** ↓IL-1β, ↓IL-6; at high conc. also ↓TNF; ↓LPS-induced microgl mito metabolic activation.	-
Multiple: antagonizes AMPA/kainate receptors, blocks voltage-gated Na^+ channels, enhances GABA$_A$ currents, and weakly inhibits carbonic anhydrase.	[71]	**rat:** PTZ-induced blood OS ± selenium	-	**Anti:** ↓plasma / erythrocyte LPO; ↑GSH-Px, ↑GSH; ↑vitC / vitE in selenium-containing groups; improved blood AOX redox readouts.
	[36]	**rat:** pilocarpine SE, 24 h	**Anti:** weak anti-infl, modest ↓hipp IL-1β; TNF unchanged.	**Anti:** normalized CAT and MDA; high-dose TPM restored SOD toward ctrl.

Broad-spectrum ASM for focal and generalized seizures.	[72]	**rat:** chronic methylphenidate, amygdala	**Anti:** ↓TNF, ↓IL-1β, ↓NF-κB; improved histol / neurobehavioral profile.	**Anti:** ↓LPO, ↓mito GSSG; ↑SOD / GPx / GR.
	[73]	**cell culture:** primary rat astrocytes	-	**Pro:** induced astrocytic stress / poorer tolerance vs GBP / LTG / TGB / LEV; ROS / MDA / iNOS / HSP70-related OS readouts assessed.
	[74]	**rat:** cocaine exposure	**Anti:** indirect anti-infl relevance via prevention of cocaine-induced NF-κB changes in frontal ctx.	**Anti:** prevented ↓hipp GSH / GPx and ↑hipp nNOS; prevented cocaine-induced OS / nitrosative changes.
	[75]	**cell culture:** PC12 cells + H_2O_2	-	**Anti:** modulated LPO and GSH / vitC-related AOX status in oxidant challenge model.
**Perampanel (PER)**<br>**AMPA receptor antagonist**	[76]	**rat:** TBI, acute	**Anti:** ↓TNF, ↓IL-1β; ↑IL-10, ↑TGF-β1.	**Anti:** ↓MDA, ↓4-HNE; preserved SOD / CAT / GST (glutathione S-transferase).
Selective non-competitive antagonist of AMPAR on postsynaptic neurons.	[77]	**cell culture / mouse:** HT22 + hemin; intracerebral hemorrhage (ICH)	**Anti:** ↓IL-1β, ↓IL-6, ↓TNF, ↓NF-κB; anti-necroptotic signal.	-
Prevents fast excitatory synaptic transmission.	[78]	**rat:** focal cerebral ischemia / MCAO	**Anti:** ↓IL-1β, ↓TNF; ↑IL-10, ↑TGF-β1.	**Anti:** ↓iNOS, ↓nNOS, ↓NO.
Adjunct for focal-onset and primary generalized tonic-clonic seizures.	[79]	**cell culture / rat:** triple-cell NVU + TNI (temporal neocortical index) / glutamate; TBI	**Anti:** ↓IL-1β, ↓IL-6; ↓microglial activation; preserved BBB / NVU.	**Anti:** ↓MDA, ↓4-HNE; ↑Sirt3, ↑IDH2 (isocitrate dehydrogenase 2) / SOD2.
	[80]	**neonatal rat:** photothrombotic perinatal stroke; PER-loaded PLGA (poly(lactic-co-glycolic acid))	**Anti:** ↓TNF, ↓IL-1β, ↓IL-6, ↓COX-2, ↓iNOS; ↑Ym1 (chitinase-like protein 3), ↑Arg1.	-
	[81]	**rat:** subarachnoid hemorrhage (SAH), 24 h	**Anti:** ↓IL-1β, ↓IL-18; ↓NLRP3 / ASC / cleaved caspase-1 / GSDMD-N (N-terminal gasdermin D); anti-infl / anti-pyroptotic signal.	**Anti:** ↓ROS, ↓MDA; ↑SOD, ↑GSH-Px; SIRT3 / FOXO3α-linked AOX effect.

	[82]	**mouse:** subarachnoid hemorrhage (SAH)	**Anti:** ↓IL-1β, ↓IL-6; anti-apoptotic signal.	-
	[83]	**cell culture / rat:** MCAO; primary cortical neurons + OGD	**Anti:** ↓microglial / astrocytic activation; anti-neuroinfl after ischemia.	**Anti:** ↓ROS, ↓4-HNE, ↓iron; ↑FTH-1 (ferritin heavy chain 1), ↑GPX-4, ↑FSP1; anti-ferroptotic / AOX signal.
**Levetiracetam (LEV)**<br><br>**SV2A modulator**<br><br>Binds synaptic vesicle protein 2A on presynaptic terminals, modulating Ca^{2+}-dependent vesicle exocytosis and dampening hypersynchronous glutamate and GABA release.<br><br>Broad-spectrum ASM used in focal and generalized seizures.	[84]	**rat:** chronic epilepsy, hippocampus + piriform ctx	**Anti:** ↓reactive astrogl / microgl; ↓IL-1β, ↓IL-1R1 in hippocampus and piriform ctx.	-
	[85]	**cell culture:** primary rat astrocyte-microglia co-cultures; infl M30 state / IL-1β challenge	**Anti:** ↑TGFβ1 in dose-dependent manner; restored astrogl membrane resting potentials / inward and outward rectifier currents in infl co-cultures.	-
	[86]	**mouse:** pilocarpine SE, acute epileptogenesis	**Anti:** ↓BBB leakage; ↓$CD31^{+}$ immature endothelial cells / angiogenic factors; ↓pro-infl responses in hippocampal microgl / astrogl.	-
	[87]	**cell culture / mouse:** BV-2 microglia; pilocarpine SE / epileptogenesis	**Anti:** ↓microgl activation / phagocytosis; ↓cytokine expression during epileptogenesis; stronger anti-neuroinfl effect than VPA / CBZ in this study.	-
	[88]	**cell culture / mouse:** BV-2 + LPS; pilocarpine SE	**Anti:** ↓AP-1 activity, ↓FosL1; FosL1 silencing suppressed TNF / IL-1β.	-
	[89]	**mouse:** pilocarpine SE / epileptogenesis	**Anti:** ↓hipp Fosl1 and broad infl-related genes; ↓cytokine / chemokine-related upregulation; ↓A2 astrogl reactivity markers.	-
	[90]	**mouse:** pilocarpine SE / acute epileptogenesis	**Anti:** ↓$CD11b^{+}CD45^{high}$ leukocyte infiltration; ↓$Ly6G^{+}Ly6C^{+}$ (lymphocyte antigen 6 complex loci C/G) infl neutrophils / monocytes; ↓broad infl cytokine panel in post-SE hippocampus.	-

	[91]	**cell culture / rat:** BV-2 + high glucose; STZ-diabetic rats	**Anti:** ↓MyD88 / TRAF6 / p-TAK1 / JNK / p38 / NF-κB; ↓TNF, ↓IL-1β, ↓IL-6; ↑IL-10, ↑CD206, ↓iNOS.	-
	[92]	**mouse:** pilocarpine-induced seizures, hippocampus	-	**Anti:** ↓LPO, ↓nitrite-nitrate; preserved CAT and GSH.
	[93]	**rat:** hippocampal microdialysis + ESR (electron spin resonance)	-	**Anti:** ↑endogenous AOX / free-radical scavenging capacity; ↓iNOS, ↑xCT.
	[94]	**rat:** chronic TLE; plus in vitro radical-scavenging assay	-	**Anti:** ↑SOD, ↑CAT, ↓ H_2O_2; direct HO• scavenging in vitro; no clear effect on LPO / carbonyls.
	[95]	**rat:** long-term TLE, 5.7 mo post-SE	-	**Anti:** ↓SOD/H_2O_2, ↓SOD/GPx, ↓SOD/(GPx+CAT) vs epileptic untreated; no significant change in H_2O_2 / MDA / carbonyls / 8-OHdG.
**Brivaracetam (BRV)**<br><br>**SV2A modulator**<br><br>High-affinity SV2A ligand with additional weak Na^+ channel effects at higher concentrations.<br><br>Used as adjunctive therapy for focal-onset seizures.	[96]	**mouse:** sciatic nerve cuff neuropathic pain, dorsal horn	**Anti:** ↓Iba1, ↓TNF, ↓CD3+ T-cell infiltration.	-
	[97]	**mouse:** cerebral ischemia/reperfusion	**Anti:** ↓TNF, ↑IL-10; ↓infarct burden; improved memory / motor outcome.	**Anti:** ↓TBARS, ↑GSH, ↑SOD, ↑CAT.
	[98]	**cell culture:** primary rat astrocytes; TNF / high-K^+-activated astrogl glutamate release	**Anti:** ↓TNF / high-K+-enhanced astrogl L-glu release; ↓plasma-membrane SV2A / Cx43 expression.	-
	[99]	**cell culture:** primary rat astrocytes; artificial HFO burst stimulation	**Anti:** ↓fast-ripple HFO-induced astrogl L-glu release; ↓plasma-membrane Cx43 / SV2A expression.	-
	[100]	**cell culture:** primary rat astrocyte-microglia co-cultures, M5 / M30 infl states	**Pro:** mild pro-infl glial shift: ↓resting ramified microglia, ↑rounded phagocytic microglia under infl conditions; Cx43 unchanged; overdose conc: ↓glial viability.	-

	[101]	**rat:** KA-TLE	**Pro:** ↑microglial activation, ↑astroglial density in multiple epileptic-circuit regions.	-
**Cannabidiol (CBD)**<br>**Multiple targets: TRPV, GPR55, adenosine-related signaling**<br>Modulates intracellular Ca^{2+}, interacts with TRPV channels, antagonizes GPR55, and inhibits ENT1 (equilibrative nucleoside transporter 1)-mediated adenosine uptake.<br>FDA-approved for Dravet syndrome and Lennox-Gastaut syndrome.	[102]	**cell culture / rat:** retinal microglia / retina + LPS	**Anti:** ↓TNF via ENT1 inhibition; enhanced adenosine-A2AAR-mediated anti-infl signaling.	-
	[103]	**mouse:** MCAO, post-ischemic phase	**Anti:** ↓MPO activity, ↓$MPO^{+}HMGB1^{+}$ cells, ↓plasma HMGB1, ↓Iba1 / GFAP (glial fibrillary acidic protein); improved neuro score / rotarod.	-
	[104]	**mouse:** delayed MCAO injury, repeated CBD from day 1 / 3 / 5	**Anti:** time-window anti-infl effect: when started day 1 or 3, ↓$Iba1^{+}HMGB1^{+}$ cells, ↓plasma HMGB1; improved survival / functional deficits.	-
	[105]	**rat:** sepsis / CLP (cecal ligation and puncture)	-	**Anti:** ↓TBARS, ↓protein carbonyls in brain / peripheral organs; improved cognition and mortality.
	[106]	**mouse:** hepatic I/R; isolated Kupffer cells / liver sinusoidal endothelial cells	**Anti:** ↓NF-κB activation, ↓TNF, ↓adhesion-molecule expression / neutrophil attachment.	**Anti:** ↓oxidative / nitrative stress and cell death.
	[107]	**rat:** chronic pilocarpine epilepsy, hipp	-	**Anti:** ↑hipp AOX defense in chronic epilepsy; with autophagy-linked protective signal.
	[108]	**mouse:** BCCAO (bilateral common carotid artery occlusion) ischemia	**Anti:** ↓glial response; ↓neuroinfl / white matter injury; improved cognitive-emotional outcome.	-
	[109]	**neonatal rat:** MCAO / arterial ischemic stroke	**Anti:** ↓astrogliosis, ↓microgl proliferation / activation; modulated neuroinfl with long-term functional recovery.	-

	[110]	**cell culture:** mouse hippocampal neurons; oxygen glucose deprivation/re-oxygenation (OGD/R)	-	**Anti:** ↓ROS, ↓LPO / MDA; ↑SOD1, ↑GPx, ↑GSH; improved mito respiration / ATP-linked OCR / spare capacity; preserved NADPH / $NADP^+$.
	[111]	**cell culture / rodent:** isolated brain mitochondria / hippocampal slices	-	**Pro:** context-dependent mito liability: at low-µM CBD ↓mito respiration, ↓ATP production, ↓Ca^{2+} uptake / mPT threshold; ↓hipp cell viability; impaired LTP / learning-memory at higher exposure.
**Stiripentol (STP)**<br>**GABA$_A$ modulator, enzyme inhibitor**	[112]	**cell culture:** rat cortical neuron-astroglia cultures; oxygen glucose deprivation (OGD) or glutamate	**Anti:** indirect anti-infl relevance only: ↓neuronal death / improved survival in mixed neuron-astroglia injury models.	**Anti:** indirect AOX / metabolic-protective relevance only: preserved neuronal survival during OGD / glutamate challenge; no direct ROS-marker panel.
Enhances GABA$_A$ receptor efficacy and elevates GABA via metabolic effects; also inhibits CYP enzymes, boosting clobazam exposure.<br>Used as adjunctive therapy in Dravet syndrome.	[113]	**gerbil:** transient forebrain ischemia, post-treatment	**Anti:** indirect anti-infl relevance only: ↓astrocyte damage / reactivity, ↓BBB leakage / IgG extravasation, ↓CA1 neuronal death; improved cognitive outcome.	-
	[114]	**cell culture:** primary mouse microglia + LPS / poly I:C	**Anti:** no prophylactic effect; in therapeutic paradigm after prior LPS activation, ↓Ptgs2 (prostaglandin-endoperoxide synthase 2), ↓pro-infl cytokine / chemokine release; less amoeboid microgl morphology.	-
**Fenfluramine (FFA)**<br>**5-HT releaser, 5-HT receptor agonist**	[115]	**rat:** acute LPS challenge	**Anti:** ↓plasma TNF, ↓IL-1β; ↑IL-10; systemic anti-infl cytokine shift.	-
	[116]	**cell culture / isolated mito / rat:** hepatocytes / liver mito; N-nitrosofenfluramine	-	**Pro:** ↓ATP, ↓GSH, ↓protein thiols; ↑GSSG, ↑MDA / LPO; mito dysfunction with later OS signal.

Amphetamine derivative that increases synaptic 5-HT via transporter-mediated mechanisms; norfenfluramine acts at 5-$HT_{2A/2B/2C}$ and 5-HT_{1D} receptors and fenfluramine-related antiseizure action also involves σ1R-linked signaling.<br><br>Low-dose oral solution is approved adjunctive therapy for seizures in Dravet and Lennox-Gastaut syndromes.	[117]	**isolated mito / cell culture / rat:** liver mitochondria / hepatocytes; N-nitrosofenfluramine	-	**Pro:** Ca^{2+}-dependent mPT induction, mito swelling, cytochrome c release, mitochondrial depolarization, ↓ATP.
	[118]	**mouse:** Scn1a$^{+/-}$ DS, chronic	**Anti:** ↓activated CD11b^{+} microglia; ↓D-MBP / myelin damage; ↓TUNEL labeling; ↑survival.	-

Abbreviations:
A2AAR, adenosine A2A receptor; **Aβ**, amyloid-beta; **BV-2**, BV-2 microglial cell line; **Cx43**, connexin 43; **DRESS**, drug reaction with eosinophilia and systemic symptoms; **GSH-Px**, glutathione peroxidase; **Hcy**, homocysteine; **HFO**, high-frequency oscillation; **MCAO**, middle cerebral artery occlusion; **MPO**, myeloperoxidase; **nNOS**, neuronal nitric oxide synthase; **NVU**, neurovascular unit; **OCR**, oxygen consumption rate; **OGD**, oxygen-glucose deprivation; **OGD/R**, oxygen-glucose deprivation/reoxygenation; **OSI**, oxidative stress index; **SCAR**, severe cutaneous adverse reaction; **SJS**, Stevens-Johnson syndrome; **TAC**, total antioxidant capacity; **TAK1**, transforming growth factor-beta-activated kinase 1; **TEN**, toxic epidermal necrolysis; **TOS**, total oxidant status; **TRAF6**, TNF receptor-associated factor 6; **vitB6**, vitamin B6; **vitB12**, vitamin B12.

Supplementary Table S2. Antiseizure effects of anti-inflammatory drugs (AIDs) and antioxidant pharmacology (AOX) in epilepsy.

AID, AOX, or drug class and main mechanism of action	Ref	Model	Pro- or anti-seizure activity / possible linked mechanism
**Anti-inflammatory drugs (AIDs)**			
**Selective COX-2 inhibitors (celecoxib, rofecoxib, etoricoxib)**<br><br>Selective COX-2 inhibition; ↓PGE_2 / prostanoid signaling; may ↓BBB P-gp upregulation.	[119]	**rat:** acute PTZ ± anti-PGE_2 Ab / PGE_2 i.c.v.	**Anti:** celecoxib 2 mg/kg protected vs PTZ seizures; anti-PGE_2 Ab anti-sz; exogenous PGE_2 reversed celecoxib effect.
	[120]	**mouse:** acute PTZ	**Anti:** celecoxib ↑clonic sz threshold / ↑latency; effect modulated by NO pathway.
	[121]	**rat:** pilocarpine SE / epileptogenesis	**Anti:** celecoxib pretx protected vs pilocarpine sz; ↓neuronal excitability; ↓microglia activation / astrogliosis / ectopic neurogenesis; prevented epileptogenesis-linked synaptic reorganization.
	[122]	**mouse:** ADLTE (autosomal dominant lateral temporal epilepsy) / $LGI1^{-/-}$	**Anti:** celecoxib rescued Kv1.2 defect, ↓sz susceptibility, ↑survival.
	[123]	**mouse:** acute PTZ threshold	**Anti:** rofecoxib ↑PTZ sz threshold; adenosinergic mechanism implicated.
	[124]	**mouse:** acute PTZ + PTZ kindling	**No effect:** prophylactic prandial rofecoxib did not attenuate acute PTZ sz, did not alter kindling acquisition, and did not reverse established kindling.
	[125]	**rat:** WAG/Rij absence epilepsy	**Anti:** etoricoxib acutely ↓SWD number and duration by ~50%; ELTT caused ~40% long-lasting reduction in spontaneous absence sz development, persisting 5 mo after withdrawal.
	[126]	**rat:** PTZ kindling	**Anti:** low-dose etoricoxib 1 mg/kg anticonvulsant; ↓latency/duration/severity after PTZ challenge; higher dose 10 mg/kg lost or reversed benefit.
**Nonselective NSAIDs and antipyretics (aspirin, indomethacin, ibuprofen, diclofenac)**	[127]	**mouse:** pilocarpine SE; aspirin pretx 3 d before to 6 d after SE	**Pro:** ↓SE latency, ↑mortality; no clear protection against SE-induced neuronal death or hippocampal astroglial / microglial response.
	[128]	**rat:** pilocarpine SE; aspirin started 0 h / 3 h / 24 h after SE	**Anti:** ↓freq and duration of SRS in chronic phase; ↓hipp neuronal loss; ↓MFS / aberrant neurogenesis.

Nonselective COX inhibition; prostanoid suppression; some agents also modulate BBB / inflammatory signaling.	[129]	**zebrafish larvae**: acute PTZ	**Anti:** indomethacin ↑sz latency, ↓number of sz-like behaviors; ↓*il1b*, ↓*cox2b*, ↓*c-fos* after PTZ.
	[130]	**rat:** PTZ epilepsy	**Anti:** ibuprofen ↓sz score; neuroprotective in hipp; ↓COX-2 / NLRP3 / IL-18; ↓neuronal excitability.
	[131]	**rat:** acute PTZ; behavior + EEG	**Anti:** ibuprofen ↓Racine score / spike %, ↑FMJ latency; ↓brain PGF2α.
	[132]	**mouse:** rotenone corneal kindling, mitochondrial DRE	**Anti:** diclofenac ↓sz score and ameliorated ASM resistance in post-tx validation; benefit linked to ↓OS and ↓quinolinic acid / KMO (kynurenine 3-monooxygenase) activity.
	[133]	**rat:** PTZ kindling	**Anti:** diclofenac 5 to 10 mg/kg ↓sz severity; ↓hipp IL-6 and TNF; direct antiseizure + anti-infl relevance.
	[134]	**clinical, RCT**: children with prior febrile sz; ibuprofen syrup during febrile illnesses	**No effect**: no reduction in febrile-sz recurrence vs placebo.
	[135]	**clinical, RCT**: children with prior febrile sz; antipyretics during febrile episodes	**No effect**: antipyretic agents ineffective for recurrent febrile-sz prevention.
**Corticosteroids / ACTH (methylprednisolone, dexamethasone, prednisolone / prednisone, hydrocortisone, ACTH)**<br><br>Broad glucocorticoid or steroidogenic immunosuppression; ↓cytokine transcription; BBB stabilization.	[136]	**mouse:** TMEV infection-induced acute seizures	**Anti:** dexamethasone moderately ↓acute seizure burden; **Anti:** prednisone moderately ↓acute seizure burden.
	[137]	**rat:** experimental febrile SE / epileptogenesis	**Anti:** dexamethasone ↓later hippocampal hyperexcitability / spike series; ↓IL-1β / IL-1R1; attenuated BBB disruption.
	[138]	**mouse:** NMDA-induced infantile spasms	**Anti:** ACTH high doses ↓number of spasms and seizure rating.
	[139]	**rat:** TTX-induced epileptic spasms / hypsarrhythmia-like EEG	**Anti:** ACTH gel 24 to 32 IU/kg/day eliminated spasms in 66% of animals; eliminated hypsarrhythmia-like activity in responders.
	[140]	**rodent:** post-early-life seizures; dexamethasone	**Anti:** dexamethasone attenuated later-life ↑sz susceptibility; ↓microglia activation; ↓neuronal injury.
	[141]	**clinical:** infants, Gibbs hypsarrhythmia / infantile spasms	**Anti:** historical landmark report; ACTH striking clinical efficacy in infantile spasms / hypsarrhythmia.
	[142]	**clinical, multicentre case series:** children with DRE; steroid tx protocols compared	**Anti:** corticosteroids after 6 mo, 62.7% had >50% sz reduction and 6.6% became sz-free; methylprednisolone 30 mg/kg/day for 3 d showed the highest response, with >50% sz reduction in 85.7%; dexamethasone and hydrocortisone were also active.

	[143]	**clinical:** children, genetic / suspected genetic DRE; pulsatile dexamethasone	**Anti:** ↓IEA (interictal epileptiform abnormality) burden after PCT (pulsatile corticosteroid therapy); ↓sz freq in 17/24 pts; 1 became sz-free.
	[144]	**clinical:** adults, AE with new-onset sz; immunotherapy registry	**Anti:** corticosteroid-based immunotherapy reduced sz burden in AE; mixed immunotherapy cohort; not steroid-specific.
	[145]	**clinical:** pediatric, older children with intractable epilepsy; oral prednisone add-on	**Anti:** 46% became sz-free; additional pts had marked sz reduction during treatment; best responses were reported in absence epilepsy and LGS (Lennox-Gastaut syndrome).
	[146]	**clinical:** childhood intractable epilepsy; mixed steroid experience + review	**Anti:** steroids / ACTH; 47% had ↓sz freq; 25% became sz-free.
	[147]	**clinical:** children, ESES (electrical status epilepticus during sleep) / CSWS and related disorders	**Anti:** ACTH-Z EEG remission in 2/5 trials; benefit was temporary.
	[148]	**clinical, RCT:** EE-SWAS; corticosteroids vs clobazam	**Anti:** supportive syndrome-level benefit in EE-SWAS; trial emphasis was mainly cognitive / EEG outcome.
	[149]	**clinical:** children with refractory epilepsy; IV methylpred	**Anti:** >50% sz reduction in 12/14 during treatment; sustained reduction persisted after treatment.
	[150]	**clinical:** children with intractable epilepsy; IV methylpred short course	**Anti:** safe and effective in a subset of patients with refractory epilepsy.
	[151]	**clinical:** children, LKS (Landau-Kleffner syndrome) / CSWS; corticosteroids	**Anti:** improvement in language / cognition / behavior; seizure outcome was not the sole endpoint.
**Tetracycline-class antibiotics (minocycline, doxycycline, tetracycline)**<br><br>Microglial inhibition; ↓IL-1β / TNF; ↓MMP-9 and post-insult inflammatory remodeling.	[152]	**mouse:** 6-Hz psychomotor sz; MES; scMET	**Anti:** minocycline, doxycycline, and tetracycline active in 6-Hz model; ED_{50} 170, 157, and 255 mg/kg, respectively; no effect in MES or scMET;<br>**Pro/caution:** minocycline and doxycycline high doses caused toxicity / motor impairment.
	[153]	**rat:** amygdala kindling	**Anti:** minocycline ↓ADD; ↓S5D; ↓SD; ↑S4L; daily 25 mg/kg also ↓ADD / S5D / SD.

	[154]	**mouse:** PTZ kindling	**Anti:** minocycline given 1 h before PTZ ↑latency to stage 4 sz; ↓duration of stages 4 and 5; prevented ↑TNF receptor mRNA in hippocampus and piriform ctx; normalized PTZ-induced receptor-subunit changes; post-PTZ dosing was ineffective.
	[155]	**rat:** lithium-pilocarpine SE / chronic phase	**Anti:** minocycline ↓freq / duration / severity of SRS; prevented SE-induced neuronal loss; ↓microgl activation; ↓IL-1β / TNF in hippocampal CA1 and adjacent ctx; no clear effect on astrogl activation.
	[156]	**mouse:** TMEV infection-induced acute seizures / chronic TLE-like outcomes	**Anti:** minocycline ↑latency to first seizure; ↓acute seizure burden; normalized later PTZ seizure threshold; improved long-term behavioral outcomes; no clear effect on maximum observed Racine stage severity.
	[157]	**rat:** WAG/Rij absence epilepsy	**Pro:** doxycycline ↑incidence and duration of SWDs.
	[158]	**rat:** electrically induced SE / epileptogenesis	**No effect:** minocycline no reduction in spontaneous sz development;<br>**Anti:** minocycline ↓hyperactivity / hyperlocomotion; improved spatial learning; selective neuroprotection in piriform ctx / hilus; authors did not verify strong anti-inflammatory effects in this model.
	[159]	**clinical:** astrocytoma-associated DRE; case report	**Anti:** minocycline, marked reduction in seizure frequency during therapy.
	[160]	**clinical:** chronic FIRES; 3-patient case series	**No effect:** minocycline, no significant reduction in seizure frequency; one patient had shorter seizures limited to focal seizures and improved quality-of-life score.
**Anakinra**<br><br>Recombinant IL-1 receptor antagonist; blocks IL-1α / IL-1β signaling at IL-1R1.	[161]	**rat:** lithium-pilocarpine epileptogenesis / chronic TLE	**Anti:** during latent phase ↓freq and duration of SRS in chronic phase; prevented behavioral impairments; neuroprotective; no clear suppression of gliosis / Il1b / Tnfa upregulation.
	[162]	**rat:** lithium-pilocarpine epileptogenesis; anakinra ± LTG	**Anti:** no clear ↓number or duration of SRS; ↓sz severity; ↓CA1 neuronal loss; behavioral benefit.
	[163]	**rat:** pilocarpine SE and electrical SE; anakinra + VX-765 post-SE	**No effect:** onset of epilepsy and later sz freq / duration not significantly modified;<br>**Anti:** ↓IL-1β in astroglia; ↓cell loss.
	[164]	**clinical:** pediatric FIRES / super-refractory SE; case report	**Anti:** rapid EEG / clinical sz improvement; cytokines normalized.

	[165]	**clinical:** international retrospective cohort; pediatric FIRES	**Anti:** associated with reduction / termination of refractory sz burden in a substantial subset; earlier use associated with shorter ventilation, ICU stay, and hospital stay.
	[166]	**clinical:** adult Rasmussen encephalitis; case report	**Anti:** marked ↓daily sz burden after late introduction; then sz freedom; withdrawal caused relapse; reintroduction restored control.
**Canakinumab**<br><br>Anti-IL-1β monoclonal antibody; selective IL-1β neutralization.	[167]	**clinical:** adolescent; systemic autoinfl + intractable generalized epilepsy	**Anti:** near-complete resolution of clinical sz after sequential IL-1 blockade with anakinra then canakinumab; marked improvement in social / academic functioning; peripheral inflammatory gene-expression signature was extinguished after anakinra treatment.
**Tocilizumab**<br><br>Anti-IL-6 receptor monoclonal antibody; blocks IL-6R signaling.	[168]	**rat:** WAG/Rij absence epilepsy; acute and early long-term tocilizumab	**Anti:** acute 30 mg/kg ↓SWD activity for ~25 h; early long-term treatment ↓development of absence sz at 6 mo and 10 mo after treatment withdrawal; ↓depressive-like behavior; blocked LPS- and IL-6-induced worsening of absence sz.
	[169]	**clinical:** adults; NORSE (new-onset refractory status epilepticus), 7 pts, refractory to conventional immunotherapy ± rituximab	**Anti:** SE terminated after 1 to 2 doses in 6/7; median 3 d from initiation; no SE recurrence during observation.
	[170]	**clinical:** child; anakinra-refractory FIRES; tocilizumab + ketogenic diet	**Anti:** seizure cessation temporally associated with tocilizumab introduction; favorable 1-y outcome.
	[171]	**clinical:** chronic-phase FIRES; retrospective case series, 5 pts on anti-IL therapy; 1 switched from anakinra to tocilizumab	**Anti:** with anti-IL therapy, 20 to 50% ↓sz freq in 3/5 at 6 mo; ↓sz intensity; ↓rescue-med need; better behavior / communication in 3/5; similar improvement after switch to tocilizumab.
**Adalimumab**<br><br>Anti-TNF monoclonal antibody; TNF pathway blockade.	[172]	**clinical:** Rasmussen encephalitis, open-label pilot, 11 pts	**Anti:** >50% ↓sz freq in 5/11, including seizure freedom in 1; stabilization of motor / cognitive decline in 3/11; median tx delay 31 mo; median follow-up 18 mo.
**VX-765**<br><br>Caspase-1 inhibitor; ↓IL-1β maturation and inflammasome-linked excitability amplification.	[173]	**mouse:** acute KA sz; chronic spontaneous epileptic activity refractory to common ASMs	**Anti:** ↑latency to acute sz onset; ↓cumulative acute sz duration; in chronic epilepsy, dose-dependent ↓epileptic activity at doses ≥50 mg/kg; effect reversed after withdrawal.
	[163]	**rat:** electrically induced SE and pilocarpine SE; post-SE tx with VX-765 + anakinra	**No effect:** no significant change in epilepsy onset, chronic sz freq, or sz duration;<br>**Anti:** ↓IL-1β in astroglia; ↓cell loss / neurodegeneration after SE.

**Montelukast (MTK)**<br><br>CysLT1 antagonist; blocks leukotriene signaling at the BBB / inflammation interface.	[174]	**rat:** acute PTZ, behavior + EEG	**Anti:** 50 to 100 mg/kg ↓Racine score; ↑FMJ latency; 25 to 100 mg/kg ↓brain MDA; ↑SOD; 50 to 100 mg/kg ↓EEG spike %.
	[175]	**mouse:** acute PTZ, i.c.v. CysLT antagonists, EEG + BBB assays	**Anti:** 0.03 to 0.3 μmol ↑latency to generalized sz; ↓EEG amplitude; prevented PTZ-induced BBB disruption; LTD4 reversed anticonvulsant effect.
	[176]	**mouse:** PTZ kindling	**Anti:** 10 mg/kg ↑latency to myoclonic jerk and generalized tonic-clonic sz after PTZ challenge; altered cortical CysLT1 / CysLT2 immunoreactivity.
	[177]	**mouse:** acute PTZ, isobolographic add-on with phenobarbital	**Anti:** synergistically potentiated phenobarbital anticonvulsant effect vs PTZ-induced sz; adjunctive benefit shown by behavioral + EEG analyses.
	[178]	**mouse:** PTZ kindling and pilocarpine SE with spontaneous recurrent seizures	**Anti:** dose-dependently suppressed development of kindled seizures; dose-dependently suppressed pilocarpine-induced spontaneous recurrent seizures.
**Fingolimod (FTY720)**<br><br>Functional S1P receptor modulator; central glial / inflammatory signaling and BBB-P-gp effects.	[179]	**rat:** lithium-pilocarpine SE, FTY720 1 mg/kg/day i.p. for 14 d starting 24 h after SE	**Anti:** ↓incidence; ↓duration; ↓freq; ↓severity of SCs (spontaneous convulsions) during d21-34 post-SE; ↓MFS; ↓neuronal loss; ↓microgl / astrogl activation; ↓IL-1β; ↓TNF in hipp.
	[180]	**rat:** WAG/Rij absence epilepsy, ELTT 1 mg/kg/day starting before sz onset, 17 wk	**Anti:** ↓nSWDs; ↓dSWDs (number and duration of spike-wave discharges) at 6 mo; antiepileptogenic effect temporary; lost 5 mo after withdrawal.
	[181]	**rat:** pilocarpine SE, FTY720 ± W146 (sphingosine-1-phosphate receptor 1 antagonist); hipp microdialysis after PHT	**Anti:** ↓hipp P-gp; ↓NF-κB; ↓TNF; ↓COX-2; ↑hipp extracellular PHT after SE.
	[182]	**rat:** acute penicillin-induced cortical epileptiform activity, FTY720 0.3 mg/kg ± diazepam	**No clear effect:** slight ↓spike-wave activity and spike amplitudes, NS;<br>**Anti:** ↓serum IL-1β; combo with diazepam ↓IL-6.
	[183]	**rat:** PTZ-kindling-induced phenobarbital-resistant epilepsy	**Anti:** ↓seizure score dose-dependently; alleviated BBB permeability; ↓hippocampal P-gp expression; ↑brain phenobarbital delivery.
**Antioxidant pharmacology (AOXs)**			
**N-acetylcysteine (NAC)**	[184]	**rat:** acute PTZ, behavior + EEG.	**Anti:** 300 and 600 mg/kg ↑FMJ latency; ↓Racine stage; ↓EEG spike %; dose-dependent.

Thiol donor and cysteine prodrug; replenishes GSH and modulates xCT-linked glutamatergic tone.	[185]	**mouse:** acute and chronic PTZ, i.p.	**Anti:** acute and chronic dose-dependently ↑latency to myoclonic and clonic sz; chronic effect > acute; no protection vs PTZ-induced death.
	[186]	**rat:** KA-TLE, oral NAC 7-d pretx + 1-d post-tx	**Anti:** delayed sz activity; ↑intact neurons in CA3 / hilus; improved spatial memory.
	[187]	**rat:** KA-TLE, oral NAC 7-d pretx + 1-d post-tx	**Anti:** ↓mTOR; ↓Drp1; improved mito dynamics and membrane potential after epilepsy induction.
	[188]	**rat:** electrical SE / epileptogenesis; NAC + sulforaphane during 2-wk post-SE window	**Anti:** delayed epilepsy onset; blocked disease progression between 2 and 5 mo post-SE; ↓freq of spontaneous sz at 5 mo; no clear change in mean sz duration or epilepsy incidence.
	[189]	**rat:** WAG/Rij absence epilepsy	**Pro:** ↑number and duration of SWDs; improved depressive-like and cognitive comorbidities; linked to ↑brain mGlu2 receptor expression.
	[190]	**clinical:** progressive myoclonus epilepsy of Unverricht-Lundborg type; 4 siblings	**Anti:** marked ↓generalized tonic-clonic sz frequency during adjunct NAC 4 to 6 g/day; myoclonus improved inconsistently.
	[191]	**clinical:** Unverricht-Lundborg disease; 4 patients	**Anti:** improvement in seizures during NAC treatment with parallel rise in glutathione; variable response; notable side effects in some patients.
**Dimethyl fumarate (DMF)**<br><br>Electrophilic KEAP1-Nrf2 activator; immunoredox modulation, also linked to HCAR2 signaling.	[192]	**rat:** PTZ kindling	**Anti:** ↓kindling severity; ↓% kindled animals; ↓neuronal damage in hipp; ↑GPx; ↑SOD; ↑GSH; ↓LPO.
	[193]	**rat:** PTZ kindling, hipp	**Anti:** ↓sz score; ↓% kindled rats; ↓neurological damage; ↓IL-6; ↓IL-1β; ↓TNF; ↓NF-κB; ↓Bax; ↓caspase-3; ↑Nrf2; ↑HO-1; ↑NQO1; ↑Bcl-2.
	[194]	**mouse:** intrahipp KA-SE, DMF 1 wk before SE and continued after SE; SRS monitored 21 d	**Anti:** ↓SRS 7 to 21 d post-SE; ↓GSDMD-N (N-terminal gasdermin D); ↓cleaved caspase-1; ↓IL-1β; ↓IL-18; ↓microglia; ↓clasmatodendritic astroglia.
	[195]	**rat:** KA-SE, DMF 200 mg/kg/day i.p. for 7 d post-SE; additional delayed-tx epilepsy cohort	**Anti:** ↓sz freq; ↓total sz number; ↑Nrf2 activity; ↓neuronal death; improved behavioral deficits; delayed-tx also ↓symptomatic sz long after drug elimination.
	[196]	**mouse:** PTZ kindling	**Anti:** ↑latency of epileptic seizures; ↓MDA / ROS / 8-OHdG; ↑SOD; reduced mitochondrial abnormalities; ↑Nrf2.
**Tempol**	[197]	**rat:** picrotoxin-induced epilepsy	**Anti:** 100 and 150 mg/kg ↓total sz number; ↓total sz duration; 150 mg/kg also ↓seizure phases.

Membrane-permeable nitroxide; catalytic redox scavenger with SOD-mimetic activity.	[198]	**mouse:** peripheral LPS infl, then KA-induced sz susceptibility; i.c.v. tempol infusion	**Anti:** ameliorated LPS-driven ↑seizure susceptibility to KA; protected hippocampus from oxidative damage; no clear effect on microglial activation or cytokine production.
	[199]	**rat:** picrotoxin-induced epilepsy	**Anti:** 150 mg/kg was highly effective in suppressing generalized seizures.
**EUK-134**<br><br>Synthetic SOD / catalase mimetic.	[200]	**rat:** systemic KA-induced szs, limbic oxidative injury / neuropathology	**Anti:** no material change in seizure latency or duration; ↓protein nitration; ↓AP-1 and NF-κB binding; ↓spectrin proteolysis; ↓neuronal damage in limbic structures.
**Ebselen**<br><br>GPx-mimetic organoselenium compound; peroxide detoxification plus IMPase inhibition.	[201]	**mouse:** pilocarpine challenge, sub-seizure paradigm; ebselen at different doses / time intervals before pilocarpine	**Anti:** no potentiation of pilocarpine-induced sz liability; ↓pilocarpine-induced tremor; ↓*c-fos* mRNA induction.
**Elamipretide (SS-31)**<br><br>Mitochondria-targeted cardiolipin-binding peptide; stabilizes inner mitochondrial membrane bioenergetics and limits cardiolipin peroxidation.	[202]	**rat:** pilocarpine SE	**Anti:** ↓seizure severity; ↓hipp neuronal injury; ↓Fe2+; ↓MDA; ↓4-HNE; ↓p-p38 MAPK; ↓ACSL4; ↓COX-2; ↑GPX4; ↑FTH1; ↑SLC7A11.
**Idebenone**<br><br>Short-chain benzoquinone / CoQ analogue; supports electron transfer distal to complex I and improves bioenergetic resilience.	[203]	**rat:** pilocarpine-induced sz / SE	**Anti:** ↑latency to first generalized sz; ↓% animals with generalized tonic-clonic sz; ↑survival; ↓hipp neuronal damage; ↓MDA; ↓NO; ↑GSH; ↑CAT.
	[204]	**mouse:** rotenone corneal kindling, mitochondrial DRE	**Anti:** reversed refractoriness to ASMs; ↓kindled sz severity; improved bioenergetic / mitochondrial function indexes in rotenone corneal kindling model.
**Vatiquinone (EPI-743)**<br><br>Redox-active quinone / anti-ferroptotic 15-lipooxygenase(LOX)-directed strategy; limits LPO.	[205]	**cell culture:** patient-derived fibroblasts / B-lymphocytes from mitochondrial disease-associated epilepsies	**No direct seizure experiment.**<br>**Anti:** ↓ferroptosis; ↓general LPO; ↓15-HETE (15-hydroxyeicosatetraenoic acid); rescued RSL3- and BSO/Fe-induced cell death.
	[206]	**cell culture / mouse:** human dermal fibroblasts (HDFs); tamoxifen-inducible $Gpx4^{-}$deficient mice; $Ndufs4^{-/-}$ Leigh-syndrome mice	**Anti:** in vitro fully prevented RSL3- and BSO/Fe-induced cell death; in vivo no benefit on disease onset / progression / survival; seizure risk may be ↓ in $Ndufs4^{-/-}$ mice, with no seizures observed in treated animals during P30 rotarod assay.

**Melatonin**<br><br>MT1 / MT2 agonist; chronobiotic plus direct and indirect AOX actions.	[207]	**rat:** KA-SE / epileptogenesis; melatonin in drinking water for 8 wk after SE	**Anti:** ↑latency to appearance of SRSs; ↓SRS frequency only during treatment period; ↓CA1 / piriform neuronal damage; improved hyperactivity / depression-like behavior / working memory; no clear suppression of epileptogenesis.
	[208]	**rat:** spontaneously hypertensive rats (SHR); KA-SE / TLE, melatonin in drinking water for 8 wk during epileptogenesis	**Anti:** attenuated seizure activity; ↓neuronal damage in CA1 and piriform cortex;<br>**No clear effect:** did not prevent disturbance in diurnal rhythms or behavioral alterations.
	[209]	**clinical:** intractable epilepsy, randomized crossover pilot, melatonin 10 mg qhs for 3 wk	**Anti:** ↓diurnal sz vs placebo; sleep and behavior improved.
	[210]	**clinical:** children with epilepsy, randomized double-blind crossover, sustained-release melatonin	**No clear effect:** no clear change in sz freq or spike density;<br>**Anti:** ↓sleep latency; ↓WASO.
	[211]	**clinical:** adults with idiopathic generalized tonic-clonic seizures alone, randomized double-blind placebo-controlled crossover	**Anti:** ↓seizure severity score; no clear change in attack number or EEG abnormalities; sleep quality improved.
	[212]	**clinical:** IESS (infantile epileptic spasms syndrome), randomized placebo-controlled double-blind add-on trial	**No clear effect:** spasm control rate ↑ numerically vs placebo, NS;<br>**Anti:** no melatonin-related adverse effects; sleep quality improved; sleep-onset latency shortened.

Abbreviations:
ACTH, adrenocorticotropic hormone; **ADD**, afterdischarge duration; **AE**, autoimmune encephalitis; **BSO**, buthionine sulfoximine; **CSWS**, continuous spike-and-waves during slow sleep; **EE-SWAS**, developmental and epileptic encephalopathy with spike-wave activation in sleep; **ELTT**, early long-term treatment; **FMJ**, first myoclonic jerk; **i.c.v.**, intracerebroventricular; **MFS**, mossy fiber sprouting; **Ndufs4**, NADH:ubiquinone oxidoreductase core subunit S4; **NS**, not significant; **S4L**, stage 4 latency; **S5D**, stage 5 duration; **SD**, seizure duration; **SRS**, spontaneous recurrent seizures; **SWD**, spike-wave discharge; **WAG/Rij**, Wistar Albino Glaxo/Rijswijk.

References

1. Bianchi, M.; Rossoni, G.; Sacerdote, P.; Panerai, A.E.; Berti, F. Carbamazepine exerts anti-inflammatory effects in the rat. *Eur. J. Pharmacol.* **1995**, *294*, 71–74, doi:10.1016/0014-2999(95)00516-1.
2. Matoth, I.; Pinto, F.; Sicsic, C.; Brenner, T. Inhibitory effect of carbamazepine on inflammatory mediators produced by stimulated glial cells. *Neurosci. Res.* **2000**, *38*, 209–212, doi:10.1016/s0168-0102(00)00127-9.
3. Wang, C.-H.; Hsiao, C.-J.; Lin, Y.-N.; Wu, J.-W.; Kuo, Y.-C.; Lee, C.-K.; Hsiao, G. Carbamazepine attenuates inducible nitric oxide synthase expression through Akt inhibition in activated microglial cells. *Pharm. Biol.* **2014**, *52*, 1451–1459, doi:10.3109/13880209.2014.898074.
4. Gómez, C.D.; Buijs, R.M.; Sitges, M. The anti-seizure drugs vinpocetine and carbamazepine, but not valproic acid, reduce inflammatory IL-1β and TNF-α expression in rat hippocampus. *J. Neurochem.* **2014**, *130*, 770–779, doi:10.1111/jnc.12784.
5. Okada, M.; Fukuyama, K.; Shiroyama, T.; Ueda, Y. Carbamazepine Attenuates Astroglial L-Glutamate Release Induced by Pro-Inflammatory Cytokines via Chronically Activation of Adenosine A2A Receptor. *Int. J. Mol. Sci.* **2019**, *20*, doi:10.3390/ijms20153727.
6. Zhou, T.; Wang, N.; Xu, L.; Huang, H.; Yu, C.; Zhou, H. Effects of carbamazepine combined with vitamin B12 on levels of plasma homocysteine, hs-CRP and TNF-α in patients with epilepsy. *Exp. Ther. Med.* **2018**, *15*, 2327–2332, doi:10.3892/etm.2018.5698.
7. Genin, E.; Chen, D.P.; Hung, S.I.; Sekula, P.; Schumacher, M.; Chang, P.Y.; Tsai, S.H.; Wu, T.L.; Bellón, T.; Tamouza, R.; Fortier, C.; Toubert, A.; Charron, D.; Hovnanian, A.; Wolkenstein, P.; Chung, W.H.; Mockenhaupt, M.; Roujeau, J.C. HLA-A*31:01 and different types of carbamazepine-induced severe cutaneous adverse reactions: an international study and meta-analysis. *Pharmacogenomics J.* **2014**, *14*, 281–288, doi:10.1038/tpj.2013.40.
8. Higuchi, S.; Yano, A.; Takai, S.; Tsuneyama, K.; Fukami, T.; Nakajima, M.; Yokoi, T. Metabolic activation and inflammation reactions involved in carbamazepine-induced liver injury. *Toxicol. Sci.* **2012**, *130*, 4–16, doi:10.1093/toxsci/kfs222.
9. Tutanc, M.; Aras, M.; Dokuyucu, R.; Altas, M.; Zeren, C.; Arica, V.; Ozturk, O.H.; Motor, S.; Yilmaz, C. Oxidative status in epileptic children using carbamazepine. *Iran. J. Pediatr.* **2015**, *25*, e3885, doi:10.5812/ijp.3885.
10. Işık, M.; Demir, Y.; Kırıcı, M.; Demir, R.; Şimşek, F.; Beydemir, Ş. Changes in the anti-oxidant system in adult epilepsy patients receiving anti-epileptic drugs. *Arch. Physiol. Biochem.* **2015**, *121*, 97–102, doi:10.3109/13813455.2015.1026912.
11. Jakovljević, D.; Nikolić, M.; Jovanović, V.; Vidonja Uzelac, T.; Nikolić-Kokić, A.; Novaković, E.; Miljević, Č.; Milovanović, M.; Blagojević, D. Influence of Long-Term Anti-Seizure Medications on Redox Parameters in Human Blood. *Pharmaceuticals (Basel)* **2024**, *17*, doi:10.3390/ph17010130.
12. Maheswari, E.; Saraswathy, G.R.L.; Santhranii, T. Hepatoprotective and antioxidant activity of N-acetyl cysteine in carbamazepine-administered rats. *Indian J. Pharmacol.* **2014**, *46*, 211–215, doi:10.4103/0253-7613.129321.
13. Alelwani, W.; Elmorsy, E.; Kattan, S.W.; Babteen, N.A.; Alnajeebi, A.M.; Al-Ghafari, A.; Carter, W.G. Carbamazepine induces a bioenergetics disruption to microvascular endothelial cells from the blood-brain barrier. *Toxicol. Lett.* **2020**, *333*, 184–191, doi:10.1016/j.toxlet.2020.08.006.
14. Eghbal, M.A.; Taziki, S.; Sattari, M.R. Protective Role of Melatonin and Taurine Against Carbamazepine-induced Toxicity in Freshly Isolated Rat Hepatocytes. *Int. J. Morphol.* **2013**, *31*, 1081–1089, doi:10.4067/S0717-95022013000300049.
15. Xu, Y.; Zhang, N.; Xu, S.; Xu, H.; Chen, S.; Xia, Z. Effects of phenytoin on serum levels of homocysteine, vitamin B12, folate in patients with epilepsy: A systematic review and meta-analysis (PRISMA-compliant article). *Medicine (Baltimore)* **2019**, *98*, e14844, doi:10.1097/MD.0000000000014844.
16. Mintzer, S.; Skidmore, C.T.; Abidin, C.J.; Morales, M.C.; Chervoneva, I.; Capuzzi, D.M.; Sperling, M.R. Effects of antiepileptic drugs on lipids, homocysteine, and C-reactive protein. *Ann. Neurol.* **2009**, *65*, 448–456, doi:10.1002/ana.21615.
17. Viera, M.H.; Perez, O.A.; Patel, J.K.; Jones, I.; Berman, B. Phenytoin-associated hypersensitivity syndrome with features of DRESS and TEN/SJS. *Cutis.* **2010**, *85*, 312–317.

18. Tawfik, M.K. Coenzyme Q10 enhances the anticonvulsant effect of phenytoin in pilocarpine-induced seizures in rats and ameliorates phenytoin-induced cognitive impairment and oxidative stress. *Epilepsy Behav.* **2011**, *22*, 671–677, doi:10.1016/j.yebeh.2011.09.018.

19. Eghbal, M.A.; Taziki, S.; Sattari, M.R. Mechanisms of phenytoin-induced toxicity in freshly isolated rat hepatocytes and the protective effects of taurine and/or melatonin. *J. Biochem. Mol. Toxicol.* **2014**, *28*, 111–118, doi:10.1002/jbt.21542.

20. Martinc, B.; Grabnar, I.; Milosheska, D.; Lorber, B.; Vovk, T. A Cross-Sectional Study Comparing Oxidative Stress in Patients with Epilepsy Treated with Old and New Generation Antiseizure Medications. *Medicina (Kaunas)* **2024**, *60*, doi:10.3390/medicina60081299.

21. Arhan, E.; Serdaroglu, A.; Ozturk, B.; Ozturk, H.S.; Ozcelik, A.; Kurt, N.; Kutsal, E.; Sevinc, N. Effects of epilepsy and antiepileptic drugs on nitric oxide, lipid peroxidation and xanthine oxidase system in children with idiopathic epilepsy. *Seizure* **2011**, *20*, 138–142, doi:10.1016/j.seizure.2010.11.003.

22. Bolayir, E.; Celik, K.; Tas, A.; Topaktas, S.; Bakir, S. The effects of oxcarbazepine on oxidative stress in epileptic patients. *Methods Find. Exp. Clin. Pharmacol.* **2004**, *26*, 345–348, doi:10.1358/mf.2004.26.5.831325.

23. Agarwal, N.B.; Agarwal, N.K.; Mediratta, P.K.; Sharma, K.K. Effect of lamotrigine, oxcarbazepine and topiramate on cognitive functions and oxidative stress in PTZ-kindled mice. *Seizure* **2011**, *20*, 257–262, doi:10.1016/j.seizure.2010.12.006.

24. Faustmann, T.J.; Corvace, F.; Faustmann, P.M.; Ismail, F.S. Effects of Lamotrigine and Topiramate on Glial Properties in an Astrocyte-Microglia Co-Culture Model of Inflammation. *Int. J. Neuropsychopharmacol.* **2022**, *25*, 185–196, doi:10.1093/ijnp/pyab080.

25. Du, X.; Wang, B.; Wang, H.; Li, Q.; Li, X.; Hu, P.; Lai, Q.; Fan, H. Is the regulation of lamotrigine on depression in patients with epilepsy related to cytokines? *Heliyon* **2024**, *10*, e33129, doi:10.1016/j.heliyon.2024.e33129.

26. Arora, T.; Mehta, A.K.; Sharma, K.K.; Mediratta, P.K.; Banerjee, B.D.; Garg, G.R.; Sharma, A.K. Effect of carbamazepine and lamotrigine on cognitive function and oxidative stress in brain during chemical epileptogenesis in rats. *Basic Clin. Pharmacol. Toxicol.* **2010**, *106*, 372–377, doi:10.1111/j.1742-7843.2009.00499.x.

27. Haggag, B.S.; Hasanin, A.H.; Raafat, M.H.; Abdel Kawy, H.S. Lamotrigine decreased hippocampal damage and improved vascular risk markers in a rat model of pentylenetetrazole induced kindling seizure. *Korean J. Physiol. Pharmacol.* **2014**, *18*, 269–278, doi:10.4196/kjpp.2014.18.3.269.

28. Abelaira, H.M.; Réus, G.Z.; Ribeiro, K.F.; Steckert, A.V.; Mina, F.; Rosa, D.V.F.; Santana, C.V.; Romano-Silva, M.A.; Dal-Pizzol, F.; Quevedo, J. Effects of lamotrigine on behavior, oxidative parameters and signaling cascades in rats exposed to the chronic mild stress model. *Neurosci. Res.* **2013**, *75*, 324–330, doi:10.1016/j.neures.2013.02.002.

29. Eren, I.; Naziroğlu, M.; Demirdaş, A. Protective effects of lamotrigine, aripiprazole and escitalopram on depression-induced oxidative stress in rat brain. *Neurochem. Res.* **2007**, *32*, 1188–1195, doi:10.1007/s11064-007-9289-x.

30. Hossain, M.M.; Weig, B.; Reuhl, K.; Gearing, M.; Wu, L.-J.; Richardson, J.R. The anti-parkinsonian drug zonisamide reduces neuroinflammation: Role of microglial Nav 1.6. *Exp. Neurol.* **2018**, *308*, 111–119, doi:10.1016/j.expneurol.2018.07.005.

31. Mori, A.; Noda, Y.; Packer, L. The anticonvulsant zonisamide scavenges free radicals. *Epilepsy Res.* **1998**, *30*, 153–158, doi:10.1016/s0920-1211(97)00097-1.

32. Komatsu, M.; Hiramatsu, M.; Willmore, L.J. Zonisamide reduces the increase in 8-hydroxy-2'-deoxyguanosine levels formed during iron-induced epileptogenesis in the brains of rats. *Epilepsia* **2000**, *41*, 1091–1094, doi:10.1111/j.1528-1157.2000.tb00312.x.

33. Yürekli, V.A.; Gürler, S.; Nazıroğlu, M.; Uğuz, A.C.; Koyuncuoğlu, H.R. Zonisamide attenuates MPP+-induced oxidative toxicity through modulation of Ca2+ signaling and caspase-3 activity in neuronal PC12 cells. *Cell. Mol. Neurobiol.* **2013**, *33*, 205–212, doi:10.1007/s10571-012-9886-3.

34. Asanuma, M.; Miyazaki, I.; Diaz-Corrales, F.J.; Kimoto, N.; Kikkawa, Y.; Takeshima, M.; Miyoshi, K.; Murata, M. Neuroprotective effects of zonisamide target astrocyte. *Ann. Neurol.* **2010**, *67*, 239–249, doi:10.1002/ana.21885.

35. Choudhury, M.E.; Sugimoto, K.; Kubo, M.; Iwaki, H.; Tsujii, T.; Kyaw, W.T.; Nishikawa, N.; Nagai, M.; Tanaka, J.; Nomoto, M. Zonisamide up-regulated the mRNAs encoding astrocytic anti-oxidative and neurotrophic factors. *Eur. J. Pharmacol.* **2012**, *689*, 72–80, doi:10.1016/j.ejphar.2012.05.012.

36. Shishmanova-Doseva, M.; Peychev, L.; Yoanidu, L.; Uzunova, Y.; Atanasova, M.; Georgieva, K.; Tchekalarova, J. Anticonvulsant Effects of Topiramate and Lacosamide on Pilocarpine-Induced Status Epilepticus in Rats: A Role of Reactive Oxygen Species and Inflammation. *Int. J. Mol. Sci.* **2021**, *22*, doi:10.3390/ijms22052264.

37. Shishmanova-Doseva, M.; Atanasova, D.; Uzunova, Y.; Yoanidu, L.; Peychev, L.; Marinov, P.; Tchekalarova, J. Effects of lacosamide treatment on epileptogenesis, neuronal damage and behavioral comorbidities in a rat model of temporal lobe epilepsy. *Int. J. Mol. Sci.* **2021**, *22*, doi:10.3390/ijms22094667.

38. Agarwal, S.; Vyas, P.; Nirwan, N.; Vohora, D. Effect of lacosamide on neuroinflammation-mediated seizures comorbid with depression in C57BL/6 mice-Role of kynurenine pathway. *Epilepsy Behav.* **2021**, *123*, 108262, doi:10.1016/j.yebeh.2021.108262.

39. Nirwan, N.; Siraj, F.; Vohora, D. Inverted-U response of lacosamide on pilocarpine-induced status epilepticus and oxidative stress in C57BL/6 mice is independent of hippocampal collapsin response mediator protein-2. *Epilepsy Res.* **2018**, *145*, 93–101, doi:10.1016/j.eplepsyres.2018.06.009.

40. Lazzarini, R.; Malucelli, B.E.; Palermo-Neto, J. Reduction of acute inflammation in rats by diazepam: role of peripheral benzodiazepine receptors and corticosterone. *Immunopharmacol. Immunotoxicol.* **2001**, *23*, 253–265, doi:10.1081/iph-100103864.

41. Falcón, C.R.; Hurst, N.F.; Vivinetto, A.L.; López, P.H.H.; Zurita, A.; Gatti, G.; Cervi, L.; Monferran, C.G.; Roth, G.A. Diazepam impairs innate and adaptive immune responses and ameliorates experimental autoimmune encephalomyelitis. *Front. Immunol.* **2021**, *12*, 682612, doi:10.3389/fimmu.2021.682612.

42. Lazzarini, R.; Malucelli, B.E.; Muscará, M.N.; de Nucci, G.; Palermo-Neto, J. Reduction of inflammation in rats by diazepam: tolerance development. *Life Sci.* **2003**, *72*, 2361–2368, doi:10.1016/s0024-3205(03)00136-x.

43. Méndez-Cuesta, L.A.; Márquez-Valadez, B.; Pérez-De La Cruz, V.; Escobar-Briones, C.; Galván-Arzate, S.; Alvarez-Ruiz, Y.; Maldonado, P.D.; Santana, R.A.; Santamaría, A.; Carrillo-Mora, P. Diazepam blocks striatal lipid peroxidation and improves stereotyped activity in a rat model of acute stress. *Basic Clin. Pharmacol. Toxicol.* **2011**, *109*, 350–356, doi:10.1111/j.1742-7843.2011.00738.x.

44. Amouzad Mahdirejei, H.; Peeri, M.; Azarbayjani, M.A.; Masrour, F.F. Diazepam and exercise training combination synergistically reduces lipopolysaccharide-induced anxiety-like behavior and oxidative stress in the prefrontal cortex of mice. *Neurotoxicology* **2023**, *97*, 101–108, doi:10.1016/j.neuro.2023.06.004.

45. Schwaninger, M.; Ringleb, P.; Winter, R.; Kohl, B.; Fiehn, W.; Rieser, P.A.; Walter-Sack, I. Elevated plasma concentrations of homocysteine in antiepileptic drug treatment. *Epilepsia* **1999**, *40*, 345–350, doi:10.1111/j.1528-1157.1999.tb00716.x.

46. Apeland, T.; Mansoor, M.A.; Strandjord, R.E. Antiepileptic drugs as independent predictors of plasma total homocysteine levels. *Epilepsy Res.* **2001**, *47*, 27–35, doi:10.1016/s0920-1211(01)00288-1.

47. Linnebank, M.; Moskau, S.; Semmler, A.; Widman, G.; Stoffel-Wagner, B.; Weller, M.; Elger, C.E. Antiepileptic drugs interact with folate and vitamin B12 serum levels. *Ann. Neurol.* **2011**, *69*, 352–359, doi:10.1002/ana.22229.

48. Aycicek, A.; Iscan, A. The effects of carbamazepine, valproic acid and phenobarbital on the oxidative and antioxidative balance in epileptic children. *Eur. Neurol.* **2007**, *57*, 65–69, doi:10.1159/000098053.

49. Liu, J.; Huang, D.; Xu, J.; Tong, J.; Wang, Z.; Huang, L.; Yang, Y.; Bai, X.; Wang, P.; Suo, H.; Ma, Y.; Yu, M.; Fei, J.; Huang, F. Tiagabine Protects Dopaminergic Neurons against Neurotoxins by Inhibiting Microglial Activation. *Sci. Rep.* **2015**, *5*, 15720, doi:10.1038/srep15720.

50. Yang, Y.; Li, Q.; Wang, C.X.; Jeerakathil, T.; Shuaib, A. Dose-dependent neuroprotection with tiagabine in a focal cerebral ischemia model in rat. *Neuroreport* **2000**, *11*, 2307–2311, doi:10.1097/00001756-200007140-00048.

51. Chen Xu, W.; Yi, Y.; Qiu, L.; Shuaib, A. Neuroprotective activity of tiagabine in a focal embolic model of cerebral ischemia. *Brain Res.* **2000**, *874*, 75–77, doi:10.1016/s0006-8993(00)02554-3.

52. Javaid, S.; Alqahtani, F.; Ashraf, W.; Anjum, S.M.M.; Rasool, M.F.; Ahmad, T.; Alasmari, F.; Alasmari, A.F.; Alqarni, S.A.; Imran, I. Tiagabine suppresses pentylenetetrazole-induced seizures in mice and improves behavioral and cognitive parameters by modulating BDNF/TrkB expression and neuroinflammatory markers. *Biomed. Pharmacother.* **2023**, *160*, 114406, doi:10.1016/j.biopha.2023.114406.

53. Cardile, V.; Pavone, A.; Renis, M.; Russo, A.; Perciavalle, V. Biological effects of tiagabine on primary cortical astrocyte cultures of rat. *Neurosci. Lett.* **2000**, *288*, 49–52, doi:10.1016/s0304-3940(00)01206-4.

54. Raza, M.; Al-Shabanah, O.A.; El-Hadiyah, T.M.H.; Qureshi, S. Thermoregulatory and In-vivo Anti-inflammatory Effects of Vigabatrin In Rat and Mice. *Sci. Pharm.* **2000**, *68*, 379–388, doi:10.3797/scipharm.aut-00-35.

55. Durdag, E.; Yildirim, Z.; Unlu, N.L.; Kale, A.; Ceviker, N. Neuroprotective Effects of Vigabatrin on Spinal Cord Ischemia-Reperfusion Injury. *World Neurosurg.* **2018**, *120*, e33–e41, doi:10.1016/j.wneu.2018.07.103.

56. Westall, C.A.; Wright, T.; Cortese, F.; Kumarappah, A.; Snead, O.C.; Buncic, J.R. Vigabatrin retinal toxicity in children with infantile spasms: An observational cohort study. *Neurology* **2014**, *83*, 2262–2268, doi:10.1212/WNL.0000000000001069.

57. Rasmussen, A.D.; Truchot, N.; Dyssegaard, A.; Fant, P. Retinal and Systemic Toxicity of Vigabatrin Is Driven by the S-Enantiomer in the Long Evans Rat. *Toxicol. Pathol.* **2023**, *51*, 126–134, doi:10.1177/01926233231179147.

58. Yang, J.; Naumann, M.C.; Tsai, Y.-T.; Tosi, J.; Erol, D.; Lin, C.-S.; Davis, R.J.; Tsang, S.H. Vigabatrin-induced retinal toxicity is partially mediated by signaling in rod and cone photoreceptors. *PLoS ONE* **2012**, *7*, e43889, doi:10.1371/journal.pone.0043889.

59. Labh, R.; Gupta, R.; Narang, M.; Halder, S.; Kar, R. Effect of valproate and add-on levetiracetam on inflammatory biomarkers in children with epilepsy. *Epilepsy Behav.* **2021**, *125*, 108358, doi:10.1016/j.yebeh.2021.108358.

60. Verrotti, A.; Scardapane, A.; Franzoni, E.; Manco, R.; Chiarelli, F. Increased oxidative stress in epileptic children treated with valproic acid. *Epilepsy Res.* **2008**, *78*, 171–177, doi:10.1016/j.eplepsyres.2007.11.005.

61. Miller, S.L.; Bennet, L.; Sutherland, A.E.; Pham, Y.; McDonald, C.; Castillo-Melendez, M.; Allison, B.J.; Mihelakis, J.; Nitsos, I.; Boyd, B.J.; Hirst, J.J.; Walker, D.W.; Hunt, R.W.; Jenkin, G.; Wong, F.; Malhotra, A.; Fahey, M.C.; Yawno, T. Ganaxolone versus Phenobarbital for Neonatal Seizure Management. *Ann. Neurol.* **2022**, *92*, 1066–1079, doi:10.1002/ana.26493.

62. Mouihate, A.; Kalakh, S. Ganaxolone enhances microglial clearance activity and promotes remyelination in focal demyelination in the corpus callosum of ovariectomized rats. *CNS Neurosci. Ther.* **2020**, *26*, 240–250, doi:10.1111/cns.13195.

63. Divya; Faruq, M.; Nazir, S.S.; Kaushik, P.; Parvez, S.; Vohora, D. Ganaxolone Reverses the Effect of Amyloid β-Induced Neurotoxicity by Regulating the Liver X Receptor Expression in APP Transfected SH-SY5Y Cells and Murine Model of Alzheimer's Disease. *J. Neurochem.* **2025**, *169*, e70007, doi:10.1111/jnc.70007.

64. Balan, I.; Beattie, M.C.; O'Buckley, T.K.; Aurelian, L.; Morrow, A.L. Endogenous Neurosteroid (3α,5α)3-Hydroxypregnan-20-one Inhibits Toll-like-4 Receptor Activation and Pro-inflammatory Signaling in Macrophages and Brain. *Sci. Rep.* **2019**, *9*, 1220, doi:10.1038/s41598-018-37409-6.

65. Wang, T.; Chen, S.; Mao, Z.; Shang, Y.; Brinton, R.D. Allopregnanolone pleiotropic action in neurons and astrocytes: calcium signaling as a unifying mechanism. *Front Endocrinol (Lausanne)* **2023**, *14*, 1286931, doi:10.3389/fendo.2023.1286931.

66. Bayhan, I.; Turtay, M.G.; Ciftci, O.; Cetin, A.; Basak, N.; Namık Oztanır, M.; Oguzturk, H.; Gurbuz, S.; Guven, T. Comparison of immunological, histological and oxidative effects of felbamate and levetiracetam in traumatic brain injury. *Eur. Rev. Med. Pharmacol. Sci.* **2020**, *24*, 7083–7091, doi:10.26355/eurrev_202006_21702.

67. Wasterlain, C.G.; Adams, L.M.; Wichmann, J.K.; Sofia, R.D. Felbamate protects CA1 neurons from apoptosis in a gerbil model of global ischemia. *Stroke* **1996**, *27*, 1236–1240, doi:10.1161/01.str.27.7.1236.

68. Shuaib, A.; Waqaar, T.; Ijaz, M.S.; Kanthan, R.; Wishart, T.; Howlett, W. Neuroprotection with felbamate: a 7- and 28-day study in transient forebrain ischemia in gerbils. *Brain Res.* **1996**, *727*, 65–70, doi:10.1016/0006-8993(96)00352-6.

69. Thompson, C.D.; Gulden, P.H.; Macdonald, T.L. Identification of modified atropaldehyde mercapturic acids in rat and human urine after felbamate administration. *Chem. Res. Toxicol.* **1997**, *10*, 457–462, doi:10.1021/tx960205e.

70. Andrzejczak, D.; Woldan-Tambor, A.; Bednarska, K.; Zawilska, J.B. The effects of topiramate on lipopolysaccharide (LPS)-induced proinflammatory cytokine release from primary rat microglial cell cultures. *Epilepsy Res.* **2016**, *127*, 352–357, doi:10.1016/j.eplepsyres.2016.09.020.

71. Kutluhan, S.; Naziroğlu, M.; Celik, O.; Yilmaz, M. Effects of selenium and topiramate on lipid peroxidation and antioxidant vitamin levels in blood of pentylentetrazol-induced epileptic rats. *Biol. Trace Elem. Res.* **2009**, *129*, 181–189, doi:10.1007/s12011-008-8287-4.

72. Motaghinejad, M.; Motevalian, M.; Falak, R.; Heidari, M.; Sharzad, M.; Kalantari, E. Neuroprotective effects of various doses of topiramate against methylphenidate-induced oxidative stress and inflammation in isolated rat amygdala: the possible role of CREB/BDNF signaling pathway. *J. Neural Transm.* **2016**, *123*, 1463–1477, doi:10.1007/s00702-016-1619-1.

73. Pavone, A.; Cardile, V. An in vitro study of new antiepileptic drugs and astrocytes. *Epilepsia* **2003**, *44*, 34–39, doi:10.1046/j.1528-1157.44.s10.5.x.

74. Muriach, M.; López-Pedrajas, R.; Barcia, J.M.; Sanchez-Villarejo, M.V.; Almansa, I.; Romero, F.J. Cocaine causes memory and learning impairments in rats: involvement of nuclear factor kappa B and oxidative stress, and prevention by topiramate. *J. Neurochem.* **2010**, *114*, 675–684, doi:10.1111/j.1471-4159.2010.06794.x.

75. Demirci, S.; Kutluhan, S.; Naziroğlu, M.; Uğuz, A.C.; Yürekli, V.A.; Demirci, K. Effects of selenium and topiramate on cytosolic Ca(2+) influx and oxidative stress in neuronal PC12 cells. *Neurochem. Res.* **2013**, *38*, 90–97, doi:10.1007/s11064-012-0893-z.

76. Chen, T.; Dai, S.-H.; Jiang, Z.-Q.; Luo, P.; Jiang, X.-F.; Fei, Z.; Gui, S.-B.; Qi, Y.-L. The AMPAR Antagonist Perampanel Attenuates Traumatic Brain Injury Through Anti-Oxidative and Anti-Inflammatory Activity. *Cell. Mol. Neurobiol.* **2017**, *37*, 43–52, doi:10.1007/s10571-016-0341-8.

77. Yang, L.; Wang, Y.; Zhang, C.; Chen, T.; Cheng, H. Perampanel, an AMPAR antagonist, alleviates experimental intracerebral hemorrhage-induced brain injury via necroptosis and neuroinflammation. *Mol. Med. Report.* **2021**, *24*, doi:10.3892/mmr.2021.12183.

78. Niu, H.-X.; Wang, J.-Z.; Wang, D.-L.; Miao, J.-J.; Li, H.; Liu, Z.-G.; Yuan, X.; Liu, W.; Zhou, J.-R. The orally active noncompetitive AMPAR antagonist perampanel attenuates focal cerebral ischemia injury in rats. *Cell. Mol. Neurobiol.* **2018**, *38*, 459–466, doi:10.1007/s10571-017-0489-x.

79. Chen, T.; Liu, W.-B.; Qian, X.; Xie, K.-L.; Wang, Y.-H. The AMPAR antagonist perampanel protects the neurovascular unit against traumatic injury via regulating Sirt3. *CNS Neurosci. Ther.* **2021**, *27*, 134–144, doi:10.1111/cns.13580.

80. Shin, H.J.; Lee, K.Y.; Kang, J.W.; Choi, S.G.; Kim, D.W.; Yi, Y.Y. Perampanel reduces brain damage via induction of M2 microglia in a neonatal rat stroke model. *Int. J. Nanomedicine* **2022**, *17*, 2791–2804, doi:10.2147/IJN.S361377.

81. Yang, H.; Ding, C.; Cheng, M.; Sheng, Z.; Chen, L.; Chen, J.; Wang, Y. Perampanel attenuates oxidative stress and pyroptosis following subarachnoid hemorrhage via the SIRT3/FOXO3α pathway. *Sci. Rep.* **2023**, *13*, 21320, doi:10.1038/s41598-023-48802-1.

82. Kawakita, F.; Nakano, F.; Kanamaru, H.; Asada, R.; Suzuki, H. Anti-Apoptotic Effects of AMPA Receptor Antagonist Perampanel in Early Brain Injury After Subarachnoid Hemorrhage in Mice. *Transl. Stroke Res.* **2024**, *15*, 462–475, doi:10.1007/s12975-023-01138-4.

83. Lv, J.-M.; Pan, Y.-J.; Wang, X.; Zhang, M.-M.; Li, W.; Liu, J.; Wang, T. Perampanel regulates neuroinflammation and ferroptosis via activating FSP1 following brain ischemia. *J. Inflamm. Res.* **2025**, *18*, 14423–14437, doi:10.2147/JIR.S544785.

84. Kim, J.-E.; Choi, H.-C.; Song, H.-K.; Jo, S.-M.; Kim, D.-S.; Choi, S.-Y.; Kim, Y.-I.; Kang, T.-C. Levetiracetam inhibits interleukin-1 beta inflammatory responses in the hippocampus and piriform cortex of epileptic rats. *Neurosci. Lett.* **2010**, *471*, 94–99, doi:10.1016/j.neulet.2010.01.018.

85. Stienen, M.N.; Haghikia, A.; Dambach, H.; Thöne, J.; Wiemann, M.; Gold, R.; Chan, A.; Dermietzel, R.; Faustmann, P.M.; Hinkerohe, D.; Prochnow, N. Anti-inflammatory effects of the anticonvulsant drug levetiracetam on electrophysiological properties of astroglia are mediated via TGFβ1 regulation. *Br. J. Pharmacol.* **2011**, *162*, 491–507, doi:10.1111/j.1476-5381.2010.01038.x.

86. Itoh, K.; Ishihara, Y.; Komori, R.; Nochi, H.; Taniguchi, R.; Chiba, Y.; Ueno, M.; Takata-Tsuji, F.; Dohgu, S.; Kataoka, Y. Levetiracetam treatment influences blood-brain barrier failure associated with angiogenesis and inflammatory responses in the acute phase of epileptogenesis in post-status epilepticus mice. *Brain Res.* **2016**, *1652*, 1–13, doi:10.1016/j.brainres.2016.09.038.

87. Itoh, K.; Taniguchi, R.; Matsuo, T.; Oguro, A.; Vogel, C.F.A.; Yamazaki, T.; Ishihara, Y. Suppressive effects of levetiracetam on neuroinflammation and phagocytic microglia: A comparative study of levetiracetam, valproate and carbamazepine. *Neurosci. Lett.* **2019**, *708*, 134363, doi:10.1016/j.neulet.2019.134363.

88. Niidome, K.; Taniguchi, R.; Yamazaki, T.; Tsuji, M.; Itoh, K.; Ishihara, Y. Fosl1 is a novel target of levetiracetam for suppressing the microglial inflammatory reaction. *Int. J. Mol. Sci.* **2021**, *22*, doi:10.3390/ijms222010962.

89. Komori, R.; Matsuo, T.; Yokota-Nakatsuma, A.; Hashimoto, R.; Kubo, S.; Kozawa, C.; Kono, T.; Ishihara, Y.; Itoh, K. Regulation of Inflammation-Related Genes through Fosl1 Suppression in a Levetiracetam-Treated Pilocarpine-Induced Status Epilepticus Mouse Model. *Int. J. Mol. Sci.* **2022**, *23*, doi:10.3390/ijms23147608.

90. Matsuo, T.; Komori, R.; Nakatani, M.; Ochi, S.; Yokota-Nakatsuma, A.; Matsumoto, J.; Takata, F.; Dohgu, S.; Ishihara, Y.; Itoh, K. Levetiracetam Suppresses the Infiltration of Neutrophils and Monocytes and Downregulates Many Inflammatory Cytokines during Epileptogenesis in Pilocarpine-Induced Status Epilepticus Mice. *Int. J. Mol. Sci.* **2022**, *23*, doi:10.3390/ijms23147671.

91. Zhang, Y.-Y.; Wang, L.; Guo, H.; Han, T.-T.; Chang, Y.-H.; Cui, X.-C. Levetiracetam attenuates diabetes-associated cognitive impairment and microglia polarization by suppressing neuroinflammation. *Front. Pharmacol.* **2023**, *14*, 1145819, doi:10.3389/fphar.2023.1145819.

92. Oliveira, A.A.; Almeida, J.P.C.; Freitas, R.M.; Nascimento, V.S.; Aguiar, L.M.V.; Júnior, H.V.N.; Fonseca, F.N.; Viana, G.S.B.; Sousa, F.C.F.; Fonteles, M.M.F. Effects of levetiracetam in lipid peroxidation level, nitrite-nitrate formation and antioxidant enzymatic activity in mice brain after pilocarpine-induced seizures. *Cell. Mol. Neurobiol.* **2007**, *27*, 395–406, doi:10.1007/s10571-006-9132-y.

93. Ueda, Y.; Doi, T.; Takaki, M.; Nagatomo, K.; Nakajima, A.; Willmore, L.J. Levetiracetam enhances endogenous antioxidant in the hippocampus of rats: in vivo evaluation by brain microdialysis combined with ESR spectroscopy. *Brain Res.* **2009**, *1266*, 1–7, doi:10.1016/j.brainres.2009.02.040.

94. Ignacio-Mejía, I.; Contreras-García, I.J.; Mendoza-Torreblanca, J.G.; Medina-Campos, O.N.; Pedraza-Chaverri, J.; García-Cruz, M.E.; Romo-Mancillas, A.; Gómez-Manzo, S.; Bandala, C.; Sánchez-Mendoza, M.E.; Pichardo-Macías, L.A.; Cárdenas-Rodríguez, N. Evaluation of the antioxidant activity of levetiracetam in a temporal lobe epilepsy model. *Biomedicines* **2023**, *11*, doi:10.3390/biomedicines11030848.

95. Ignacio-Mejía, I.; Contreras-García, I.J.; Pichardo-Macías, L.A.; García-Cruz, M.E.; Ramírez Mendiola, B.A.; Bandala, C.; Medina-Campos, O.N.; Pedraza-Chaverri, J.; Cárdenas-Rodríguez, N.; Mendoza-Torreblanca, J.G. Effect of Levetiracetam on Oxidant-Antioxidant Activity during Long-Term Temporal Lobe Epilepsy in Rats. *Int. J. Mol. Sci.* **2024**, *25*, doi:10.3390/ijms25179313.

96. Tsymbalyuk, S.; Smith, M.; Gore, C.; Tsymbalyuk, O.; Ivanova, S.; Sansur, C.; Gerzanich, V.; Simard, J.M. Brivaracetam attenuates pain behaviors in a murine model of neuropathic pain. *Mol. Pain* **2019**, *15*, 1744806919886503, doi:10.1177/1744806919886503.

97. Deval, C.; Sharma, P.; Sharma, B.; Singh, B. Brivaracitam ameliorates increased inflammation, oxidative stress, and acetylcholinesterase activity in ischemic mice. *Clin. Psychopharmacol. Neurosci.* **2025**, *23*, 120–132, doi:10.9758/cpn.24.1216.

98. Okada, M.; Fukuyama, K.; Shiroyama, T.; Ueda, Y. Brivaracetam prevents astroglial l-glutamate release associated with hemichannel through modulation of synaptic vesicle protein. *Biomed. Pharmacother.* **2021**, *138*, 111462, doi:10.1016/j.biopha.2021.111462.

99. Fukuyama, K.; Okada, M. Brivaracetam and Levetiracetam Suppress Astroglial L-Glutamate Release through Hemichannel via Inhibition of Synaptic Vesicle Protein. *Int. J. Mol. Sci.* **2022**, *23*, doi:10.3390/ijms23094473.

100. Ismail, F.S.; Faustmann, P.M.; Kümmel, M.-L.; Förster, E.; Faustmann, T.J.; Corvace, F. Brivaracetam exhibits mild pro-inflammatory features in an in vitro astrocyte-microglia co-culture model of inflammation. *Front. Cell. Neurosci.* **2022**, *16*, 995861, doi:10.3389/fncel.2022.995861.

101. Kelemen, K.; Sárosi, M.; Csüdör, Á.; Orbán-Kis, K.; Kelemen, H.; Bába, L.; Gáll, Z.; Horváth, E.; Katona, I.; Szilágyi, T. Marked differences in the effects of levetiracetam and its analogue brivaracetam on microglial, astrocytic, and neuronal density in the rat model of kainic acid-induced temporal lobe epilepsy. *Front. Pharmacol.* **2025**, *16*, 1553545, doi:10.3389/fphar.2025.1553545.

102. Liou, G.I.; Auchampach, J.A.; Hillard, C.J.; Zhu, G.; Yousufzai, B.; Mian, S.; Khan, S.; Khalifa, Y. Mediation of cannabidiol anti-inflammation in the retina by equilibrative nucleoside transporter and A2A adenosine receptor. *Invest. Ophthalmol. Vis. Sci.* **2008**, *49*, 5526–5531, doi:10.1167/iovs.08-2196.

103. Hayakawa, K.; Mishima, K.; Irie, K.; Hazekawa, M.; Mishima, S.; Fujioka, M.; Orito, K.; Egashira, N.; Katsurabayashi, S.; Takasaki, K.; Iwasaki, K.; Fujiwara, M. Cannabidiol prevents a post-ischemic injury progressively induced by cerebral ischemia via a high-mobility group box1-inhibiting mechanism. *Neuropharmacology* **2008**, *55*, 1280–1286, doi:10.1016/j.neuropharm.2008.06.040.

104. Hayakawa, K.; Irie, K.; Sano, K.; Watanabe, T.; Higuchi, S.; Enoki, M.; Nakano, T.; Harada, K.; Ishikane, S.; Ikeda, T.; Fujioka, M.; Orito, K.; Iwasaki, K.; Mishima, K.; Fujiwara, M. Therapeutic time window of cannabidiol treatment on delayed ischemic damage via high-mobility group box1-inhibiting mechanism. *Biol. Pharm. Bull.* **2009**, *32*, 1538–1544, doi:10.1248/bpb.32.1538.

105. Cassol, O.J.; Comim, C.M.; Silva, B.R.; Hermani, F.V.; Constantino, L.S.; Felisberto, F.; Petronilho, F.; Hallak, J.E.C.; De Martinis, B.S.; Zuardi, A.W.; Crippa, J.A.S.; Quevedo, J.; Dal-Pizzol, F. Treatment with cannabidiol reverses oxidative stress parameters, cognitive impairment and mortality in rats submitted to sepsis by cecal ligation and puncture. *Brain Res.* **2010**, *1348*, 128–138, doi:10.1016/j.brainres.2010.06.023.

106. Mukhopadhyay, P.; Rajesh, M.; Horváth, B.; Bátkai, S.; Park, O.; Tanchian, G.; Gao, R.Y.; Patel, V.; Wink, D.A.; Liaudet, L.; Haskó, G.; Mechoulam, R.; Pacher, P. Cannabidiol protects against hepatic ischemia/reperfusion injury by attenuating inflammatory signaling and response, oxidative/nitrative stress, and cell death. *Free Radic. Biol. Med.* **2011**, *50*, 1368–1381, doi:10.1016/j.freeradbiomed.2011.02.021.

107. Hosseinzadeh, M.; Nikseresht, S.; Khodagholi, F.; Naderi, N.; Maghsoudi, N. Cannabidiol Post-Treatment Alleviates Rat Epileptic-Related Behaviors and Activates Hippocampal Cell Autophagy Pathway Along with Antioxidant Defense in Chronic Phase of Pilocarpine-Induced Seizure. *J. Mol. Neurosci.* **2016**, *58*, 432–440, doi:10.1007/s12031-015-0703-6.

108. Mori, M.A.; Meyer, E.; Soares, L.M.; Milani, H.; Guimarães, F.S.; de Oliveira, R.M.W. Cannabidiol reduces neuroinflammation and promotes neuroplasticity and functional recovery after brain ischemia. *Prog Neuropsychopharmacol Biol Psychiatry* **2017**, *75*, 94–105, doi:10.1016/j.pnpbp.2016.11.005.

109. Ceprián, M.; Jiménez-Sánchez, L.; Vargas, C.; Barata, L.; Hind, W.; Martínez-Orgado, J. Cannabidiol reduces brain damage and improves functional recovery in a neonatal rat model of arterial ischemic stroke. *Neuropharmacology* **2017**, *116*, 151–159, doi:10.1016/j.neuropharm.2016.12.017.

110. Sun, S.; Hu, F.; Wu, J.; Zhang, S. Cannabidiol attenuates OGD/R-induced damage by enhancing mitochondrial bioenergetics and modulating glucose metabolism via pentose-phosphate pathway in hippocampal neurons. *Redox Biol.* **2017**, *11*, 577–585, doi:10.1016/j.redox.2016.12.029.

111. Drummond-Main, C.D.; Ahn, Y.; Kesler, M.; Gavrilovici, C.; Kim, D.Y.; Kiroski, I.; Baglot, S.L.; Chen, A.; Sharkey, K.A.; Hill, M.N.; Teskey, G.C.; Rho, J.M. Cannabidiol impairs brain mitochondrial metabolism and neuronal integrity. *Cannabis Cannabinoid Res.* **2023**, *8*, 283–298, doi:10.1089/can.2022.0011.

112. Verleye, M.; Buttigieg, D.; Steinschneider, R. Neuroprotective activity of stiripentol with a possible involvement of voltage-dependent calcium and sodium channels. *J. Neurosci. Res.* **2016**, *94*, 179–189, doi:10.1002/jnr.23688.

113. Shin, M.C.; Lee, T.-K.; Lee, J.-C.; Kim, H.I.; Park, C.W.; Cho, J.H.; Kim, D.W.; Ahn, J.H.; Won, M.-H.; Lee, C.-H. Therapeutic effects of stiripentol against ischemia-reperfusion injury in gerbils focusing on cognitive deficit, neuronal death, astrocyte damage and blood brain barrier leakage in the hippocampus. *Korean J. Physiol. Pharmacol.* **2022**, *26*, 47–57, doi:10.4196/kjpp.2022.26.1.47.

114. Platow, R.J.; Pommer, S.; Brauer, J.; Wang, Y.; Mani, S.; Kaindl, A.M. Microglial activation is inhibited by selective anti-seizure medications. *Inflamm. Res.* **2025**, *74*, 112, doi:10.1007/s00011-025-02076-7.

115. Connor, T.J.; Kelly, J.P. Fenfluramine-induced immunosuppression: an in vivo analysis. *Eur. J. Pharmacol.* **2002**, *455*, 175–185, doi:10.1016/s0014-2999(02)02588-8.

116. Nakagawa, Y.; Suzuki, T.; Kamimura, H.; Nagai, F. N-Nitrosofenfluramine induces cytotoxicity via mitochondrial dysfunction and oxidative stress in isolated rat hepatocytes. *Arch. Toxicol.* **2005**, *79*, 312–320, doi:10.1007/s00204-004-0635-3.

117. Nakagawa, Y.; Suzuki, T.; Kamimura, H.; Nagai, F. Role of mitochondrial membrane permeability transition in N-nitrosofenfluramine-induced cell injury in rat hepatocytes. *Eur. J. Pharmacol.* **2006**, *529*, 33–39, doi:10.1016/j.ejphar.2005.10.042.

118. Cha, J.; Filatov, G.; Smith, S.J.; Gammaitoni, A.R.; Lothe, A.; Reeder, T. Fenfluramine increases survival and reduces markers of neurodegeneration in a mouse model of Dravet syndrome. *Epilepsia Open* **2024**, *9*, 300–313, doi:10.1002/epi4.12873.

119. Oliveira, M.S.; Furian, A.F.; Royes, L.F.F.; Fighera, M.R.; Fiorenza, N.G.; Castelli, M.; Machado, P.; Bohrer, D.; Veiga, M.; Ferreira, J.; Cavalheiro, E.A.; Mello, C.F. Cyclooxygenase-2/PGE2 pathway facilitates pentylenetetrazol-induced seizures. *Epilepsy Res.* **2008**, *79*, 14–21, doi:10.1016/j.eplepsyres.2007.12.008.

120. Zandieh, A.; Maleki, F.; Hajimirzabeigi, A.; Zandieh, B.; Khalilzadeh, O.; Dehpour, A.R. Anticonvulsant effect of celecoxib on pentylenetetrazole-induced convulsion: Modulation by NO pathway. *Acta Neurobiol Exp (Wars)* **2010**, *70*, 390–397, doi:10.55782/ane-2010-1811.

121. Zhang, H.-J.; Sun, R.-P.; Lei, G.-F.; Yang, L.; Liu, C.-X. Cyclooxygenase-2 inhibitor inhibits hippocampal synaptic reorganization in pilocarpine-induced status epilepticus rats. *J. Zhejiang Univ. Sci. B* **2008**, *9*, 903–915, doi:10.1631/jzus.B0820018.

122. Zhou, L.; Zhou, L.; Su, L.; Cao, S.-L.; Xie, Y.-J.; Wang, N.; Shao, C.-Y.; Wang, Y.-N.; Zhou, J.-H.; Cowell, J.K.; Shen, Y. Celecoxib ameliorates seizure susceptibility in autosomal dominant lateral temporal epilepsy. *J. Neurosci.* **2018**, *38*, 3346–3357, doi:10.1523/JNEUROSCI.3245-17.2018.

123. Akula, K.K.; Dhir, A.; Kulkarni, S.K. Rofecoxib, a selective cyclooxygenase-2 (COX-2) inhibitor increases pentylenetetrazol seizure threshold in mice: possible involvement of adenosinergic mechanism. *Epilepsy Res.* **2008**, *78*, 60–70, doi:10.1016/j.eplepsyres.2007.10.008.

124. Claycomb, R.J.; Hewett, S.J.; Hewett, J.A. Prophylactic, prandial rofecoxib treatment lacks efficacy against acute PTZ-induced seizure generation and kindling acquisition. *Epilepsia* **2011**, *52*, 273–283, doi:10.1111/j.1528-1167.2010.02889.x.

125. Citraro, R.; Leo, A.; Marra, R.; De Sarro, G.; Russo, E. Antiepileptogenic effects of the selective COX-2 inhibitor etoricoxib, on the development of spontaneous absence seizures in WAG/Rij rats. *Brain Res. Bull.* **2015**, *113*, 1–7, doi:10.1016/j.brainresbull.2015.02.004.

126. Katyal, J.; Kumar, H.; Gupta, Y.K. Anticonvulsant activity of the cyclooxygenase-2 (COX-2) inhibitor etoricoxib in pentylenetetrazole-kindled rats is associated with memory impairment. *Epilepsy Behav.* **2015**, *44*, 98–103, doi:10.1016/j.yebeh.2014.12.032.

127. Jeong, K.H.; Kim, J.Y.; Choi, Y.-S.; Lee, M.-Y.; Kim, S.Y. Influence of aspirin on pilocarpine-induced epilepsy in mice. *Korean J. Physiol. Pharmacol.* **2013**, *17*, 15–21, doi:10.4196/kjpp.2013.17.1.15.

128. Ma, L.; Cui, X.-L.; Wang, Y.; Li, X.-W.; Yang, F.; Wei, D.; Jiang, W. Aspirin attenuates spontaneous recurrent seizures and inhibits hippocampal neuronal loss, mossy fiber sprouting and aberrant neurogenesis following pilocarpine-induced status epilepticus in rats. *Brain Res.* **2012**, *1469*, 103–113, doi:10.1016/j.brainres.2012.05.058.

129. Barbalho, P.G.; Lopes-Cendes, I.; Maurer-Morelli, C.V. Indomethacin treatment prior to pentylenetetrazole-induced seizures downregulates the expression of il1b and cox2 and decreases seizure-like behavior in zebrafish larvae. *BMC Neurosci.* **2016**, *17*, 12, doi:10.1186/s12868-016-0246-y.

130. Liu, R.; Wu, S.; Guo, C.; Hu, Z.; Peng, J.; Guo, K.; Zhang, X.; Li, J. Ibuprofen Exerts Antiepileptic and Neuroprotective Effects in the Rat Model of Pentylenetetrazol-Induced Epilepsy via the COX-2/NLRP3/IL-18 Pathway. *Neurochem. Res.* **2020**, *45*, 2516–2526, doi:10.1007/s11064-020-03109-9.

131. Durankuş, F.; Şenkal, E.; Sünnetçi, E.; Albayrak, Y.; Beyazyüz, M.; Atasoy, Ö.; Erbaş, O. Beneficial Effects of Ibuprofen on Pentylenetetrazol-induced Convulsion. *Neurochem. Res.* **2020**, *45*, 2409–2416, doi:10.1007/s11064-020-03101-3.

132. Samriti; Kaur, A.; Kaur, A.; Goel, R.K. Ameliorative effect of diclofenac in rotenone corneal kindling model of drug-resistant epilepsy: Edge of dual COX and KMO inhibition. *Brain Res.* **2025**, *1846*, 149246, doi:10.1016/j.brainres.2024.149246.

133. Vieira, V.; Glassmann, D.; Marafon, P.; Pereira, P.; Gomez, R.; Coitinho, A.S. Effect of diclofenac sodium on seizures and inflammatory profile induced by kindling seizure model. *Epilepsy Res.* **2016**, *127*, 107–113, doi:10.1016/j.eplepsyres.2016.08.020.

134. van Stuijvenberg, M.; Derksen-Lubsen, G.; Steyerberg, E.W.; Habbema, J.D.; Moll, H.A. Randomized, controlled trial of ibuprofen syrup administered during febrile illnesses to prevent febrile seizure recurrences. *Pediatrics* **1998**, *102*, E51, doi:10.1542/peds.102.5.e51.

135. Strengell, T.; Uhari, M.; Tarkka, R.; Uusimaa, J.; Alen, R.; Lautala, P.; Rantala, H. Antipyretic agents for preventing recurrences of febrile seizures: randomized controlled trial. *Arch. Pediatr. Adolesc. Med.* **2009**, *163*, 799–804, doi:10.1001/archpediatrics.2009.137.

136. Metcalf, C.S.; Vanegas, F.; Underwood, T.; Johnson, K.; West, P.J.; Smith, M.D.; Wilcox, K.S. Screening of prototype antiseizure and anti-inflammatory compounds in the Theiler's murine encephalomyelitis virus model of epilepsy. *Epilepsia Open* **2022**, *7*, 46–58, doi:10.1002/epi4.12550.

137. Garcia-Curran, M.M.; Hall, A.M.; Patterson, K.P.; Shao, M.; Eltom, N.; Chen, K.; Dubé, C.M.; Baram, T.Z. Dexamethasone attenuates hyperexcitability provoked by experimental febrile status epilepticus. *eNeuro* **2019**, *6*, doi:10.1523/ENEURO.0430-19.2019.

138. Shi, X.-Y.; Yang, X.-F.; Tomonoh, Y.; Hu, L.-Y.; Ju, J.; Hirose, S.; Zou, L.-P. Development of a mouse model of infantile spasms induced by N-methyl-D-aspartate. *Epilepsy Res.* **2015**, *118*, 29–33, doi:10.1016/j.eplepsyres.2015.09.014.

139. Le, J.T.; Frost, J.D.; Swann, J.W. Acthar® Gel (repository corticotropin injection) dose-response relationships in an animal model of epileptic spasms. *Epilepsy Behav.* **2021**, *116*, 107786, doi:10.1016/j.yebeh.2021.107786.

140. Fox, P.; Mithal, D.S.; Somogyi, J.R.; Vien, A.C.; Sanchez, R.M.; Koh, S. Dexamethasone after early-life seizures attenuates increased susceptibility to seizures, seizure-induced microglia activation and neuronal injury later in life. *Neurosci. Lett.* **2020**, *728*, 134953, doi:10.1016/j.neulet.2020.134953.

141. Sorel, L.; Dusaucy-Bauloye, A. [Findings in 21 cases of Gibbs' hypsarrhythmia; spectacular effectiveness of ACTH]. *Acta Neurol. Psychiatr. Belg.* **1958**, *58*, 130–141.

142. Falsaperla, R.; Collotta, A.D.; Marino, S.D.; Sortino, V.; Leonardi, R.; Privitera, G.F.; Pulvirenti, A.; Suppiej, A.; Vecchi, M.; Verrotti, A.; Farello, G.; Spalice, A.; Elia, M.; Spitaleri, O.; Micale, M.; Mailo, J.; Ruggieri, M. Drug resistant epilepsies: A multicentre case series of steroid therapy. *Seizure* **2024**, *117*, 115–125, doi:10.1016/j.seizure.2024.02.007.

143. Schiller, K.; Thomas, J.; Avigdor, T.; Mansilla, D.; Kortas, A.; Unterholzner, G.; Rauchenzauner, M.; Frauscher, B. Pulsatile corticoid therapy reduces interictal epileptic activity burden in children with genetic drug-resistant epilepsy. *Epilepsia Open* **2024**, *9*, 1265–1276, doi:10.1002/epi4.12947.

144. Byun, J.-I.; Lee, S.-T.; Jung, K.-H.; Sunwoo, J.-S.; Moon, J.; Lim, J.-A.; Lee, D.Y.; Shin, Y.-W.; Kim, T.-J.; Lee, K.-J.; Lee, W.-J.; Lee, H.-S.; Jun, J.; Kim, D.-Y.; Kim, M.-Y.; Kim, H.; Kim, H.J.; Suh, H.I.; Lee, Y.; Kim, D.W.; Jeong, J.H.; Choi, W.C.; Bae, D.W.; Shin, J.-W.; Jeon, D.; Park, K.-I.; Jung, K.-Y.; Chu, K.; Lee, S.K. Effect of Immunotherapy on Seizure Outcome in Patients with Autoimmune Encephalitis: A Prospective Observational Registry Study. *PLoS ONE* **2016**, *11*, e0146455, doi:10.1371/journal.pone.0146455.

145. Sinclair, D.B. Prednisone therapy in pediatric epilepsy. *Pediatr. Neurol.* **2003**, *28*, 194–198, doi:10.1016/s0887-8994(02)00513-1.

146. Verhelst, H.; Boon, P.; Buyse, G.; Ceulemans, B.; D'Hooghe, M.; Meirleir, L.D.; Hasaerts, D.; Jansen, A.; Lagae, L.; Meurs, A.; Coster, R.V.; Vonck, K. Steroids in intractable childhood epilepsy: clinical experience and review of the literature. *Seizure* **2005**, *14*, 412–421, doi:10.1016/j.seizure.2005.07.002.

147. Inutsuka, M.; Kobayashi, K.; Oka, M.; Hattori, J.; Ohtsuka, Y. Treatment of epilepsy with electrical status epilepticus during slow sleep and its related disorders. *Brain Dev.* **2006**, *28*, 281–286, doi:10.1016/j.braindev.2005.09.004.

148. van Arnhem, M.M.L.; van den Munckhof, B.; Arzimanoglou, A.; Perucca, E.; Metsähonkala, L.; Rubboli, G.; Søndergaard Khinchi, M.; de Saint-Martin, A.; Klotz, K.A.; Jacobs, J.; Cross, J.H.; Garcia Morales, I.; Otte, W.M.; van Teeseling, H.C.; Leijten, F.S.S.; Braun, K.P.J.; Jansen, F.E.; RESCUE ESES study group Corticosteroids versus clobazam for treatment of children with epileptic encephalopathy with spike-wave activation in sleep (RESCUE ESES): a multicentre randomised controlled trial. *Lancet Neurol.* **2024**, *23*, 147–156, doi:10.1016/S1474-4422(23)00409-X.

149. Sevilla-Castillo, R.A.; Palacios, G.C.; Ramirez-Campos, J.; Mora-Puga, M.; Diaz-Bustos, R. Methylprednisolone for the treatment of children with refractory epilepsy. *Neuropediatrics* **2009**, *40*, 265–268, doi:10.1055/s-0030-1249653.

150. Almaabdi, K.H.; Alshehri, R.O.; Althubiti, A.A.; Alsharef, Z.H.; Mulla, S.N.; Alshaer, D.S.; Alfaidi, N.S.; Jan, M.M. Intravenous methylprednisolone for intractable childhood epilepsy. *Pediatr. Neurol.* **2014**, *50*, 334–336, doi:10.1016/j.pediatrneurol.2013.12.015.

151. Sinclair, D.B.; Snyder, T.J. Corticosteroids for the treatment of Landau-kleffner syndrome and continuous spike-wave discharge during sleep. *Pediatr. Neurol.* **2005**, *32*, 300–306, doi:10.1016/j.pediatrneurol.2004.12.006.

152. Wang, D.D.; Englot, D.J.; Garcia, P.A.; Lawton, M.T.; Young, W.L. Minocycline- and tetracycline-class antibiotics are protective against partial seizures in vivo. *Epilepsy Behav.* **2012**, *24*, 314–318, doi:10.1016/j.yebeh.2012.03.035.

153. Beheshti Nasr, S.M.; Moghimi, A.; Mohammad-Zadeh, M.; Shamsizadeh, A.; Noorbakhsh, S.M. The effect of minocycline on seizures induced by amygdala kindling in rats. *Seizure* **2013**, *22*, 670–674, doi:10.1016/j.seizure.2013.05.005.

154. Ahmadirad, N.; Shojaei, A.; Javan, M.; Pourgholami, M.H.; Mirnajafi-Zadeh, J. Effect of minocycline on pentylenetetrazol-induced chemical kindled seizures in mice. *Neurol. Sci.* **2014**, *35*, 571–576, doi:10.1007/s10072-013-1552-0.

155. Wang, N.; Mi, X.; Gao, B.; Gu, J.; Wang, W.; Zhang, Y.; Wang, X. Minocycline inhibits brain inflammation and attenuates spontaneous recurrent seizures following pilocarpine-induced status epilepticus. *Neuroscience* **2015**, *287*, 144–156, doi:10.1016/j.neuroscience.2014.12.021.

156. Barker-Haliski, M.L.; Heck, T.D.; Dahle, E.J.; Vanegas, F.; Pruess, T.H.; Wilcox, K.S.; White, H.S. Acute treatment with minocycline, but not valproic acid, improves long-term behavioral outcomes in the Theiler's virus model of temporal lobe epilepsy. *Epilepsia* **2016**, *57*, 1958–1967, doi:10.1111/epi.13577.

157. Kovács, Z.; Kékesi, K.A.; Baracskay, P.; Juhász, G.; Czurkó, A. Doxycycline could aggravate the absence-like epileptic seizures of WAG/Rij rats via matrix metalloproteinase inhibition. *Neurochem. Int.* **2011**, *59*, 563–566, doi:10.1016/j.neuint.2011.06.016.

158. Russmann, V.; Goc, J.; Boes, K.; Ongerth, T.; Salvamoser, J.D.; Siegl, C.; Potschka, H. Minocycline fails to exert antiepileptogenic effects in a rat status epilepticus model. *Eur. J. Pharmacol.* **2016**, *771*, 29–39, doi:10.1016/j.ejphar.2015.12.002.

159. Nowak, M.; Strzelczyk, A.; Reif, P.S.; Schorlemmer, K.; Bauer, S.; Norwood, B.A.; Oertel, W.H.; Rosenow, F.; Strik, H.; Hamer, H.M. Minocycline as potent anticonvulsant in a patient with astrocytoma and drug resistant epilepsy. *Seizure* **2012**, *21*, 227–228, doi:10.1016/j.seizure.2011.12.009.

160. Feng, L.; Li, H.; Ma, L.; Hu, M.; Hui, B.; Sun, Z.; Wang, X.; Wang, Y.; Jiang, W. Minocycline in chronic management of febrile infection-related epilepsy syndrome (FIRES): a case series and literature review of treatment strategies. *Acta Epileptologica* **2025**, *7*, 35, doi:10.1186/s42494-025-00224-4.

161. Dyomina, A.V.; Zubareva, O.E.; Smolensky, I.V.; Vasilev, D.S.; Zakharova, M.V.; Kovalenko, A.A.; Schwarz, A.P.; Ischenko, A.M.; Zaitsev, A.V. Anakinra Reduces Epileptogenesis, Provides Neuroprotection, and Attenuates Behavioral Impairments in Rats in the Lithium-Pilocarpine Model of Epilepsy. *Pharmaceuticals (Basel)* **2020**, *13*, doi:10.3390/ph13110340.

162. Zubareva, O.E.; Sinyak, D.S.; Kalita, A.D.; Griflyuk, A.V.; Diespirov, G.P.; Postnikova, T.Y.; Zaitsev, A.V. Antiepileptogenic Effects of Anakinra, Lamotrigine and Their Combination in a Lithium-Pilocarpine Model of Temporal Lobe Epilepsy in Rats. *Int. J. Mol. Sci.* **2023**, *24*, doi:10.3390/ijms242015400.

163. Noe, F.M.; Polascheck, N.; Frigerio, F.; Bankstahl, M.; Ravizza, T.; Marchini, S.; Beltrame, L.; Banderó, C.R.; Löscher, W.; Vezzani, A. Pharmacological blockade of IL-1β/IL-1 receptor type 1 axis during epileptogenesis provides neuroprotection in two rat models of temporal lobe epilepsy. *Neurobiol. Dis.* **2013**, *59*, 183–193, doi:10.1016/j.nbd.2013.07.015.

164. Kenney-Jung, D.L.; Vezzani, A.; Kahoud, R.J.; LaFrance-Corey, R.G.; Ho, M.-L.; Muskardin, T.W.; Wirrell, E.C.; Howe, C.L.; Payne, E.T. Febrile infection-related epilepsy syndrome treated with anakinra. *Ann. Neurol.* **2016**, *80*, 939–945, doi:10.1002/ana.24806.

165. Lai, Y.-C.; Muscal, E.; Wells, E.; Shukla, N.; Eschbach, K.; Hyeong Lee, K.; Kaliakatsos, M.; Desai, N.; Wickström, R.; Viri, M.; Freri, E.; Granata, T.; Nangia, S.; Dilena, R.; Brunklaus, A.; Wainwright, M.S.; Gorman, M.P.; Stredny, C.M.; Asiri, A.; Hundallah, K.; Doja, A.; Payne, E.; Wirrell, E.; Koh, S.; Carpenter, J.L.; Riviello, J. Anakinra usage in febrile infection related epilepsy syndrome: an international cohort. *Ann. Clin. Transl. Neurol.* **2020**, *7*, 2467–2474, doi:10.1002/acn3.51229.

166. Mochol, M.; Taubøll, E.; Sveberg, L.; Tennøe, B.; Berg Olsen, K.; Heuser, K.; Svalheim, S. Seizure control after late introduction of anakinra in a patient with adult onset Rasmussen's encephalitis. *Epilepsy Behav. Rep.* **2021**, *16*, 100462, doi:10.1016/j.ebr.2021.100462.

167. DeSena, A.D.; Do, T.; Schulert, G.S. Systemic autoinflammation with intractable epilepsy managed with interleukin-1 blockade. *J. Neuroinflammation* **2018**, *15*, 38, doi:10.1186/s12974-018-1063-2.

168. Leo, A.; Nesci, V.; Tallarico, M.; Amodio, N.; Gallo Cantafio, E.M.; De Sarro, G.; Constanti, A.; Russo, E.; Citraro, R. IL-6 Receptor Blockade by Tocilizumab Has Anti-absence and Anti-epileptogenic Effects in the WAG/Rij Rat Model of Absence Epilepsy. *Neurotherapeutics* **2020**, *17*, 2004–2014, doi:10.1007/s13311-020-00893-8.

169. Jun, J.-S.; Lee, S.-T.; Kim, R.; Chu, K.; Lee, S.K. Tocilizumab treatment for new onset refractory status epilepticus. *Ann. Neurol.* **2018**, *84*, 940–945, doi:10.1002/ana.25374.

170. Stredny, C.M.; Case, S.; Sansevere, A.J.; Son, M.; Henderson, L.; Gorman, M.P. Interleukin-6 Blockade With Tocilizumab in Anakinra-Refractory Febrile Infection-Related Epilepsy Syndrome (FIRES). *Child Neurology Open* **2020**, *7*, 2329048X20979253, doi:10.1177/2329048X20979253.

171. Aledo-Serrano, A.; Hariramani, R.; Gonzalez-Martinez, A.; Álvarez-Troncoso, J.; Toledano, R.; Bayat, A.; Garcia-Morales, I.; Becerra, J.L.; Villegas-Martínez, I.; Beltran-Corbellini, A.; Gil-Nagel, A. Anakinra and tocilizumab in the chronic phase of febrile infection-related epilepsy syndrome (FIRES): Effectiveness and safety from a case-series. *Seizure* **2022**, *100*, 51–55, doi:10.1016/j.seizure.2022.06.012.

172. Lagarde, S.; Villeneuve, N.; Trébuchon, A.; Kaphan, E.; Lepine, A.; McGonigal, A.; Roubertie, A.; Barthez, M.-A.J.; Trommsdorff, V.; Lefranc, J.; Wehbi, S.; des Portes, V.; Laguitton, V.; Quartier, P.; Scavarda, D.; Giusiano, B.; Milh, M.; Bulteau, C.; Bartolomei, F. Anti-tumor necrosis factor alpha therapy (adalimumab) in Rasmussen's encephalitis: An open pilot study. *Epilepsia* **2016**, *57*, 956–966, doi:10.1111/epi.13387.

173. Maroso, M.; Balosso, S.; Ravizza, T.; Iori, V.; Wright, C.I.; French, J.; Vezzani, A. Interleukin-1β biosynthesis inhibition reduces acute seizures and drug resistant chronic epileptic activity in mice. *Neurotherapeutics* **2011**, *8*, 304–315, doi:10.1007/s13311-011-0039-z.

174. Cevik, B.; Solmaz, V.; Aksoy, D.; Erbas, O. Montelukast inhibits pentylenetetrazol-induced seizures in rats. *Med. Sci. Monit.* **2015**, *21*, 869–874, doi:10.12659/MSM.892932.

175. Lenz, Q.F.; Arroyo, D.S.; Temp, F.R.; Poersch, A.B.; Masson, C.J.; Jesse, A.C.; Marafiga, J.R.; Reschke, C.R.; Iribarren, P.; Mello, C.F. Cysteinyl leukotriene receptor (CysLT) antagonists decrease pentylenetetrazol-induced seizures and blood-brain barrier dysfunction. *Neuroscience* **2014**, *277*, 859–871, doi:10.1016/j.neuroscience.2014.07.058.

176. Fleck, J.; Temp, F.R.; Marafiga, J.R.; Jesse, A.C.; Milanesi, L.H.; Rambo, L.M.; Mello, C.F. Montelukast reduces seizures in pentylenetetrazol-kindled mice. *Braz. J. Med. Biol. Res.* **2016**, *49*, e5031, doi:10.1590/1414-431X20155031.

177. Fleck, J.; Marafiga, J.R.; Jesse, A.C.; Ribeiro, L.R.; Rambo, L.M.; Mello, C.F. Montelukast potentiates the anticonvulsant effect of phenobarbital in mice: an isobolographic analysis. *Pharmacol. Res.* **2015**, *94*, 34–41, doi:10.1016/j.phrs.2015.02.001.

178. Rehni, A.K.; Singh, T.G. Modulation of leukotriene D4 attenuates the development of seizures in mice. *Prostaglandins Leukot. Essent. Fatty Acids* **2011**, *85*, 97–106, doi:10.1016/j.plefa.2011.04.003.

179. Gao, F.; Liu, Y.; Li, X.; Wang, Y.; Wei, D.; Jiang, W. Fingolimod (FTY720) inhibits neuroinflammation and attenuates spontaneous convulsions in lithium-pilocarpine induced status epilepticus in rat model. *Pharmacol. Biochem. Behav.* **2012**, *103*, 187–196, doi:10.1016/j.pbb.2012.08.025.

180. Leo, A.; Citraro, R.; Amodio, N.; De Sarro, C.; Gallo Cantafio, M.E.; Constanti, A.; De Sarro, G.; Russo, E. Fingolimod Exerts only Temporary Antiepileptogenic Effects but Longer-Lasting Positive Effects on Behavior in the WAG/Rij Rat Absence Epilepsy Model. *Neurotherapeutics* **2017**, *14*, 1134–1147, doi:10.1007/s13311-017-0550-y.

181. Gao, F.; Gao, Y.; Meng, F.; Yang, C.; Fu, J.; Li, Y. The Sphingosine 1-Phosphate Analogue FTY720 Alleviates Seizure-induced Overexpression of P-Glycoprotein in Rat Hippocampus. *Basic Clin. Pharmacol. Toxicol.* **2018**, *123*, 14–20, doi:10.1111/bcpt.12973.

182. Türel, C.A.; Çelik, H.; Torun, İ.E.; Çetinkaya, A.; Türel, İ. The antiinflammatory and electrophysiological effects of fingolimod on penicillin-induced rats. *Arq Neuropsiquiatr* **2022**, *80*, 1220–1226, doi:10.1055/s-0042-1758754.

183. Garg, N.; Joshi, R.; Bhatia, A.; Bansal, S.; Chakrabarti, A.; Prakash, A.; Saikia, B.; Modi, M.; Medhi, B. Study of fingolimod, nitric oxide inhibitor, and P-glycoprotein inhibitor in modulating the P-glycoprotein expression via an endothelin-sphingolipid pathway in an animal model of pharmacoresistant epilepsy. *Indian J. Pharmacol.* **2023**, *55*, 307–314, doi:10.4103/ijp.ijp_100_23.

184. Bilister Egilmez, C.; Azak Pazarlar, B.; Erdogan, M.A.; Erbas, O. N-acetyl cysteine: A new look at its effect on PTZ-induced convulsions. *Epilepsy Res.* **2023**, *193*, 107144, doi:10.1016/j.eplepsyres.2023.107144.

185. Zaeri, S.; Emamghoreishi, M. Acute and Chronic Effects of N-acetylcysteine on Pentylenetetrazole-induced Seizure and Neuromuscular Coordination in Mice. *Iran. J. Med. Sci.* **2015**, *40*, 118–124.

186. Mohammadi, E.; Nikbakht, F.; Vazifekhah, S.; Babae, J.F.; Jogataei, M.T. Evaluation the cognition-improvement effects of N-acetyl cysteine in experimental temporal lobe epilepsy in rat. *Behav. Brain Res.* **2023**, *440*, 114263, doi:10.1016/j.bbr.2022.114263.

187. Mohammadi, E.; Nikbakht, F.; Barati, M.; Roghani, M.; Vazifekhah, S.; Khanizadeh, A.M.; Heidari, Z. Protective effect of N-acetyl cysteine on the mitochondrial dynamic imbalance in temporal lobe epilepsy: Possible role of mTOR. *Neuropeptides* **2022**, *96*, 102294, doi:10.1016/j.npep.2022.102294.

188. Pauletti, A.; Terrone, G.; Shekh-Ahmad, T.; Salamone, A.; Ravizza, T.; Rizzi, M.; Pastore, A.; Pascente, R.; Liang, L.-P.; Villa, B.R.; Balosso, S.; Abramov, A.Y.; van Vliet, E.A.; Del Giudice, E.; Aronica, E.; Patel, M.; Walker, M.C.; Vezzani, A. Targeting oxidative stress improves disease outcomes in a rat model of acquired epilepsy. *Brain* **2019**, *142*, e39, doi:10.1093/brain/awz130.

189. Tallarico, M.; Leo, A.; Guarnieri, L.; Zito, M.C.; De Caro, C.; Nicoletti, F.; Russo, E.; Constanti, A.; De Sarro, G.; Citraro, R. N-acetylcysteine aggravates seizures while improving depressive-like and cognitive impairment comorbidities in the WAG/Rij rat model of absence epilepsy. *Mol. Neurobiol.* **2022**, *59*, 2702–2714, doi:10.1007/s12035-021-02720-3.

190. Hurd, R.W.; Wilder, B.J.; Helveston, W.R.; Uthman, B.M. Treatment of four siblings with progressive myoclonus epilepsy of the Unverricht-Lundborg type with N-acetylcysteine. *Neurology* **1996**, *47*, 1264–1268, doi:10.1212/wnl.47.5.1264.

191. Edwards, M.J.J.; Hargreaves, I.P.; Heales, S.J.R.; Jones, S.J.; Ramachandran, V.; Bhatia, K.P.; Sisodiya, S. N-acetylcysteine and Unverricht-Lundborg disease: variable response and possible side effects. *Neurology* **2002**, *59*, 1447–1449, doi:10.1212/wnl.59.9.1447.

192. Singh, N.; Vijayanti, S.; Saha, L.; Bhatia, A.; Banerjee, D.; Chakrabarti, A. Neuroprotective effect of Nrf2 activator dimethyl fumarate, on the hippocampal neurons in chemical kindling model in rat. *Epilepsy Res.* **2018**, *143*, 98–104, doi:10.1016/j.eplepsyres.2018.02.011.

193. Singh, N.; Saha, L.; Kumari, P.; Singh, J.; Bhatia, A.; Banerjee, D.; Chakrabarti, A. Effect of dimethyl fumarate on neuroinflammation and apoptosis in pentylenetetrazol kindling model in rats. *Brain Res. Bull.* **2019**, *144*, 233–245, doi:10.1016/j.brainresbull.2018.11.013.

194. Xia, L.; Liu, L.; Cai, Y.; Zhang, Y.; Tong, F.; Wang, Q.; Ding, J.; Wang, X. Inhibition of Gasdermin D-Mediated Pyroptosis Attenuates the Severity of Seizures and Astroglial Damage in Kainic Acid-Induced Epileptic Mice. *Front. Pharmacol.* **2021**, *12*, 751644, doi:10.3389/fphar.2021.751644.

195. Sandouka, S.; Singh, P.K.; Saadi, A.; Taiwo, R.O.; Sheeni, Y.; Zhang, T.; Deeb, L.; Guignet, M.; White, S.H.; Shekh-Ahmad, T. Repurposing dimethyl fumarate as an antiepileptogenic and disease-modifying treatment for drug-resistant epilepsy. *J. Transl. Med.* **2023**, *21*, 796, doi:10.1186/s12967-023-04695-2.

196. Chang, Y.; Zhou, M.; Zhang, R.Y. Antioxidant effect of dimethyl fumarate in pentylenetetrazole-kindled epilepsy mice and is activated by nuclear factor erythroid 2-related factor 2 pathway. *J. Physiol. Pharmacol.* **2024**, *75*, doi:10.26402/jpp.2024.1.07.

197. Pinar, N. Effects of Tempol on Epileptic Activity in Picrotoxin-Induced Epilepsy in Rats. *Neurol. India* **2021**, *69*, 426–429, doi:10.4103/0028-3886.314542.

198. Ho, Y.-H.; Lin, Y.-T.; Wu, C.-W.J.; Chao, Y.-M.; Chang, A.Y.W.; Chan, J.Y.H. Peripheral inflammation increases seizure susceptibility via the induction of neuroinflammation and oxidative stress in the hippocampus. *J. Biomed. Sci.* **2015**, *22*, 46, doi:10.1186/s12929-015-0157-8.

199. Dubey, V.; Dixit, A.B.; Banerjee, J. Tempol suppresses generalized seizures in animal model of epilepsy. *Neurol. India* **2023**, *71*, 412–413, doi:10.4103/0028-3886.375411.

200. Rong, Y.; Doctrow, S.R.; Tocco, G.; Baudry, M. EUK-134, a synthetic superoxide dismutase and catalase mimetic, prevents oxidative stress and attenuates kainate-induced neuropathology. *Proc Natl Acad Sci USA* **1999**, *96*, 9897–9902, doi:10.1073/pnas.96.17.9897.

201. Singh, N.; Serres, F.; Toker, L.; Sade, Y.; Blackburn, V.; Batra, A.S.; Saiardi, A.; Agam, G.; Belmaker, R.H.; Sharp, T.; Vasudevan, S.R.; Churchill, G.C. Effects of the putative lithium mimetic ebselen on pilocarpine-induced neural activity. *Eur. J. Pharmacol.* **2020**, *883*, 173377, doi:10.1016/j.ejphar.2020.173377.

202. Liu, X.; Wang, F.-Y.; Chi, S.; Liu, T.; Yang, H.-L.; Zhong, R.-J.; Li, X.-Y.; Gao, J. Mitochondria-targeting peptide SS-31 attenuates ferroptosis via inhibition of the p38 MAPK signaling pathway in the hippocampus of epileptic rats. *Brain Res.* **2024**, *1836*, 148882, doi:10.1016/j.brainres.2024.148882.

203. Ahmed, M.A.E. Neuroprotective effects of idebenone against pilocarpine-induced seizures: modulation of antioxidant status, DNA damage and Na(+), K (+)-ATPase activity in rat hippocampus. *Neurochem. Res.* **2014**, *39*, 394–402, doi:10.1007/s11064-014-1236-z.

204. Kaur, A.; Kaur, A.; Samriti; Goel, R.K. Attenuation of mitochondrial refractory epilepsy in rotenone corneal kindling model of drug resistance by idebenone: An approach to bypass mitochondrial complex I. *Epilepsy Res.* **2024**, *207*, 107458, doi:10.1016/j.eplepsyres.2024.107458.

205. Kahn-Kirby, A.H.; Amagata, A.; Maeder, C.I.; Mei, J.J.; Sideris, S.; Kosaka, Y.; Hinman, A.; Malone, S.A.; Bruegger, J.J.; Wang, L.; Kim, V.; Shrader, W.D.; Hoff, K.G.; Latham, J.C.; Ashley, E.A.; Wheeler, M.T.; Bertini, E.; Carrozzo, R.; Martinelli, D.; Dionisi-Vici, C.; Chapman, K.A.; Enns, G.M.; Gahl, W.; Wolfe, L.; Saneto, R.P.; Johnson, S.C.; Trimmer, J.K.; Klein, M.B.; Holst, C.R. Targeting ferroptosis: A novel therapeutic strategy for the treatment of mitochondrial disease-related epilepsy. *PLoS ONE* **2019**, *14*, e0214250, doi:10.1371/journal.pone.0214250.

206. Kayser, E.-B.; Mulholland, M.; Olkhova, E.A.; Chen, Y.; Coulson, H.; Cairns, O.; Truong, V.; James, K.; Johnson, B.M.; Hanaford, A.; Johnson, S.C. Evaluating the efficacy of vatiquinone in preclinical models of Leigh syndrome and GPX4 deficiency. *Orphanet J. Rare Dis.* **2025**, *20*, 65, doi:10.1186/s13023-025-03582-x.

207. Tchekalarova, J.; Petkova, Z.; Pechlivanova, D.; Moyanova, S.; Kortenska, L.; Mitreva, R.; Lozanov, V.; Atanasova, D.; Lazarov, N.; Stoynev, A. Prophylactic treatment with melatonin after status epilepticus: effects on epileptogenesis, neuronal damage, and behavioral changes in a kainate model of temporal lobe epilepsy. *Epilepsy Behav.* **2013**, *27*, 174–187, doi:10.1016/j.yebeh.2013.01.009.

208. Petkova, Z.; Tchekalarova, J.; Pechlivanova, D.; Moyanova, S.; Kortenska, L.; Mitreva, R.; Popov, D.; Markova, P.; Lozanov, V.; Atanasova, D.; Lazarov, N.; Stoynev, A. Treatment with melatonin after status epilepticus attenuates seizure activity and neuronal damage but does not prevent the disturbance in diurnal rhythms and behavioral alterations in spontaneously hypertensive rats in kainate model of temporal lobe epilepsy. *Epilepsy Behav.* **2014**, *31*, 198–208, doi:10.1016/j.yebeh.2013.12.013.

209. Goldberg-Stern, H.; Oren, H.; Peled, N.; Garty, B.-Z. Effect of melatonin on seizure frequency in intractable epilepsy: a pilot study. *J. Child Neurol.* **2012**, *27*, 1524–1528, doi:10.1177/0883073811435916.

210. Jain, S.V.; Horn, P.S.; Simakajornboon, N.; Beebe, D.W.; Holland, K.; Byars, A.W.; Glauser, T.A. Melatonin improves sleep in children with epilepsy: a randomized, double-blind, crossover study. *Sleep Med.* **2015**, *16*, 637–644, doi:10.1016/j.sleep.2015.01.005.

211. Maghbooli, M.; Alyan NajafAbadi, S.; MalekMahmoudi, G.; Molseghi, M.H. Effect of add-on melatonin on seizure outcomes and quality of sleep in epilepsy with idiopathic generalized tonic-clonic seizures alone in adult patients: Cross-sectional, randomized, double-blind, placebo-controlled clinical trial. *Brain Behav.* **2023**, *13*, e2860, doi:10.1002/brb3.2860.

212. Sun, Y.; Chen, J.; Shi, X.; Li, Z.; Wan, L.; Yan, H.; Chen, Y.; Wang, J.; Wang, J.; Zou, L.; Reiter, R.; Zhang, B.; Yang, G. Safety and efficacy of melatonin supplementation as an add-on treatment for infantile epileptic spasms syndrome: A randomized, placebo-controlled, double-blind trial. *J. Pineal Res.* **2024**, *76*, e12922, doi:10.1111/jpi.12922.
